# System-level assessment of dynamic reconfiguration for lifetime and cost outcomes in electric vehicle battery packs

**DOI:** 10.1038/s41467-026-74951-8

**Published:** 2026-07-08

**Authors:** Albert Škegro, Torsten Wik, Bo Bijlenga, Alexander Bessman, Changfu Zou

**Affiliations:** 1 Department of Electrical Engineering, Chalmers University of Technology, Gothenburg, Sweden; 2 PHINIA Inc., Åmål, Sweden; 3 Scania CV AB, Södertälje, Sweden

**Keywords:** Electrical and electronic engineering, Batteries

## Abstract

The electrification of transport relies heavily on lithium-ion batteries, yet conventional fixed-configuration battery packs suffer from structural inefficiencies such as cell-to-cell variability and premature failure. Dynamic battery reconfiguration, enabled by intelligent control of switching hardware around individual cells, provides an alternative pack architecture. Here we present a systematic, battery technology-agnostic evaluation of the lifetime and economic benefits of reconfigurable battery packs using a statistically grounded framework based on detailed cell modelling across diverse design and usage conditions. We show that reconfigurable battery packs can extend battery lifetime by over 20%, particularly in high-voltage applications such as electric trucks and long-range passenger vehicles. Despite higher upfront costs, reconfigurable packs can reduce lifetime cost by deferring replacements and retaining greater residual value. A sensitivity analysis identifies robust thresholds for economic viability: battery capacities above approximately 50 kWh, annual driving distances below 12,150 km, and additional upfront costs under 7.16%. These findings position dynamic reconfiguration as a scalable, cost-optimised architecture for next-generation battery platforms, and provide a quantitative foundation for future hardware design, management software, and life-cycle sustainability assessments.

## Abstract (web summary)

The study uses large-scale simulations and validated cell models to show that dynamic reconfiguration in electric vehicle battery packs extends lifetime and reduces costs, quantifying thresholds for economic benefit across chemistries, voltages, and usage patterns.

## Introduction

Contemporary transportation systems remain heavily dependent on fossil fuels, and the resulting tailpipe emissions are a major driver of ecosystem degradation and climate change^1^. Electric vehicle (EV) adoption, powered predominantly by lithium-ion batteries, is widely regarded as a key strategy to decarbonise transport and cut these emissions. However, meeting the high energy and power demands of EVs requires battery packs containing hundreds or thousands of cells in series/parallel configurations (e.g., on the order of 10^2^–10^4^ cells per pack in modern EV models), which raises new challenges in battery management and reliability. Despite ongoing efforts, battery management systems (BMS) have so far achieved only incremental improvements without fundamental breakthroughs^2^. Advanced, intelligent control systems for managing battery health, efficiency, and robustness are still in their infancy, leaving today’s BMS with limited ability to optimise longevity or life-cycle resource efficiency. Inevitable manufacturing variations and non-uniform operating conditions lead to cell-to-cell performance variability within packs. This heterogeneity means that the usable capacity and performance of a conventional battery pack (CBP) are effectively limited by its weakest cell^3^. Moreover, the premature degradation or failure (e.g., an internal short or open-circuit) of a single cell can disable an entire module or string, potentially causing complete pack failure and requiring costly replacement of the whole battery pack, even though most cells may still be fully operational. Such scenarios compromise, and in some cases even undermine, the economic viability and sustainability of large-scale electrified transport, as prematurely retired EV battery packs represent wasted embodied energy and materials.

**Fig. 1:**
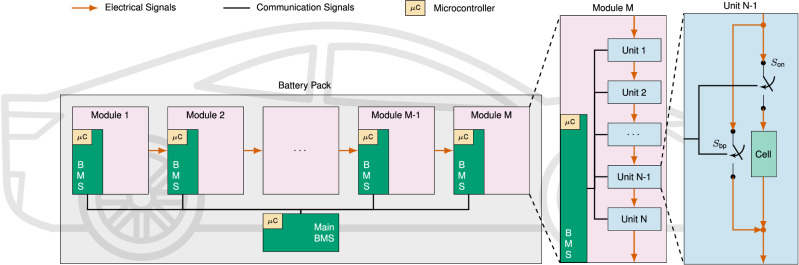
Physical architecture of a dynamically reconfigurable battery pack (RBP). Each reconfigurable battery unit consists of a cell and two controllable switches, enabling independent engagement or bypassing during operation. A detailed description of the hierarchical RBP design is provided in Supplementary Information S12.

Dynamic reconfiguration has emerged as a promising strategy to overcome these structural limitations of conventional, fixed-configuration battery packs (CBPs), which are unable to adapt to non-uniform cell degradation over time^4,5^. The physical architecture enabling this concept is illustrated in Fig. 1. By integrating power electronics, such as semiconductor switches around individual cells or cell groups, and employing advanced real-time control algorithms, reconfigurable battery packs (RBPs) can dynamically alter cell interconnections during operation^6,7^. This added flexibility enables more uniform utilisation of individual cells^8^, mitigation of weak-cell bottlenecks, and enhanced resilience to localised faults^9^. Nevertheless, these benefits come at the cost of increased hardware complexity, integration effort^10^, and upfront costs, factors that have contributed to scepticism about their broader practical viability^11^. A summary of key functional advantages and associated challenges is given in Supplementary Table S1. Despite these hurdles, leading automotive and battery manufacturers, including Audi^12^, Mercedes-Benz^13^, Porsche^14^, Scania^15^, Bosch^16^, Geely^17^, LG Chem^18^, and Samsung^19^, have actively engaged in research and development efforts, reflecting growing industry confidence in the concept’s practical value (see Supplementary Fig. S1). Yet, existing studies have primarily focused on immediate advantages such as active balancing or fault diagnosis, treating lifetime extension and cost savings as secondary benefits^20,21^. Furthermore, the available techno-economic assessments tend to be case-specific^22,23^ or architecture-limited^24^, lacking systematic evaluations that generalise across diverse degradation scenarios, environmental conditions, and usage profiles.

To address this critical gap, we present a systematic and battery technology-agnostic evaluation of the lifetime extension and economic implications enabled by dynamic reconfiguration. The analysis builds on large-scale virtual cell populations and experimentally validated battery models, combining equivalent-circuit electrical formulations with semi-empirical ageing models. A structured design-space exploration is conducted across a broad range of battery chemistries, pack topologies, usage intensities, and temperature distributions, reflecting the scale and diversity encountered in real-world EV deployments. To account for cell-to-cell manufacturing variability, each scenario is further evaluated using 1000 Monte Carlo simulations with stochastic variations in initial capacity and resistance, enabling systematic comparison between CBPs and RBPs. Notably, these results reveal that the achievable lifetime extension from dynamic reconfiguration scales nonlinearly with system voltage and is significantly influenced by battery chemistries. Building on these findings, we further quantify how lifetime gains translate into economic benefits through a system-level cost analysis, enabling identification of design, usage, and cost thresholds for economically viable deployment. Our results highlight substantial economic opportunities of RBPs, particularly for high-voltage and high-capacity EVs.

## Results

We evaluate the potential of dynamic battery reconfiguration across a representative set of realistic operating conditions and design scenarios. The analysis considers five categories of influencing factors: manufacturing variability, thermal environment, usage pattern, system voltage, and cell chemistry.

Manufacturing variability is characterised by cell-to-cell dispersion in beginning-of-life (BOL) capacity and resistance. Thermal environment is characterised by the long-term mean cell temperature and the degree of spatial temperature non-uniformity within the pack. Usage pattern is described by the fraction of time the battery remains at rest, system voltage by the number of series-connected cells, and cell chemistry by separate simulations of lithium iron phosphate (LFP) and lithium nickel manganese cobalt oxide (NMC) cells. Together, these factors span practically relevant EV design and operating regimes and are selected based on their relevance to battery pack design^25^, correspondence to real-world usage^26^, and physical links to dominant degradation mechanisms^27^. The definitions, symbols, investigated values, and scenario counts for these factors are summarised in Supplementary Table S2.

**Fig. 2:**
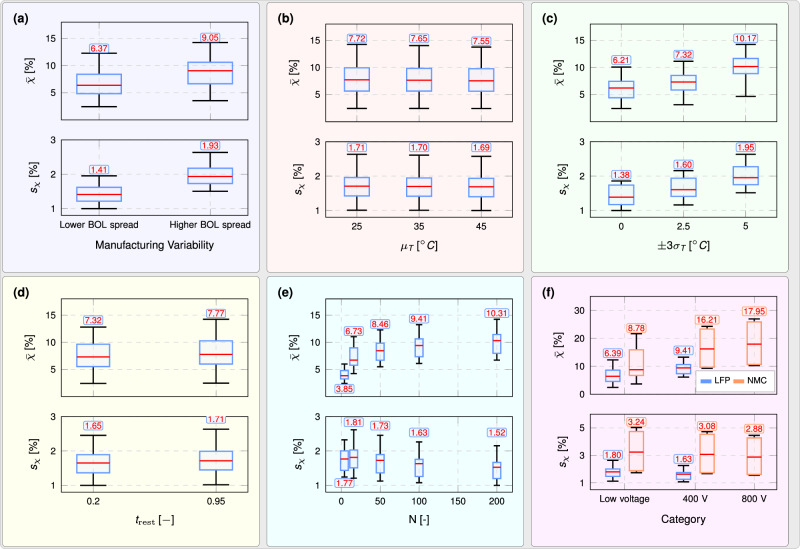
Sensitivity of lifetime extension to key system and environmental factors. **a** Cell manufacturing variability in beginning-of-life (BOL) capacity and resistance. **b** Mean operating cell temperature *μ*_*T*_. **c** Spatial temperature gradient *σ*_*T*_. **d** Rest period duration *t*_rest_. **e **The number of series-connected cells *N*. **f** Cell chemistry and system voltage, with blue boxes denoting lithium iron phosphate (LFP) and orange boxes denoting lithium nickel manganese cobalt oxide (NMC) cells. Each subfigure contains two panels: the top shows mean lifetime extension ($\bar{\chi }$) and the bottom shows its standard deviation (*s*_*χ*_), evaluated across all relevant scenarios. Median values are indicated above or below each boxplot. In all boxplots: center line, median; box limits, upper and lower quartiles; whiskers, 1.5 × interquartile range; points, outliers. Source data are provided as a Source Data file.

In total, 240 scenarios are evaluated: 216 for LFP and 24 for NMC, each corresponding to a unique combination of factor values (see Supplementary Information S3). To account for stochastic cell-to-cell variations at BOL, each scenario is executed using 1000 Monte Carlo simulations, yielding a distribution of lifetime extension values *χ*. From each distribution, two metrics are extracted: the mean lifetime extension $\bar{\chi }$ and its standard deviation *s*_*χ*_. Sensitivity analyses are then performed for each factor category, and the resulting distributions of $\bar{\chi }$ and *s*_*χ*_ are visualised using boxplots. Figure 2 presents the LFP results together with LFP–NMC comparisons, while detailed NMC-only results are provided in Supplementary Fig. S2. In each boxplot, scenarios sharing the same value of the factor under study are grouped together, while the remaining input factors are varied.

### Effect of manufacturing variability

Manufacturing inconsistencies, such as variations in electrode coating, material loading, porosity, and solid-electrolyte interphase (SEI) formation, are primary sources of cell-to-cell performance variability^28^. Many of these differences originate during electrode production and are further amplified during cell formation. In current manufacturing practice, post-formation grading is used to characterise individual cells based on metrics such as BOL cell capacity and resistance; afterwards, sorting is performed to group cells with similar properties for module assembly. While this two-step approach helps to mitigate cell variations, it incurs significant cost and processing time. Specifically, the formation stage alone, which requires charge-discharge cycling of every cell to form the SEI and assess initial performance, accounts for a substantial share of production time and energy use. The combination of this stage with post-formation grading and sorting can account for approximately 30% of total cell manufacturing costs and remains a major production bottleneck^29^. Even so, well-matched cells often diverge in performance over time, making perfect long-term uniformity practically unattainable.

To evaluate this effect, we compare two representative manufacturing-variability test cases (TC1: tighter tolerances; TC2: looser tolerances), with chemistry-specific parameter values (see Supplementary Table S2). As illustrated in Fig. 2a, both the mean lifetime extension $\bar{\chi }$ and its standard deviation *s*_*χ*_ tend to increase with manufacturing variability. Specifically, for LFP cells, the median $\bar{\chi }$ increases from 6.37% in TC1 to 9.05% in TC2, while the median *s*_*χ*_ rises from 1.41% to 1.94%. Importantly, the coefficient of variation remains comparable between TC1 and TC2 (22.14% for TC1 and 21.44% for TC2). These results confirm that dynamic reconfiguration generally provides greater benefits in more heterogeneous battery packs without introducing proportionally higher uncertainty in outcomes. Moreover, the findings support the intuition that dynamic reconfiguration is particularly advantageous in packs where one or a few weak (i.e., poorly manufactured or prematurely degraded) cells can disproportionately constrain pack-level performance. Although the manufacturing variability and inconsistent ageing are inherently stochastic, the trends observed in Fig. 2a highlight the robustness of reconfiguration in extending battery lifetime under realistic conditions.

These findings have practical relevance for both first-life and second-life applications. In first-life systems, dynamic reconfiguration does not eliminate the need for basic quality assurance, but it significantly reduces the reliance on costly screening and sorting procedures. This not only lowers processing costs but also expands the feasible supply of cells: batches with wider capacity spreads, which are typically cheaper due to relaxed grading requirements, can be effectively utilised without sacrificing long-term pack-level performance. By shifting the burden from front-loaded manufacturing precision to adaptive in-life control, it offers a scalable, cost-effective pathway to improve battery pack reliability and sustainability. In second-life applications, variability is further amplified by heterogeneous prior use, repurposing strategies, and ageing during the second life. Dynamic reconfiguration enables these battery packs to bypass underperforming cells and harness remaining capacity more effectively, thereby mitigating imbalance and extending functional lifetime.

### Effect of thermal environment

Across the studied thermal conditions, variation in the spatially averaged long-term cell temperature *μ*_*T*_ has only a minor negative effect on lifetime extension (Fig. 2b). Quantitatively, increasing *μ*_*T*_ from 25 °C to 45 °C slightly reduces the median expected extension $\bar{\chi }$, from 7.72% to 7.55%, with a marginal decrease in the median *s*_*χ*_ from 1.70% to 1.69%. These results suggest that moderate changes in the thermal setpoint, whether driven by ambient conditions or battery thermal management system (BTMS) settings, have limited impact on the benefit of reconfiguration. This minor reduction is not due to reduced cell-to-cell variability but instead stems from the shortened service life imposed by higher temperatures: as *μ*_*T*_ increases, overall degradation accelerates, shortening the pack’s service life and reducing the time window for reconfiguration to mitigate differential ageing.

In contrast, greater spatial temperature gradients *σ*_*T*_ consistently lead to increased $\bar{\chi }$ and *s*_*χ*_, as shown in Fig. 2c. As *σ*_*T*_ increases from 0 to 0.84 °C, corresponding to a full-pack spread of 3*σ*_*T*_ = ± 2.5 °C, the median $\bar{\chi }$ rises from 6.20% to 10.17%, and the median *s*_*χ*_ increases from 1.38% to 1.95%. This trend reflects the fact that spatial thermal variation induces divergent ageing trajectories: cells exposed to locally higher temperatures degrade faster, increasing capacity and resistance imbalances. Dynamic reconfiguration helps mitigate these effects by bypassing or isolating thermally overstressed cells, thereby improving overall pack longevity.

This behaviour highlights the potential for a combined thermal-electrical control strategy. The BTMS operates in the thermal domain, regulating heat via actuators such as pumps, fans, and heat exchangers, but it acts slowly and is constrained by physical design and cost^30–32^. Reconfiguration, by contrast, operates in the electrical domain where the control dynamics are much faster, enabling rapid redistribution of current at the cell level. Used together, these systems form a multi-tiered degradation mitigation strategy: the BTMS reduces the pack’s average temperature and global thermal imbalances on a slow timescale, while reconfiguration rapidly mitigates local temperature peaks and thermal gradients. These complementary effects are particularly useful when BTMS optimisation is limited by design trade-offs or economic constraints.

### Effect of usage pattern

The two utilisation regimes span the physical envelope of battery ageing behaviour: the low-utilisation case (*t*_rest_ = 0.95) is dominated by calendar ageing, while the high-utilisation case (*t*_rest_ = 0.2) is dominated by cycle ageing. Intermediate duty cycles are bounded by these two extremes. Variation in usage pattern, represented by the rest-time fraction, exerts only a modest influence on lifetime extension (Fig. 2d). Across the investigated utilisation regimes, the median of the mean lifetime extension $\bar{\chi }$ for LFP cells increases slightly, from 7.32% to 7.77%. The median of the standard deviation *s*_*χ*_ also rises marginally, from 1.65% to 1.71%. These results indicate that RBPs provide consistent benefits over CBPs across a wide range of duty cycles. Whether the battery is cycled intensively or remains idle for long durations, reconfiguration remains effective in extending lifetime, reinforcing its applicability across diverse EV usage scenarios, from intensively operated mining haulers to privately owned vehicles with extended idle periods.

### Effect of system voltage

Increasing system voltage, reflected in a larger number of series-connected cells (see Supplementary Table S2), increases the mean lifetime extension and reduces the variability of outcomes (Fig. 2e). Quantitatively, across the investigated voltage classes, the median $\bar{\chi }$ increases from 3.85% to 10.31%, while the median of *s*_*χ*_ decreases from 1.76% to 1.52%. This indicates that higher-voltage battery architectures not only achieve greater expected lifetime extension, but also exhibit less variation in outcomes, yielding more consistent and predictable performance. This trend can be explained by contrasting the structural limitations of CBPs with the flexibility of RBPs. In a CBP, increasing the number of series-connected cells increases the probability that at least one cell will exhibit significant capacity or resistance mismatch, or reach the end of life (EOL) prematurely. Because CBPs lack the ability to bypass individual cells, failure of a single weak cell can trigger early pack-level EOL. In contrast, RBPs allow selective bypassing or isolation of underperforming cells, preventing premature pack failure. As the number of series-connected cells increases, so does the flexibility of reconfiguration, enhancing the pack’s overall robustness and lifetime.

These findings have direct implications for EV system design. Although dynamic reconfiguration provides lifetime extension across all pack sizes, its relative advantage scales with system voltage. High-voltage architectures, such as those used in hybrid electric vehicles (HEVs) and battery electric vehicles (BEVs), stand to benefit the most. While low-voltage systems (e.g., mild hybrids or auxiliary applications) can still gain from reconfiguration, the absolute improvements in lifetime extension are more limited.

### Effect of chemistry

To evaluate whether the observed effects of reconfiguration generalise across battery chemistries, a subset of matched test scenarios is investigated using NMC cells, with the temperature fixed at 25 °C. Manufacturing variability exerts a strong influence in NMC-based packs: the median of the mean lifetime extension $\bar{\chi }$ increases from 7.86% in TC1 to 18.75% in TC2, with a corresponding rise in the median standard deviation *s*_*χ*_ from 1.84% to 4.62% (see Supplementary Fig. S2a and Fig. S2b). This trend aligns with the behaviour observed in LFP systems and reinforces the principle that reconfiguration becomes more effective when BOL cell-to-cell heterogeneity is more pronounced. Moreover, these findings suggest that reconfiguration is particularly effective in NMC-based systems, yielding notably higher lifetime extension compared to LFP. However, the observed median *s*_*χ*_ = 3.06% is also higher than in LFP under equivalent conditions. This elevated variability arises from more pronounced divergence in ageing trajectories among NMC cells, potentially driven by their greater thermal sensitivity compared to LFP cells.

The influence of usage pattern on NMC cells is also examined. Increasing *t*_rest_ from 0.2 to 0.95 leads to a modest decline in lifetime extension, from 10.56% to 9.76%, accompanied by a reduction in *s*_*χ*_ (see Supplementary Fig. S2c). This contrasts slightly with the positive trend seen in LFP and likely reflects chemistry-specific differences in dominant degradation mechanisms, particularly NMC’s greater susceptibility to calendar ageing.

System voltage is stratified into three levels: low voltage, 400 V, and 800 V (see Fig. 2f). In both the low and 400 V categories, NMC exhibits consistently higher median values of $\bar{\chi }$ than LFP (8.78% vs. 6.39%, and 16.21% vs. 9.41%, respectively). The corresponding median values of *s*_*χ*_ are also higher for NMC (3.24% vs. 1.80%, and 3.08% vs. 1.63%), although both chemistries show a decreasing trend of *s*_*χ*_ with increasing voltage. For 800 V systems, the lifetime extension reaches a particularly pronounced median of 17.95%, with a standard deviation of 2.88%. These results align with those observed in LFP systems, showing that higher voltages tend to yield greater lifetime extension and lower scenario-to-scenario variation.

### Implications for real-world EV applications

While the individual dependencies on manufacturing variability, thermal environment, usage pattern, and chemistry are analysed separately in the preceding subsections, their combined system-level implications cannot be directly inferred. Here, we synthesise these factors by projecting the resulting lifetime-extension behaviour onto real-world EV architectures, thereby enabling an application-level interpretation of reconfiguration benefits. Specifically, the expected lifetime extension through dynamic reconfiguration is mapped onto 20 representative EV models spanning 15 manufacturers. These vehicles, characterised by their nominal battery pack voltages and cell chemistries, represent a cross-section of mild hybrid electric cars, low-voltage electric cars, high-voltage electric cars, and electric commercial trucks. System voltage is adopted as a compact system-level proxy that reflects multiple coupled design attributes, including the number of series-connected cells, pack power level, and the statistical averaging of cell-to-cell heterogeneity. This abstraction captures the dominant scaling effects of battery pack configuration without introducing a high-dimensional design space.

**Fig. 3:**
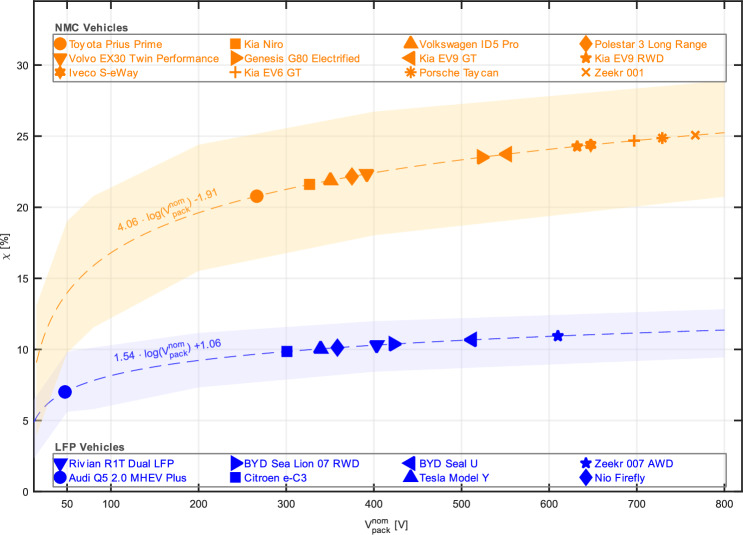
Estimated mean lifetime extension ($\bar{\chi }$) enabled by dynamic reconfiguration across representative electric vehicle (EV) models. Models are characterised by cathode chemistry, i.e., lithium iron phosphate (LFP) or lithium nickel manganese cobalt oxide (NMC), and nominal pack voltage ${V}_{\,{{\rm{pack}}}}^{{{\rm{nom}}}\,}$. Simulations are performed under a representative baseline usage and thermal context; scenario mapping details and fit evaluation are provided in the Supplementary Information. Real-world EV models are mapped onto fitted curves according to voltage class and chemistry^68^. MHEV, mild hybrid electric vehicle; BEV, battery electric vehicle. Source data are provided as a Source Data file.

The results, shown in Fig. 3, reveal a clear and interpretable pattern: the benefit of reconfiguration increases with system voltage, but not linearly. Instead, the gain follows a logarithmic trend, rising steeply at first, then tapering off, which reflects the diminishing marginal utility of increasing voltage from a reconfiguration perspective. For instance, mild hybrid electric vehicles (MHEVs), such as the Audi Q5 (nominal voltage: 47.6 V), fall near the lower end of the voltage spectrum and show modest lifetime gains ($\bar{\chi }\approx 7\%$). Mainstream BEVs, such as the Tesla Model Y and BYD Sea Lion (300-400 V), are associated with moderate extensions (~10%), while high-voltage platforms, such as the Porsche Taycan, Kia EV9, and Iveco S-eWay truck (500-750 V), achieve substantial benefits exceeding 24%. Moreover, for any given voltage level, NMC-based vehicles consistently outperform their LFP-based counterparts. This is consistent with earlier findings (see the Effect of Chemistry subsection), reflecting the stronger reconfiguration gains achievable in chemistries with greater intrinsic ageing variability.

These findings underscore that dynamic reconfiguration is particularly well-suited to high-voltage EV architectures, where the large number of series-connected cells amplifies the achievable lifetime extension. Vehicle classes such as long-range BEVs, performance-oriented EVs, and electric commercial trucks typically fall into this high-voltage regime and therefore exhibit substantial technical benefits in terms of lifetime extension. Nevertheless, even in lower-voltage platforms, the integration of reconfigurable battery systems can yield meaningful improvements in battery longevity, thereby offering sustainability and cost-of-ownership benefits. More broadly, this case study demonstrates that dynamic reconfiguration is a chemistry-agnostic and architecture-adaptive approach to enhancing battery lifetime.

### Economic impact of RBPs

While the preceding subsection establishes the technical lifetime-extension potential of dynamic reconfiguration, its practical adoption ultimately depends on economic viability. In particular, the extent to which lifetime extension translates into cost benefits depends on usage intensity and economic conditions.

Although RBPs require higher upfront investments due to added hardware, they may reduce lifetime costs by mitigating degradation, deferring battery replacements, and retaining higher residual value. To quantify this trade-off, we compare the net present cost (NPC) of RBPs and CBPs, accounting for five components: upfront investment, operation and maintenance (O&M), energy-related losses, battery replacements, and residual value. Building upon the results of lifetime extension (see Eq. (18) in the Methods section), we conduct a sensitivity analysis of the lifetime cost difference across eight parameters to assess how design, usage, and economic factors influence the lifetime cost benefit of RBPs (*Δ*NPC, defined in Eq. (28)). These include factors controllable through engineering and investment decisions, alongside real-world usage and market conditions. Two usage factors capture how the EV is operated: vehicle lifetime (*Y*_EV_), representing different deployment horizons, and annual driving distance (*L*), accounting for differences in driving intensity. Economic factors include the RBP upfront cost factor (*ν*), defined as the relative increase in initial cost of RBPs compared to CBPs, the discount rate (*r*), and O&M cost rates for CBPs (*α*_CBP_) and RBPs (*α*_RBP_), where the latter is lower due to reduced maintenance requirements for RBPs. Design factors include the relative increase in ohmic losses (*δ*_loss_), representing additional energy losses introduced by RBP power electronics. We also vary the nominal battery pack energy (${E}_{\,{{\rm{pack}}}}^{{{\rm{nom}}}\,}$), which determines the battery pack sizing, and consider cell chemistry as a categorical parameter, consistent with the scenarios in the Effect of Chemistry subsection. Full parameter ranges and sampling details are provided in the Specification and Implementation subsection.

**Fig. 4:**
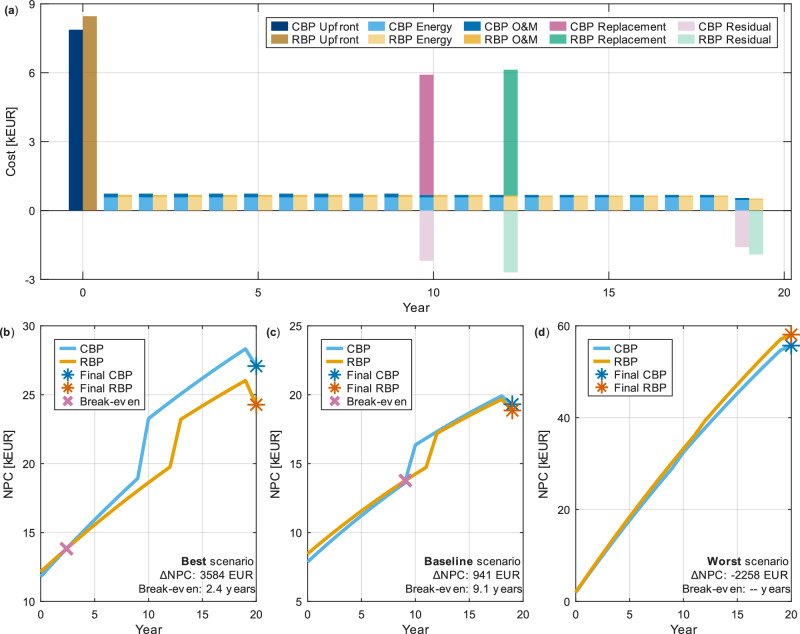
Cost composition and cumulative net present cost trajectories of reconfigurable battery packs (RBPs) relative to conventional battery packs (CBPs). A positive *Δ*NPC indicates lifetime cost savings for the RBP relative to the CBP; NPC, net present cost. **a** Annual cost breakdown in the baseline scenario. **b–d** Cumulative NPC trajectories in the most favourable scenario (**b**), baseline scenario (**c**), and least favourable scenario (**d**). All parameter details are provided in Supplementary Table S5. Source data are provided as a Source Data file.

The annual cost breakdown of the CBP and RBP in the baseline scenario (80 kWh, LFP pack, EV lifetime of 18.8 years; full parameter details provided in Supplementary Table S5) is shown in Fig. 4a. Compared to the CBP, the RBP incurs higher upfront and replacement costs due to the additional power electronics required for dynamic reconfiguration. These electronics also introduce ohmic losses and modestly increase the RBP’s energy cost. However, the RBP compensates for these added expenses through two key mechanisms: postponing battery replacements and retaining greater residual value. Specifically, the RBP delays the replacement interval by 1.1 years. Since annual costs are tracked at full-year resolution, this shift causes the replacement event to appear in the following calendar year, resulting in a change two years later in the cost profile (Fig. 4a). Additionally, the RBP’s slower degradation leads to a residual value that is more than 19% higher than that of the CBP.

In the baseline scenario, cost parity between the RBP and CBP architectures is achieved after 9.1 years, before the battery replacement (Fig. 4c). By the EV end-of-life, the RBP yields a total saving of €941. This corresponds to 5.61% of the CBP lifetime cost and 11.95% of the CBP upfront cost. Lifetime cost outcomes range from substantial savings to net penalties. Under favourable design and usage conditions, dynamic reconfiguration reaches cost break-even with the conventional design after 2.4 years, saving €3584 (Fig. 4b), equivalent to 15.65% of the CBP lifetime cost and 30.36% of the CBP upfront cost. In the least favourable scenario (Fig. 4d), the additional cost of implementing dynamic reconfiguration is never recovered, and the RBP incurs €2258 more than the CBP. These results demonstrate that the economic viability of RBPs is context-dependent, rather than universally beneficial. Full parameter sets for the most favourable and least favourable cases are provided in Supplementary Table S5.

**Fig. 5:**
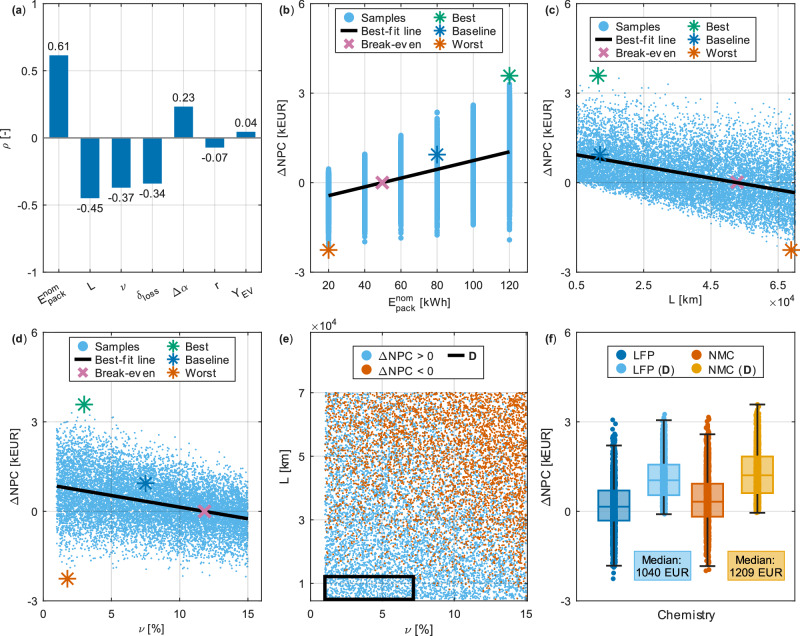
Sensitivity analysis of the lifetime cost benefit of reconfigurable battery packs (RBPs) (*Δ*NPC). NPC, net present cost; CBP, conventional battery pack. **a** Global sensitivity ranking based on the Spearman rank correlation coefficient (*ρ*) between input parameters and *Δ*NPC. *ν*, RBP upfront cost factor; ${E}_{\,{{\rm{pack}}}}^{{{\rm{nom}}}\,}$, nominal battery pack energy; *L*, annual driving distance; *δ*_loss_, relative increase in ohmic losses; *α*_CBP_, CBP operation-and-maintenance cost rate; *Y*_EV_, electric vehicle lifetime; *r*, discount rate. One-dimensional sensitivities of *Δ*NPC to ${E}_{\,{{\rm{pack}}}}^{{{\rm{nom}}}\,}$(**b**), *L*(**c**), and ν(**d**). Points show individual simulation samples. **e** Design space analysis in the (*ν*, *L*) plane. The identified rectangular region (D) indicates the favourable design region where at least 99.7% of cases yield *Δ*NPC > 0. **f** Sensitivity to cell chemistry. Boxplots show the distribution of *Δ*NPC for lithium iron phosphate (LFP) and lithium nickel manganese cobalt oxide (NMC) chemistries; `D' refers to samples within the favourable design region from panel (**e**). In all boxplots: center line, median; box limits, upper and lower quartiles; whiskers, 1.5 × interquartile range; points, outliers. Source data are provided as a Source Data file.

Based on the sensitivity analysis, the most influential driver of economic viability is the nominal battery pack energy ${E}_{\,{{\rm{pack}}}}^{{{\rm{nom}}}\,}$ (Spearman correlation coefficient *ρ* = 0.61, Fig. 5a; see the method in Supplementary Information S9). While Fig. 5a includes all investigated sensitivity parameters, we focus the discussion on the three most influential factors. Extended visualisations and scenario analyses for the remaining parameters are provided in Supplementary Fig. S3.

The nominal battery pack energy ${E}_{\,{{\rm{pack}}}}^{{{\rm{nom}}}\,}$ emerges as the most influential parameter in the lifetime cost comparison. This is because it directly and linearly scales the largest cost components within the economic model: upfront cost, replacement cost, and residual value (see Eq. (13)). While larger battery capacities entail higher upfront and replacement costs, they simultaneously amplify the financial benefits of lifetime extension through delayed replacements and enhanced residual value. A linear regression of the NPC difference, *Δ*NPC, against ${E}_{\,{{\rm{pack}}}}^{{{\rm{nom}}}\,}$ (Fig. 5b) reveals a cost break-even threshold at ${E}_{\,{{\rm{pack}}}}^{{{\rm{nom}}}\,}=49.72\,{{{\rm{kWh}}}}$. Below this threshold, RBPs are unlikely to achieve a lifetime cost benefit relative to CBPs. This implies that the economic viability of dynamic reconfiguration improves with larger pack sizes, highlighting substantial opportunities for long-range passenger EVs and heavy-duty EVs.

Compared to ${E}_{\,{{\rm{pack}}}}^{{{\rm{nom}}}\,}$, other parameters exert relatively less influence on the lifetime cost benefit. The annual driving distance *L* shows a negative correlation with *Δ*NPC (*ρ* = − 0.47; Fig. 5a). As *L* increases, energy costs rise for both the CBP and RBP; however, the additional power electronics in the RBP introduce slightly higher ohmic losses, reducing its lifetime cost benefit at higher driving intensities. A linear regression of *Δ*NPC against *L* (Fig. 5c) identifies a cost break-even threshold at *L* = 52,748 km. Nonetheless, substantial variability across scenarios indicates limited overall sensitivity. Most real-world EVs operate significantly below this break-even threshold. For instance, in Sweden, private vehicles drive on average 11,410 km annually, while light- and heavy-duty EVs average 13,030 km and 40,060 km, respectively^33^. These usage levels suggest that RBPs can be economically beneficial across a wide spectrum of real-world EV applications.

The RBP upfront cost factor *ν* also exhibits a modest negative correlation with *Δ*NPC (*ρ* = − 0.37; Fig. 5a). This is expected, as higher values of *ν* represent larger initial hardware investments in the RBP, which increase the upfront cost and reduce the likelihood of achieving a lifetime cost benefit. Regression analysis of *Δ*NPC against *ν* (Fig. 5d) places the cost break-even threshold at *ν* = 11.82%, above which RBPs are less likely to yield an economic gain over CBPs. As dynamic reconfiguration technology progresses and economies of scale reduce component costs, *ν* is expected to keep decreasing, further improving the long-term economic outlook for RBPs.

To provide actionable insights for RBP implementation and evaluate trade-offs between economic and usage factors, we identify a region in the (*ν*, *L*) space where RBPs consistently yield a lifetime cost benefit. Specifically, we search for the largest normalised rectangle in which at least 99.7% of samples result in *Δ*NPC > 0, regardless of variations in other parameters (Fig. 5e). Notably, we find that for *ν* below approximately 7.16% and annual driving distances under 12,150 km, RBPs deliver cost benefits across nearly all tested scenarios. This region lies well within the break-even thresholds identified individually for *ν* and *L*, thereby providing a robust and conservative implementation guideline.

Figure 5f compares the lifetime cost benefits of RBPs across LFP and NMC chemistries. In all scenarios, the median values of *Δ*NPC are positive, and both chemistries exhibit similar distributions of *Δ*NPC, reflecting equivalent unit cost assumptions (see Eq. (14) in the Methods section). When analysis is restricted to the favourable design region identified in Fig. 5e, median savings increase to €1040 for LFP and €1209 for NMC, with maximum values exceeding €3200 and €3500, respectively. The increased cost benefit observed for NMC-based RBPs is primarily driven by their greater lifetime extension compared to LFP counterparts, as quantified in the Effect of Chemistry subsection.

To assess robustness with respect to regional variation in electricity price and vehicle energy consumption, targeted sensitivity analyses are conducted across three representative European levels for each parameter, with results reported in Supplementary Fig. S5 and Supplementary Table S6. For large-pack applications (${E}_{\,{{\rm{pack}}}}^{{{\rm{nom}}}\,}\ge 50\,{{{\rm{kWh}}}}$), the fraction of economically favourable scenarios remains majority-positive across the full range of tested conditions, whereas small-pack viability is more sensitive to both parameters.

## Discussion

### Reconfiguration granularity

Full cell-level reconfiguration is considered in the analysis presented in the Results section, whereby each cell can be independently engaged or bypassed (Fig. 1). This configuration represents an upper bound on achievable performance, as it maximises the resolution at which cell-to-cell heterogeneity can be identified and acted upon during operation.

In practice, industrial research and pre-commercial demonstrator studies have predominantly explored coarser granularities, such as cluster- or string-level reconfiguration, as a compromise between performance gains, hardware complexity, packaging constraints, and system reliability^13,15,16,34^. At these scales, heterogeneity is managed in aggregated form, limiting the ability to isolate localised degradation or transient stress. This trade-off highlights a fundamental dependency between reconfiguration granularity and heterogeneity resolution. As granularity coarsens, the system progressively transitions from fine-grained adaptive control toward averaged management, reducing its capacity to mitigate weak-cell effects and redistribute degradation pathways. Consequently, the achievable lifetime extension and associated economic value are intrinsically linked to the architectural level at which heterogeneity can be resolved.

These considerations suggest that reconfiguration should not be viewed as a binary design choice, but rather as a continuum defined by architectural resolution. Identifying the optimal granularity under realistic cost, packaging, and reliability constraints will therefore be important for practical RBP implementation.

### Economic modelling framework and assumptions

The economic analysis relies on a set of static, transparent assumptions to enable consistent comparison between conventional and reconfigurable battery pack architectures across a wide design and usage space. These include a linear mapping between remaining state of health (SOH) and residual value, a uniform distribution to represent uncertainty in lifetime extension, and a fixed range of ohmic loss penalties associated with reconfiguration hardware (see details in the Methods section). Such assumptions are commonly adopted in system-level techno-economic assessments where the objective is to identify governing trends, break-even thresholds, and design envelopes rather than to predict application-specific market outcomes.

In practice, second-life battery value also depends on the internal condition of individual cells at retirement, which cannot be fully represented by static SOH-based proxies. While emerging diagnostic and data-driven approaches can provide more detailed characterisation of these conditions^35^, their integration into system-level economic analysis would require additional assumptions about second-life deployment, usage profiles, and market structures. Consequently, the adopted economic model serves as a baseline for capturing first-order effects of lifetime extension and deferred replacement, while enabling consistent comparison across diverse system configurations.

Second-life deployment pathways vary between direct pack-level reuse and cell-level harvesting followed by sorting and repackaging^36^. RBPs may fundamentally shift second-life strategies from pre-deployment disassembly and requalification toward in-operation management of degradation. By reducing cell-to-cell heterogeneity and enabling fault-tolerant operation through selective bypassing, they increase the technical feasibility of direct pack-level reuse and open pathways that are currently limited for CBPs. However, the realisation of this potential depends on factors beyond the battery itself, including certification frameworks (e.g., UL 1974^37^), system integration standards, and liability structures, which may still necessitate conservative processing workflows. As these external constraints are not explicitly modelled, the residual value estimated in this work may underestimate the benefits of RBPs in scenarios where pack-level reuse becomes viable.

### Alternative design strategies

Several engineering approaches have been proposed to mitigate the pack-level consequences of cell-to-cell heterogeneity and premature failure, acting at the stages of manufacturing, design, operation, or maintenance. However, these approaches intervene through fundamentally different mechanisms, and therefore should not be regarded as equivalent solutions to the same problem.

At the manufacturing stage, tighter cell sorting and grading aim to reduce BOL dispersion in capacity and resistance before pack assembly. This can improve initial uniformity, but, as discussed in the Manufacturing Variability subsection, it requires additional processing and cannot prevent cell states from diverging progressively during operation. At the design stage, battery oversizing mitigates premature EOL by introducing excess nominal capacity. Although straightforward to implement, this strategy increases material use and pack mass, while leaving the intrinsic weak-cell sensitivity of a fixed series-connected architecture unchanged.

At the operation stage, conventional battery management strategies such as passive or active balancing can redistribute charge and reduce short-term SOC imbalance^5^. Yet these strategies still act within a fixed electrical topology. They may smooth transient non-uniformity, but they cannot remove the structural bottleneck imposed by weak or degraded cells, nor can they reconfigure current paths in response to evolving cell-level limitations. Maintenance-stage interventions, such as modular replacement, restore performance only after degradation has already become sufficiently pronounced to justify service action.

Dynamic reconfiguration is distinct because it addresses heterogeneity through topology itself. Rather than only reducing the symptoms of non-uniform ageing, it changes how non-uniformity is propagated, tolerated, and managed at pack level during operation. Its distinguishing feature is therefore not merely improved balancing, but the ability to convert a structurally rigid battery pack into one that can adapt electrically to evolving cell conditions. Viewed in this context, dynamic reconfiguration is best understood as an architectural response to heterogeneity, whereas oversizing, balancing, sorting, and replacement each address only specific manifestations or stages of the problem. This difference in intervention level helps explain why reconfiguration opens a broader pathway for degradation-aware battery pack design.

### Beyond EV applications

While the analysis is presented in the context of EV operation, the underlying framework is not specific to a particular application domain. By separating model structure from parameterisation, it can be extended to systems with different duty cycles, power profiles, and operational constraints, including stationary energy storage^38,39^. In such settings, reconfigurable architectures may provide similar advantages in terms of utilisation efficiency and robustness to cell-level degradation. However, the extent to which these benefits are realised depends on application-specific constraint regimes. Stationary systems, for example, are subject to distinct safety, installation, and certification requirements (e.g., UL 1973^40^, UL 9540^41^, NFPA 855^42^), as well as different integration and liability considerations.

These factors influence not only system design, but also acceptable operating strategies and failure tolerances, which in turn affect the practical value of reconfiguration. Therefore, extending the present framework to other domains requires careful reparameterisation under domain-specific constraints, rather than direct transfer of EV-based results.

### Concluding remarks

This study establishes a robust quantitative framework for assessing dynamic battery reconfiguration as an effective strategy for lifetime extension and cost reduction in EV battery systems. By integrating large-scale stochastic simulations with detailed cell electrical and degradation modelling, we show that RBPs systematically overcome the intrinsic limitations of CBPs, particularly their vulnerability to performance bottlenecks caused by cell-to-cell variability.

Reconfiguration delivers broad lifetime extension across architectures, chemistries, and usage profiles, with its magnitude scaling nonlinearly with system voltage. High-voltage EV platforms exhibit the greatest lifetime improvements, owing to their higher number of series-connected cells and increased susceptibility to localised failures. Moreover, thermal gradients and manufacturing-induced heterogeneities further amplify the advantages of dynamic reconfiguration, positioning it as a fast-timescale complement to conventional BTMS strategies. Both LFP and NMC chemistries benefit, with NMC showing greater gains due to its stronger sensitivity to imbalance-induced degradation.

Our economic analysis reveals that dynamic reconfiguration can deliver notable lifetime cost savings under realistic EV deployment scenarios. These savings stem primarily from deferred battery replacements and improved residual value. However, the economic outcome depends not only on the achievable lifetime extension but also on operational factors. A global sensitivity analysis highlights nominal battery pack energy as the most decisive factor, identifying a threshold of approximately 50 kWh above which RBPs are substantially more likely to outperform CBPs in the overall system cost. Annual driving distance and upfront hardware cost exert secondary but still meaningful influence. Importantly, we delineate a conservative design-use region, covering packs below 7.16% incremental cost and vehicles driven under 12,150 km per year, within which RBPs are almost universally cost-effective. These thresholds serve as concrete, actionable guidelines for implementation.

Taken together, our results demonstrate that dynamic reconfiguration is not merely a supplementary feature but a system-level design strategy that enables longer-lasting, more resilient, and economically viable battery systems. With continued advancements in switching hardware and integration strategies, the cost penalty of reconfiguration is likely to diminish, further widening its margin of advantage. This positions RBPs as a scalable enabler of sustainable and cost-efficient electrified transport, especially for high-performance and commercial applications. Future work should focus on translating the structural advantages of reconfiguration into practical battery management strategies, particularly by accounting for switching behaviour and its system-level effects.

## Methods

We consider two types of battery pack architectures: CBPs and RBPs. A CBP, as examined in this study, comprises a series connection of *N* battery cells permanently wired in a fixed topology. In certain real-world applications, multiple cells are connected in parallel prior to the series configuration, forming what is commonly referred to as a battery unit. In such cases, each parallel-connected unit can be treated as a single effective cell in this study without loss of generality. In contrast, the basic building block of an RBP is the reconfigurable battery unit, which consists of a battery cell and a pair of controllable switches (see Fig. 1). One switch is connected in series and the other in parallel with the cell, enabling the unit to selectively engage or bypass the cell during operation. Two cell types are selected for evaluation: the Sony Murata US26650FTC1 (LFP chemistry)^43^ and the Sanyo UR18650E (NMC chemistry)^44^. Both use graphite as the anode material. The LFP cell employs lithium iron phosphate as the cathode active material, while the NMC cell features a nickel-manganese-cobalt oxide cathode in a 1:1:1 ratio. Cell specifications are provided in Supplementary Tables S8 and S9.

### Modelling of battery cell behaviour

The electrical and ageing models are adopted from experimentally validated formulations reported in the literature, without re-identification of parameters. Detailed parameter values are provided in Supplementary Information S10.

The same electrical model structure is employed for both LFP and NMC cells. This model comprises an open-circuit voltage (OCV), a series resistance *R*_0_, and a single resistor-capacitor (RC) pair consisting of a polarisation resistance *R*_1_ and a capacitance *C*_1_. The OCV is modelled as a function of the state of charge (SOC), while *R*_0_, *R*_1_, and *C*_1_ are temperature-dependent. The OCV and ECM parameters are taken from^45^ for LFP cells and^46,47^ for NMC cells.

The ageing model captures two primary degradation mechanisms: capacity fade and resistance increase. Both components are modelled separately for LFP and NMC chemistries using semi-empirical expressions derived in ^48–50^. The total capacity loss ${\widetilde{Q}}_{i}^{l}$ and resistance increase ${\widetilde{R}}_{i}^{l}$ of the *i*^th^ cell comprise contributions from both calendar ageing and cycle ageing, modelled additively:

1a $${\widetilde{Q}}_{i}^{l}={\widetilde{Q}}_{i}^{l,\,{{\rm{cal}}}\,}+{\widetilde{Q}}_{i}^{l,\,{{\rm{cyc}}}\,}\,,\quad i=1,\ldots,{N}_{s}\,,$$

1b $${\widetilde{R}}_{i}^{l}={\widetilde{R}}_{i}^{l,\,{{\rm{cal}}}\,}+{\widetilde{R}}_{i}^{l,\,{{\rm{cyc}}}\,}\,,\quad i=1,\ldots,{N}_{s}\,.$$

Here, ${\widetilde{Q}}^{l,{{\rm{cal}}}}$ and ${\widetilde{R}}^{l,{{\rm{cal}}}}$ denote the degradation contributions from calendar ageing, i.e. those occurring irrespective of cycling activity, while ${\widetilde{Q}}^{l,{{\rm{cyc}}}}$ and ${\widetilde{R}}^{l,{{\rm{cyc}}}}$ encompass the degradation effects resulting from repeated charge and discharge cycles during operation.

The RMS SOC over a given cycle for cell *i*, denoted by SOC^*ϕ*^, quantifies the quadratic mean of the SOC levels throughout the cycle duration, starting at ${t}_{\,{{\rm{cyc}}}}^{{{\rm{s}}}\,}$ and ending at ${t}_{\,{{\rm{cyc}}}}^{{{\rm{e}}}\,}$:

2 $${\,{{\rm{SOC}}}}_{i}^{\phi }=\sqrt{\frac{1}{{t}_{{{{\rm{cyc}}}}}^{{{{\rm{e}}}}}-{t}_{{{{\rm{cyc}}}}}^{{{{\rm{s}}}}}}\int_{{t}_{{{{\rm{cyc}}}}}^{{{{\rm{s}}}}}}^{{t}_{{{{\rm{cyc}}}}}^{{{\rm{e}}}\,}}{\left[{{{\rm{SOC}}}}_{i}\left(t\right)\right]}^{2}dt}\,.$$

For LFP cells, calendar ageing is modelled as a function of the cell’s RMS SOC, temperature *T*, and time *t*, following the semi-empirical relations proposed in^48^:

3a $${\widetilde{Q}}_{i}^{l,\,{{\rm{cal}}}}({{{\rm{SOC}}}}_{i}^{\phi },{T}_{i},t)={k}_{{{\rm{SOC}}},{{\rm{Q}}}}({{{\rm{SOC}}}}_{i}^{\phi })\cdot {k}_{{{\rm{T}}},{{\rm{Q}}}}({T}_{i})\cdot {t}^{0.5}\,,$$

3b $${\widetilde{R}}_{i}^{l,\,{{\rm{cal}}}}({{{\rm{SOC}}}}_{i}^{\phi },{T}_{i},t)={k}_{{{\rm{SOC}}},{{\rm{R}}}}({{{\rm{SOC}}}}_{i}^{\phi })\cdot {k}_{{{\rm{T}}},{{\rm{R}}}}({T}_{i})\cdot t\,,$$

where

4a $${k}_{{{\rm{SOC}}},{{\rm{Q}}}}({{{\rm{SOC}}}\,}_{i}^{\phi })={\gamma }_{1}{\left({{{\rm{SOC}}}}_{i}^{\phi }-{\gamma }_{2}\right)}^{3}+{\gamma }_{3}\,,$$

4b $${k}_{{{\rm{T}}},{{\rm{Q}}}}({T}_{i})={\gamma }_{4}\,\exp \left(-\frac{{\gamma }_{5}}{{\gamma }_{6}}\left(\frac{1}{{T}_{i}}-\frac{1}{{\gamma }_{7}}\right)\right)\,,$$

4c $${k}_{{{\rm{SOC}}},{{\rm{R}}}}({{{\rm{SOC}}}\,}_{i}^{\phi })={\gamma }_{8}{\left({{{\rm{SOC}}}}_{i}^{\phi }-{\gamma }_{9}\right)}^{2}+{\gamma }_{10}\,,$$

4d $${k}_{{{\rm{T}}},{{\rm{R}}}}({T}_{i})={\gamma }_{11}\,\exp \left(-\frac{{\gamma }_{12}}{{\gamma }_{13}}\left(\frac{1}{{T}_{i}}-\frac{1}{{\gamma }_{14}}\right)\right)\,,$$

with parameters *γ*_1_, …, *γ*_14_ fitted to experimental data.

The cycle ageing of LFP cells is modelled as a function of the cell’s C-rate, depth of discharge (DOD), and equivalent full cycles (EFCs), following^49^:

5a $${\widetilde{Q}}_{i}^{l,\,{{\rm{cyc}}}}({C}_{i}^{{{{\rm{rate}}}}},{{{\rm{DOD}}}}_{i},{{{\rm{EFC}}}}_{i})={k}_{{{\rm{C}}},{{\rm{Q}}}}({C}_{i}^{{{{\rm{rate}}}}})\cdot {k}_{{{\rm{DOD}}},{{\rm{Q}}}}({{{\rm{DOD}}}}_{i})\cdot {{{\rm{EFC}}}\,}_{i}^{0.5}\,,$$

5b $${\widetilde{R}}_{i}^{l,\,{{\rm{cyc}}}}({C}_{i}^{{{{\rm{rate}}}}},{{{\rm{DOD}}}}_{i},{{{\rm{EFC}}}}_{i})={k}_{{{\rm{C}}},{{\rm{R}}}}({C}_{i}^{{{{\rm{rate}}}}})\cdot {k}_{{{\rm{DOD}}},{{\rm{R}}}}({{{\rm{DOD}}}}_{i})\cdot {{{\rm{EFC}}}}_{i}\,,$$

where

6a $${k}_{{{\rm{C}}},{{\rm{Q}}}}({C}_{i}^{{{{\rm{rate}}}}})={\gamma }_{15}{C}_{i}^{{{\rm{rate}}}\,}+{\gamma }_{16},$$

6b $${k}_{{{\rm{DOD}}},{{\rm{Q}}}}({{{\rm{DOD}}}}_{i})={\gamma }_{17}{\left({{{\rm{DOD}}}}_{i}-{\gamma }_{18}\right)}^{3}+{\gamma }_{19}\,,$$

6c $${k}_{{{\rm{C}}},{{\rm{R}}}}({C}_{i}^{{{{\rm{rate}}}}})={\gamma }_{20}{C}_{i}^{{{\rm{rate}}}\,}+{\gamma }_{21}\,,$$

6d $${k}_{{{\rm{DOD}}},{{\rm{R}}}}({{{\rm{DOD}}}}_{i})={\gamma }_{22}{\left({{{\rm{DOD}}}}_{i}-{\gamma }_{23}\right)}^{3}+{\gamma }_{24},$$

with parameters *γ*_15_, …, *γ*_24_ fitted to experimental data.

For NMC cells, calendar ageing is modelled using a semi-empirical expression derived from^50^, which incorporates the effects of the cell’s mean SOC ($\overline{\,{{\rm{SOC}}}\,}$), temperature, and time:

7a $${\widetilde{Q}}_{i}^{l,\,{{\rm{cal}}}\,}\left({\overline{{{\rm{SOC}}}}}_{i},{T}_{i},t\right)={k}_{{{\rm{SOC}}},{{\rm{Q}}}}\left({\overline{{{\rm{SOC}}}}}_{i}\right)\cdot {k}_{{{\rm{T}}},{{\rm{Q}}}}\left({T}_{i}\right)\cdot {t}^{0.75}\,,$$

7b $${\widetilde{R}}_{i}^{l,\,{{\rm{cal}}}\,}\left({\overline{{{\rm{SOC}}}}}_{i},{T}_{i},t\right)={k}_{{{\rm{SOC}}},{{\rm{R}}}}\left({\overline{{{\rm{SOC}}}}}_{i}\right)\cdot {k}_{{{\rm{T}}},{{\rm{R}}}}\left({T}_{i}\right)\cdot {t}^{0.75}\,,$$

where

8a $${k}_{{{\rm{SOC}}},{{\rm{Q}}}}\left({\overline{{{\rm{SOC}}}}}_{i}\right)={\beta }_{1}{v}_{{{{\rm{OC}}}}}\left({\overline{{{\rm{SOC}}}}}_{i}\right)+{\beta }_{2}\,,$$

8b $${k}_{{{\rm{T}}},{{\rm{Q}}}}({T}_{i})={\beta }_{3}\,\exp \left(-\frac{{\beta }_{4}}{{T}_{i}}\right)\,,$$

8c $${k}_{{{\rm{SOC}}},{{\rm{R}}}}\left({\overline{{{\rm{SOC}}}}}_{i}\right)={\beta }_{5}{v}_{{{{\rm{OC}}}}}\left({\overline{{{\rm{SOC}}}}}_{i}\right)+{\beta }_{6}\,,$$

8d $${k}_{{{\rm{T}}},{{\rm{R}}}}({T}_{i})={\beta }_{7}\,\exp \left(-\frac{{\beta }_{8}}{{T}_{i}}\right)\,,$$

with parameters *β*_1_, …, *β*_8_ fitted to experimental data.

The cycle ageing for NMC cells is also modelled based on^50^, considering the dependency on the cell’s RMS SOC, DOD, and cumulative capacity throughput (*Q*^th^):

9a $${\widetilde{Q}}_{i}^{l,\,{{\rm{cyc}}}\,}\left({\,{{\rm{SOC}}}}_{i}^{\phi },{{{\rm{DOD}}}}_{i},{Q}_{i}^{{{\rm{th}}}\,}\right)=\left[{k}_{{{\rm{SOC}}},{{\rm{Q}}}}\left({\,{{\rm{SOC}}}}_{i}^{\phi }\right)+{k}_{{{\rm{DOD}}},{{\rm{Q}}}}\left({{{\rm{DOD}}}}_{i}\right)\right]{({Q}_{i}^{{{{\rm{th}}}}})}^{0.5}\,,$$

9b $${\widetilde{R}}_{i}^{l,\,{{\rm{cyc}}}\,}\left({\,{{\rm{SOC}}}}_{i}^{\phi },{{{\rm{DOD}}}}_{i},{Q}_{i}^{{{\rm{th}}}\,}\right)=\left[{k}_{{{\rm{SOC}}},{{\rm{R}}}}\left({\,{{\rm{SOC}}}}_{i}^{\phi }\right)+{k}_{{{\rm{DOD}}},{{\rm{R}}}}\left({{{\rm{DOD}}}}_{i}\right)\right]{Q}_{i}^{{{\rm{th}}}\,}\,,$$

where

10a $${k}_{{{{\rm{SOC,Q}}}}}({{{\rm{SOC}}}\,}_{i}^{\phi })={\beta }_{9}{\left({v}_{{{{\rm{OC}}}}}({{{\rm{SOC}}}}_{i}^{\phi })+{\beta }_{10}\right)}^{2}\,,$$

10b $${k}_{{{{\rm{DOD,Q}}}}}({{{\rm{DOD}}}}_{i})={\beta }_{11}+{\beta }_{12}{{{\rm{DOD}}}}_{i}\,,$$

10c $${k}_{{{{\rm{SOC,R}}}}}({{{\rm{SOC}}}\,}_{i}^{\phi })={\beta }_{13}{\left({v}_{{{{\rm{OC}}}}}({{{\rm{SOC}}}}_{i}^{\phi })+{\beta }_{14}\right)}^{2}\,,$$

10d $${k}_{{{{\rm{DOD,R}}}}}({{{\rm{DOD}}}}_{i})={\beta }_{15}+{\beta }_{16}{{{\rm{DOD}}}}_{i}\,.$$

### Statistics of lifetime extension

For a CBP, the EOL time is defined as the point at which the first cell reaches 80% of its nominal capacity. In contrast, full reconfiguration in RBPs under an idealised control policy assumed in this work enables cells to be actively managed such that their long-term degradation is balanced and they reach the EOL simultaneously. The resulting lifetime therefore represents an upper bound on achievable lifetime extension. The analysis operates at the structural level, characterising the long-term effects of reconfiguration on cell utilisation and lifetime without resolving fast-timescale switching dynamics or controller-level decision logic. With this in mind, each RBP simulation continues until all cells drop below the 80% capacity threshold. At that point, the times at which individual cells cross the 80% threshold are recorded, along with the corresponding EFCs for each cell. For the RBP, the total EFC is computed as the average of the recorded EFCs across all cells at their respective EOL. The comparison between the total EFCs of the CBP and RBP provides a quantitative measure of lifetime extension. This extension is expressed as a percentage increase for simulation run *k*:

11 $${\chi }^{k}=\left(\frac{{\sum }_{i=1}^{N}{{{\rm{EFC}}}}_{i}}{N\cdot \min \{{{{\rm{EFC}}}}_{1},\ldots,{{{\rm{EFC}}}}_{N}\}}-1\right)\cdot 100\,[\%]\,,$$

where EFC_*i*_ denotes the EFCs when cell *i* reaches its EOL.

To summarise the overall performance across multiple simulation runs within each scenario defined in the Results section, we calculate the sample mean and sample variance of the lifetime improvements:

12a $$\bar{\chi }=\frac{1}{{N}_{{{{\rm{sim}}}}}}\mathop{\sum }_{k=1}^{{N}_{{{{\rm{sim}}}}}}{\chi }^{k}\,,$$

12b $${s}_{\chi }^{2}=\frac{1}{{N}_{{{{\rm{sim}}}}}-1}{\sum }_{k=1}^{{N}_{{{{\rm{sim}}}}}}{\left({\chi }^{k}-\bar{\chi }\right)}^{2}\,,$$

where *N*_sim_ denotes the total number of Monte Carlo simulations, which is set to 1000 for each scenario in this study.

### Economic cost modelling

For both the CBP and RBP, the NPC model accounts for the upfront cost ${C}_{\xi }^{\,{{\rm{init}}}\,}$, operational and maintenance (O&M) costs ${C}_{\xi }^{{{{\rm{O}}}}\,{\&}\,{{{\rm{M}}}}}$, energy costs ${C}_{\xi }^{\,{{\rm{en}}}\,}$, battery replacement costs ${C}_{\xi }^{\,{{\rm{repl}}}\,}$, and residual value ${C}_{\xi }^{\,{{\rm{res}}}\,}$, where *ξ* ∈ {CBP,  RBP}. The economic analysis assumes continued vehicle operation and does not explicitly model stochastic vehicle retirement events (e.g., accidents), which are exogenous to battery system design. The total NPC over the EV lifetime *Y*_EV_ for technology *ξ* is given by:

13 $${{{\rm{NPC}}}}_{\xi }={C}_{\xi }^{{{{\rm{init}}}}}+{\sum }_{y=1}^{{Y}_{{{{\rm{EV}}}}}}\left({C}_{y,\xi }^{\,{{\rm{en}}}\,}+{C}_{y,\xi }^{{{{\rm{O}}}}\,{\&}\,{{{\rm{M}}}}}+{C}_{y,\xi }^{\,{{\rm{repl}}}}\right)\frac{1}{{(1+r)}^{y}}-{C}_{\xi }^{{{\rm{res}}}\,},$$

where *y* denotes the year index, and *r* is the discount rate reflecting the time value of money.

The upfront cost of the CBP, ${C}_{\,{{\rm{CBP}}}}^{{{\rm{init}}}\,}$, is computed as the product of the nominal battery pack energy ${E}_{\,{{\rm{pack}}}}^{{{\rm{nom}}}\,}$ [kWh] and the cost per unit pack energy *c*^pack^ [EUR/kWh]:

14 $${C}_{\,{{\rm{CBP}}}}^{{{{\rm{init}}}}}={E}_{{{{\rm{pack}}}}}^{{{{\rm{nom}}}}}\cdot {c}^{{{{\rm{pack}}}}}.$$

To account for the additional investment required for reconfiguration hardware, such as controllable switches, connectors, and printed circuit boards (PCBs), the upfront cost of the RBP, ${C}_{\,{{\rm{RBP}}}}^{{{\rm{init}}}\,}$, is modelled as a scaled multiple of the upfront CBP cost ${C}_{\,{{\rm{CBP}}}}^{{{\rm{init}}}\,}$:

15 $${C}_{\,{{\rm{RBP}}}}^{{{\rm{init}}}\,}=\left(1+\nu \right)\cdot {C}_{\,{{\rm{CBP}}}}^{{{\rm{init}}}\,}\,,$$

where *ν* > 0 denotes the RBP upfront cost factor.

The annual energy cost of the CBP, ${C}_{y,\,{{\rm{CBP}}}}^{{{\rm{en}}}\,}$, is computed based on the vehicle’s specific energy consumption *ε* [kWh/km] and the annual driving distance *L* [km]:

16 $${C}_{y,\,{{\rm{CBP}}}}^{{{{\rm{en}}}}}={c}^{{{{\rm{en}}}}}\cdot \varepsilon \cdot L,$$

where *c*^en^ is the electricity price [EUR/kWh]. The RBP architecture introduces additional ohmic losses due to the presence of controllable switches in the current path (see Fig. 1). These losses occur even when a cell is disengaged, as at least one switch remains active in the current path. These energy losses do not directly affect cell ageing or lifetime extension in our simulations. Instead, we model the increased energy consumption of the RBP using the ohmic losses increase parameter *δ*_loss_:

17 $${C}_{y,\,{{\rm{RBP}}}}^{{{{\rm{en}}}}}=(1+{\delta }_{{{{\rm{loss}}}}})\cdot {C}_{y,{{\rm{CBP}}}}^{{{\rm{en}}}\,}\,.$$

Battery pack replacements typically occur at the end of each battery life *Y*_*ξ*_, which differs between the CBP and RBP. Building upon the results of lifetime extension, we model the lifetime extension achieved by RBP as a uniform distribution:

18 $$\frac{{Y}_{{{{\rm{RBP}}}}}-{Y}_{{{{\rm{CBP}}}}}}{{Y}_{{{{\rm{CBP}}}}}} \sim {{{\mathcal{U}}}}\left({\bar{\chi }}_{{{{\rm{med}}}}}-{s}_{\chi,{{\rm{med}}}},\ {\bar{\chi }}_{{{{\rm{med}}}}}+{s}_{\chi,{{\rm{med}}}}\right)\,,$$

where ${\bar{\chi }}_{{{{\rm{med}}}}}$ and *s*_*χ*,med_ denote the median values of $\bar{\chi }$ and *s*_*χ*_ defined in (12), and ${{{\mathcal{U}}}}({a}_{1},\,{a}_{2})$ represents a continuous uniform distribution over the interval [*a*_1_,  *a*_2_]. According to Fig. 3, both ${\bar{\chi }}_{{{{\rm{med}}}}}$ and *s*_*χ*,med_ are obtained from the nominal battery pack voltage ${V}_{\,{{\rm{pack}}}}^{{{\rm{nom}}}\,}$.

The replacement cost is time-dependent and reflects the projected decline in lithium-ion battery prices over time. It is modelled using an exponential decay function fitted to observed price data from 2018 to 2024^51^, and extrapolated to estimate future cost trends. In year *y*, the replacement cost per kilowatt-hour in USD, ${c}_{\,{{\rm{USD}}}}^{{{\rm{repl}}}\,}$, is given by:

19 $${c}_{\,{{\rm{USD}}}}^{{{\rm{repl}}}\,}(y)={p}_{1}\exp \left(-{p}_{2}({y}_{{{{\rm{init}}}}}-{y}_{0})\right)+{p}_{3},$$

where *y*_init_ = 2025 + *y* is the calendar year when the replacement occurs, and *y*_0_ = 2017 is the reference year. Parameter values obtained from the exponential fit are *p*_1_ = 172.0648, *p*_2_ = 0.1420, and *p*_3_ = 63.0950. The resulting cost is converted from USD/kWh to EUR/kWh using the European Central Bank (ECB) currency exchange rate of 1 USD = 0.8554 EUR^52^ at the time of analysis:

20 $${c}_{\,{{\rm{EUR}}}}^{{{\rm{repl}}}\,}(y)=0.8554\,{c}_{\,{{\rm{USD}}}}^{{{\rm{repl}}}\,}(y)$$

The replacement cost for CBP is now given as

21 $${C}_{y,\,{{\rm{CBP}}}}^{{{\rm{repl}}}\,}=\left\{\begin{array}{ll}0,\hfill&\,{{\rm{if}}}y\ne {Y}_{{{{\rm{CBP}}}}}\\ {c}_{\,{{\rm{EUR}}}}^{{{\rm{repl}}}\,}(y)\,\cdot {E}_{\,{{\rm{pack}}}}^{{{\rm{nom}}}\,},&\,{{\rm{if}}}y={Y}_{{{{\rm{CBP}}}}}\end{array}\right.$$

For the RBP, the replacement cost in the corresponding year is modelled as a scaled multiple of the CBP replacement cost:

22 $${C}_{y,\,{{\rm{RBP}}}}^{{{\rm{repl}}}\,}=\left(1+\nu \right)\cdot {C}_{y,\,{{\rm{CBP}}}}^{{{\rm{repl}}}\,}.$$

Note that, for both architectures, battery replacements are scheduled at the beginning of the calendar year immediately following the end of the battery pack lifetime.

The O&M cost includes routine inspections, hardware servicing, and software updates. These costs are modelled as a fixed fraction of the battery pack’s upfront cost^53^, denoted by the O&M rate *α*_*ξ*_. In year *y*, the relevant O&M cost is calculated based on the capital cost of the battery pack currently in service, denoted by ${C}_{\xi }^{\,{{\rm{capital}}}\,}(y)$:

23 $${C}_{y,\xi }^{{{{\rm{O}}}}\,{\&}\,{{{\rm{M}}}}}={\alpha }_{\xi }\cdot {C}_{\xi }^{\,{{\rm{capital}}}\,}(y).$$

In years without a battery replacement, ${C}_{\xi }^{\,{{\rm{capital}}}\,}(y)$ corresponds to the last battery pack acquisition cost, which is either the upfront cost ${C}_{\xi }^{\,{{\rm{init}}}\,}$ or the latest replacement cost ${C}_{y,\xi }^{\,{{\rm{repl}}}\,}$. In years with a replacement, the O&M cost is split proportionally between the old and new battery pack costs, weighted by the fraction of the year each system is in operation:

24 $${C}_{y,\xi }^{{{{\rm{O}}}}\,{\&}\,{{{\rm{M}}}}}={\alpha }_{\xi }\cdot \left({{{\rm{frac}}}}_{{{{\rm{old}}}}}\cdot {C}_{y,\xi }^{{{{\rm{O}}}}\&{{{\rm{M}}}},{{{\rm{old}}}}}+{{{\rm{frac}}}}_{{{{\rm{new}}}}}\cdot {C}_{y,\xi }^{{{\rm{O}}}\&{{\rm{M,new}}}\,}\right),$$

where frac_old_ and frac_new_ represent the fractions of the year operated with the old and the new battery packs, respectively, and ${C}_{y,\xi }^{\,{{\rm{O}}}\&{{\rm{M,old}}}\,}$ and ${C}_{y,\xi }^{\,{{\rm{O}}}\&{{\rm{M,new}}}\,}$ are their respective costs.

At the end of the battery pack lifetime *Y*_*ξ*_ and EV lifetime *Y*_EV_, the battery pack retains a residual value based on its remaining SOH. We model this by assuming linear degradation of SOH from 100% to a predefined end of the first life (EOL1) for *Y*_*ξ*_, which we set to SOH_EOL1_ = 80%.

Let *y*_elapsed_ denote the time elapsed since the most recent battery pack replacement. The remaining SOH at *y*_elapsed_ corresponds to

25 $${\,{{\rm{SOH}}}}_{{{{\rm{res}}}}}^{\xi }({y}_{{{{\rm{elapsed}}}}})=1-\frac{{y}_{{{{\rm{elapsed}}}}}}{{Y}_{\xi }}\cdot \left(1-{{{\rm{SOH}}}}_{{{{\rm{EOL1}}}}}\right).$$

The non-discounted residual value of the battery pack ${C}_{\xi }^{\,{{\rm{res,nd}}}\,}$ is then modelled as a fraction of the capital cost of the battery currently in service ${C}_{\xi }^{\,{{\rm{capital}}}\,}$:

26 $${C}_{\xi }^{\,{{\rm{res,nd}}}}({y}_{{{{\rm{elapsed}}}}})=	 \frac{{{{\rm{SOH}}}}_{{{{\rm{res}}}}}^{\xi }({y}_{{{{\rm{elapsed}}}}})-{{{\rm{SOH}}}}_{{{{\rm{EOL2}}}}}^{\xi }}{1-{{{\rm{SOH}}}}_{{{{\rm{EOL2}}}}}^{\xi }}\cdot {C}_{\xi }^{{{{\rm{capital}}}}}({y}_{{{{\rm{elapsed}}}}})\\ 	 \cdot {c}^{{{{\rm{res}}}}}\,,\quad \xi \in \{\,{{\rm{CBP}}},{{\rm{RBP}}}\,\}\,,$$

where *c*^res^ is a resale value coefficient capturing market-based depreciation for second-life batteries. The first factor on the right-hand side of Eq. (26) represents second-life potential, where the remaining SOH is normalised relative to the end of final life, SOH_EOL2_.

The residual value, discounted back to the present value, is then given by:

27 $${C}_{\xi }^{\,{{\rm{res}}}}({y}_{{{{\rm{elapsed}}}}})={C}_{\xi }^{{{{\rm{res,nd}}}}}({y}_{{{{\rm{elapsed}}}}})\cdot \frac{1}{{(1+r)}^{{y}_{{{{\rm{elapsed}}}}}}}\,.$$

Finally, the economic advantage of reconfiguration is expressed as the NPC difference:

28 $$\Delta \,{{\rm{NPC}}}={{{\rm{NPC}}}}_{{{{\rm{CBP}}}}}-{{{\rm{NPC}}}}_{{{{\rm{RBP}}}}}.$$

A positive *Δ*NPC indicates that the RBP achieves a lower total cost than the CBP over the full system lifetime.

### Specification and implementation

#### Cell manufacturing variability

For each of the considered battery chemistries, manufacturing variability is represented by four distribution parameters: the mean and standard deviation of BOL cell capacity (*μ*_*Q*_, *σ*_*Q*_) and of BOL cell resistance (*μ*_*R*_, *σ*_*R*_). Two sets of parameters are used, corresponding to two test cases. The corresponding parameter values are provided in Supplementary Information S11.

#### Temperature-related parameters

Prolonged exposure to significant temperature variations can cause divergence in cell capacity and internal resistance, adversely affecting the performance and consistency of a battery pack. To ensure optimal operation, a BTMS must control the maximum cell temperature and minimise temperature differences between cells. In a well-functioning BTMS, the maximum cell temperature is typically maintained below 50 °C, with the temperature difference between cells usually limited to 5 °C^54^. The temperature distribution across cells in both CBP and RBP architectures is approximated as a normal distribution:

29 $${T}_{i} \sim {{{\mathcal{N}}}}\left({\mu }_{T},\,{\sigma }_{T}^{2}\right),$$

where *T*_*i*_ represents the temperature of cell *i* in Kelvin (K). Here, *μ*_*T*_ represents the long-term mean cell temperature, defined as the spatial average across all cells in the battery pack, while *σ*_*T*_ quantifies the spatial temperature non-uniformity. In practice, this non-uniformity is small compared to the mean, i.e., *σ*_*T*_ ≪ *μ*_*T*_. While this approximation simplifies implementation and facilitates generalisation across various cell configurations and BTMS designs, it may underestimate the potential lifetime extension achievable through dynamic reconfiguration. In practice, an RBP could leverage reconfiguration capabilities to balance cell temperatures more effectively. Given that approximately 99.7% of data within a normal distribution lies within  ± 3*σ*_*T*_ of the mean *μ*_*T*_, we set 6*σ*_*T*_ ≈ 5 *K*, consistent with values reported in^54^, yielding *σ*_*T*_ = 0.84 *K*. The mean temperature *μ*_*T*_ is varied within [25, 35, 45]°C to represent both mild and relatively high-temperature environments.

#### Economic analysis parameterisation

In our parameter settings, the vehicle lifetime *Y*_EV_ varies from 15 to 20 years, aligning with recent estimates of BEV lifetime of 18.4 years^55^. The CBP lifetime *Y*_CBP_ is assumed to be 10 years, in line with increasingly prevailing industry practices^56^. The nominal EV battery pack voltage is set to 800 V, reflecting the adoption of next-generation BEVs. Based on the results in Fig. 3, this voltage value is first used to derive ${\bar{\chi }}_{{{{\rm{med}}}}}$ and *s*_*χ*,med_, which are subsequently substituted into (18) to compute the RBP lifetime *Y*_RBP_.

Annual driving distance *L* ranges from 5000 km to 70,000 km, reflecting averages for private cars (11,410 km/year) and commercial taxis (61,600 km/year) reported by Swedish transportation statistics^33^. Values for the nominal battery pack energy ${E}_{\,{{\rm{pack}}}}^{{{\rm{nom}}}\,}$ are selected from common EV sizes {20, 40, …, 120} kWh^57^.

To calculate the upfront cost of the CBP, we set the cost per unit pack energy to *c*^pack^ = 98.37 EUR/kWh, derived from a market-average price of 115 USD/kWh^51^ and the ECB currency exchange rate of 1 EUR = 1.1690 USD^52^ at the time of analysis. To estimate the upfront cost of the RBP, we perform a bottom-up, component-level analysis based on automotive-grade switches, gate drivers, microcontrollers, and circuit boards. This analysis suggests that reconfiguration hardware accounts for approximately 9.36% for LFP and 8.47% for NMC of the total pack cost in automotive applications (see Supplementary Information S12). Hence, to account for variation in implementation choices, we parameterise the RBP upfront cost factor *ν* to range between 1% and 15%. Annual energy costs are calculated using the vehicle’s specific energy consumption *ε* = 0.2 kWh/km, representative of mid- to large-size EVs under mixed driving conditions^58^. The electricity price is set to *c*^en^ = 0.24 EUR/kWh, corresponding to average household consumer prices in Sweden in the second half of 2024^59^.

To capture the additional ohmic losses introduced by switches in RBPs, while allowing for hardware design variation, we note that parallel connection of switches can reduce the effective switch resistance to less than 10% of a typical cell’s internal resistance^11^, limiting the added RBP energy losses to below 2%^60^. To also cover less favourable hardware implementations, we conservatively vary the ohmic losses increase parameter *δ*_loss_ ∈ [1%, 5%].

Discount rate follows the European Union’s recommended baseline of 3%^61^. To test the robustness of our findings, we vary the rate between 2% and 4%, reflecting a range of financial assumptions and risk profiles relevant to different stakeholders.

For battery energy storage systems (BESS), O&M rates between 0.5%^62^ and 2.5%^63^ of the upfront cost are reported. Due to more demanding operational conditions in EVs, for CBPs, we adopt *α*_CBP_ ∈ [1%, 3%]. For RBPs, SOH balancing and fault isolation are expected to reduce maintenance needs by mitigating degradation in the most stressed cells and simplifying service interventions. Accordingly, the O&M cost rate is set to 50% of the CBP value: *α*_RBP_ = 0.5 *α*_CBP_.

Regarding the resale value coefficient *c*^res^, which captures market-based depreciation for second-life batteries, estimates in the literature suggest that the market value of second-life EV batteries ranges from 20% to 80% of the cost of new batteries^64^. In our baseline scenario, we assume *c*^res^ = 0.5.

The end of final life, ${\,{{\rm{SOH}}}}_{{{\rm{EOL2}}}\,}^{\xi }$, is defined differently for CBPs and RBPs to account for their respective tolerances to cell-level degradation. For CBPs, the range of SOH_EOL2_ is set to 50–60%, in line with reported values for second-life applications^65^. In contrast, RBPs can dynamically bypass degraded cells, enabling continued operation at lower pack-level average SOH. Accordingly, a reduced range of 40–50% is applied.

To comprehensively assess the economic impact of RBPs, we generate 100,000 parameter combinations using Latin Hypercube Sampling (LHS). LHS is selected for its efficiency in exploring high-dimensional parameter spaces without requiring prohibitively large sample sizes^66^. Each parameter is varied within the ranges specified above, which are selected based on recent literature and market data as discussed in the corresponding paragraphs. Every sampled configuration is then used to compute the NPC difference (*Δ*NPC). A complete overview of parameter ranges used in the economic analysis is given in Supplementary Information S13.

#### Simulation platform and setup

This analysis is conducted using MATLAB via extensive simulation experiments on a Linux-based computer cluster with 40 cores. In these experiments, all cells are initialised at an SOC of 50%.

## Supplementary information

Supplementary Information

Transparent Peer Review file

## Source data

Source Data

## Data Availability

The source data underlying the figures in this paper are provided in the Source Data file. All simulation parameter values used in this study are provided in the Methods section and Supplementary Information.  Source data are provided with this paper.

## Code availability

The code used in this work is publicly available at Zenodo^67^.
