# Supplementary material for: System-level assessment of dynamic reconfiguration for lifetime and cost outcomes in electric vehicle battery packs
Source: Nat Commun. 2026 Jul 8;17:5980. doi: 10.1038/s41467-026-74951-8 (PMC13346987; doi:10.1038/s41467-026-74951-8)
Supplement: Supplementary file 1 — Supplementary Information [file 41467_2026_74951_MOESM1_ESM.pdf]

Supplementary Information

System-Level Assessment of Dynamic Reconfiguration for Lifetime and Cost Outcomes in Electric Vehicle Battery Packs

Albert Škegro[1], Torsten Wik[1], Bo Bijlenga[2], Alexander Bessman[3], Changfu Zou[1,*]

[1]Department of Electrical Engineering, Chalmers University of Technology, Gothenburg, 41296, Sweden
[2]PHINIA Inc., 662 21 Åmål, Sweden
[3]Scania CV AB, 151 87 Södertälje, Sweden

*Corresponding author: changfu.zou@chalmers.se

S1. Functional Properties, System-Level Outcomes, and Industrial Engagement

Supplementary Table S1 and Supplementary Fig. S1 complement Fig. 1 of the main text by summarising the functional properties, system-level outcomes, and challenges of dynamic battery reconfiguration, alongside a timeline of industrial engagement.

Supplementary Table S1 | Functional properties, system-level outcomes, and associated challenges of dynamic battery reconfiguration. Functional properties describe capabilities enabled directly by reconfiguration; system-level outcomes are the resulting benefits at the pack or vehicle level; challenges represent costs and drawbacks introduced by the additional hardware and control complexity.

Functional properties	System-level outcomes	Challenges
State of health balancing	Lifetime extension	Increased ohmic losses
Fault isolation	Faster charging	Increased upfront cost
Improved observability	Improved safety	Hardware complexity
Support for mixed chemistries		Integration effort

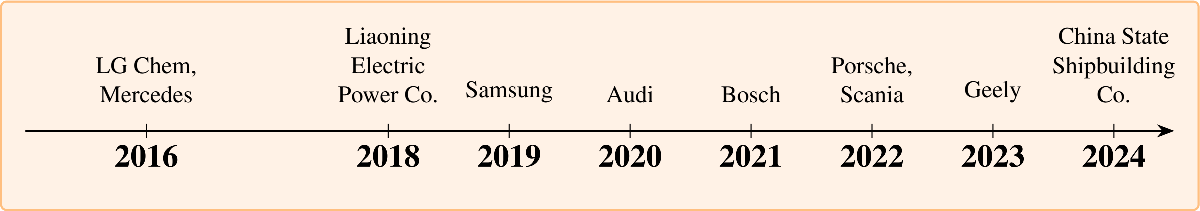

Supplementary Fig. S1 | Timeline of industrial engagement with dynamic battery reconfiguration. First known public disclosures by stakeholders across the automotive, battery manufacturing, grid utility, and heavy industry sectors between 2016 and 2024.

S2. Study Factors and Investigated Levels

Two cell chemistries are considered throughout: lithium iron phosphate (LFP) and lithium nickel manganese cobalt oxide (NMC). The table below consolidates the definitions and investigated levels of the five key influencing factors considered in the lifetime and economic analyses.

Supplementary Table S2 | Summary of influencing factors, symbols, and investigated levels. The table consolidates the definitions and investigated levels of the five key influencing factors considered in the lifetime and economic analyses. These parameters are varied across the simulation design space described in the Results section of the main text, while detailed modelling assumptions are provided in the Methods section of the main text. TC1 and TC2 denote the two manufacturing variability test cases defined in the Manufacturing Variability subsection of the main text, corresponding to tighter and looser production tolerances, respectively.

**Factor**	**Physical meaning**	**Symbols**	**Investigated levels**	**Scenarios**
Manufacturing variability	Cell-to-cell heterogeneity at beginning of life arising from production tolerances	CV_Q, CV_R	**LFP:**<br>TC1: $CV_Q = 0.23\%$, $CV_R = 1.81\%$<br>TC2: $CV_Q = 0.33\%$, $CV_R = 0.73\%$<br>**NMC:**<br>TC1: $CV_Q = 0.36\%$, $CV_R = 1.04\%$<br>TC2: $CV_Q = 0.80\%$, $CV_R = 1.94\%$	2 per chemistry
Thermal environment	Long-term average temperature and spatial non-uniformity within the battery pack	μ_T, σ_T	$\mu_T \in \{25, 35, 45\}$ °C<br>$\sigma_T \in \{0,\ 0.417,\ 0.833\}$ °C<br>(full-pack spread $\pm 3\sigma_T$)	9 (LFP only)
Usage pattern	Fraction of time the battery remains at rest (idle)	t_{rest}	$t_{\text{rest}} \in \{0.2,\ 0.95\}$	2
System voltage	Electrical architecture defined by number of series-connected cells	N	$N \in \{4,\ 14/16,\ 20,\ 50,\ 100,\ 200\}$<br>(14 for NMC, 16 for LFP at ~50 V)	6
Cell chemistry	Cathode material and associated ageing behaviour	—	LFP, NMC	2

S3. Lifetime Extension Analysis: Scenario Counts

Supplementary Table S3 reports the number of simulation scenarios underlying the boxplots in Fig. 2 of the main text. For each investigated category, the table specifies how many scenarios were used to generate each boxplot in the corresponding subfigure. Where relevant, separate counts are provided for LFP and NMC chemistries. For example, in Fig. 2a, each boxplot is based on 108 scenarios.

Supplementary Table S3 | Number of scenarios contributing to each subfigure in Fig. 2 of the main text.

Subfigure	Category	Boxplot	Scenarios per boxplot
**(a)**	Manufacturing variability	Each	108
**(b)**	Mean cell temperature μ_T	Each	72
**(c)**	Spatial temperature gradient σ_T	Each	72
**(d)**	Rest-time fraction t_{rest}	Each	108
**(e)**	Number of series-connected cells N	Each	36
**(f)**	Cell chemistry	LFP (low voltage)	144
		NMC (low voltage)	16
		LFP (400 V)	36
		NMC (400 V)	4
		NMC (800 V)	4
		**Total LFP**	**216**
		**Total NMC**	**24**

S4. Lifetime Extension Analysis: Supplementary Results

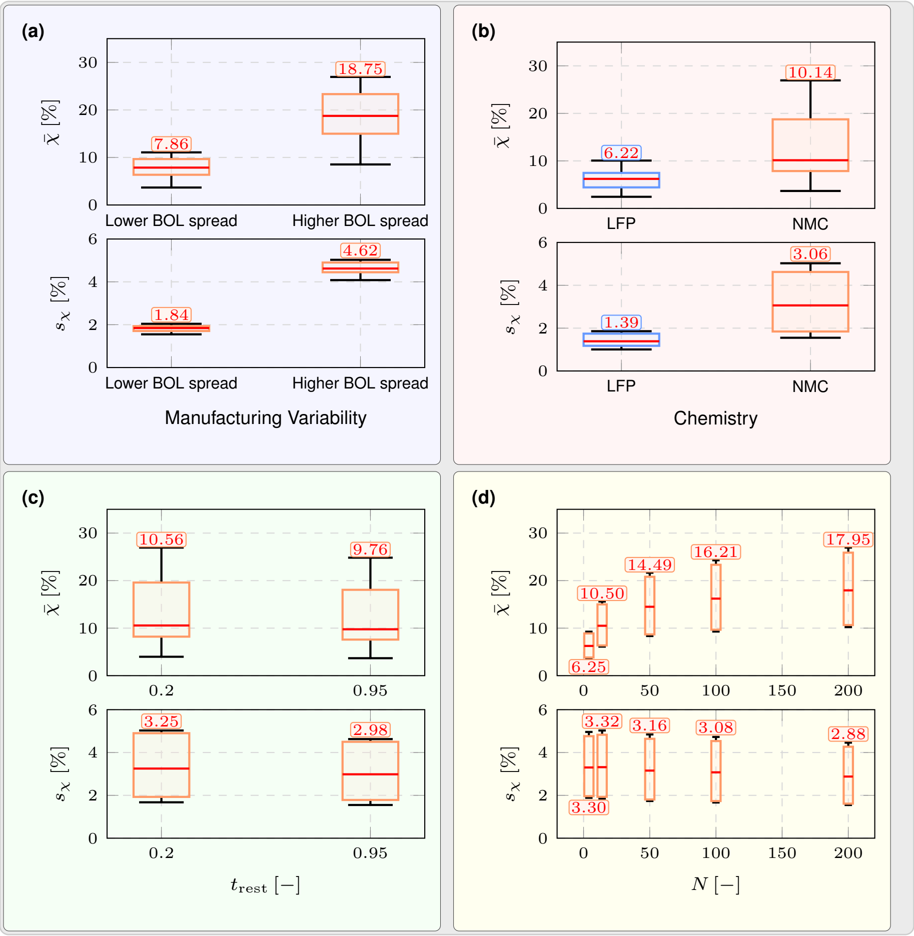

Supplementary Fig. S2 | Sensitivity of lifetime extension of lithium nickel manganese cobalt oxide (NMC) battery packs to key system and environmental factors. Each subfigure contains two panels: the top shows mean lifetime extension ($\bar{\chi}$) and the bottom shows its standard deviation (s_χ), evaluated across all relevant scenarios. Median values are indicated above or below each boxplot. **(a)** Cell manufacturing variability in beginning-of-life (BOL) capacity and resistance. **(b)** Cell chemistry comparison under nominal thermal conditions ($\mu_T = 25°\text{C}$, $\sigma_T = 0°\text{C}$), with blue boxes denoting lithium iron phosphate (LFP) and orange boxes denoting NMC cells. **(c)** Rest period duration t_{rest}. **(d)** The number of series-connected cells N. In all boxplots: center line, median; box limits, upper and lower quartiles; whiskers, 1.5× interquartile range; points, outliers. Source data are provided as a Source Data file.

S5. Model Selection for Voltage-Dependent Lifetime Extension Fitting

To describe the relationship between the expected lifetime extension $\bar{\chi}$ and the nominal battery pack voltage $V_{\text{pack}}^{\text{nom}}$, we considered four candidate models, each reflecting a distinct mathematical relationship between these variables. The first model is a logarithmic fit, denoted as `log`, where

$$\bar{\chi} = a \log(V_{\text{pack}}^{\text{nom}}) + b. \tag{S1}$$

The second model is a square-root fit, referred to as `sqrt`, and defined by

$$\bar{\chi} = a\sqrt{V_{\text{pack}}^{\text{nom}}} + b. \tag{S2}$$

The third model is a power-law fit, labelled as `power`, expressed as

$$\bar{\chi} = a(V_{\text{pack}}^{\text{nom}})^{b}. \tag{S3}$$

The fourth model is a quadratic fit, referred to as `poly2`, given by

$$\bar{\chi} = a(V_{\text{pack}}^{\text{nom}})^{2} + bV_{\text{pack}}^{\text{nom}} + c. \tag{S4}$$

Each model was fitted independently for LFP and NMC chemistries using nonlinear least-squares regression, applied to the median $\bar{\chi}$ values obtained from simulation. Model performance was evaluated using root mean square error (RMSE), mean absolute error (MAE), and the coefficient of determination (R^2).

Among the candidates, the logarithmic model consistently provided the best fit across both chemistries, yielding the lowest RMSE and the highest R^2. The detailed fitting results are reported in Supplementary Table S4.

Supplementary Table S4 | Fitting performance of four voltage-dependent models for predicting lifetime extension $\bar{\chi}$ in LFP and NMC battery systems.

Chemistry	Model	a	b	c	RMSE	MAE	R^2
LFP	`log`	1.541808	1.055415	–	0.3478	0.2683	0.9742
	`sqrt`	0.234192	5.247156	–	0.9050	0.8003	0.8255
	`power`	3.462544	0.180050	–	0.5634	0.4606	0.9324
	`poly2`	-0.000016	0.018950	5.768943	0.8946	0.7535	0.8295
NMC	`log`	4.062631	-1.913175	–	0.3788	0.3450	0.9951
	`sqrt`	0.610135	9.336440	–	1.8393	1.6477	0.8840
	`power`	5.708044	0.225845	–	1.0966	0.9589	0.9588
	`poly2`	-0.000039	0.048621	10.739876	1.6880	1.4885	0.9023

S6. Economic Analysis: Model Parameters for Baseline-Case, Best-Case, and Worst-Case Scenarios

To assess the economic viability of the reconfigurable battery packs (RBPs) compared to conventional battery packs (CBPs), in Fig. 4b– 4d of the main text, we investigate three scenarios: baseline, best-case, and worst-case. The key model parameters used for the economic analysis in each scenario are summarised in Supplementary Table S5.

S7. Economic Analysis: Supplementary Results

In addition to the three most influential factors discussed in the Economic Impact subsection of the main text, namely the nominal battery pack energy ($E_{\text{pack}}^{\text{nom}}$), the annual driving distance (L), and the RBP upfront cost factor (ν), the global sensitivity analysis (Fig. 5a) also includes several other parameters that exhibit weaker correlations with the lifetime cost benefit of RBPs (ΔNPC). These supplementary sensitivities are presented in Supplementary Fig. S3.

Supplementary Fig. S3a indicates that additional ohmic losses from RBP power electronics, denoted by δ_{loss}, exhibit a modest negative correlation with ΔNPC ($\rho = -0.34$; Fig. 5a), as anticipated: Higher δ_{loss} increases energy

Supplementary Table S5 | Model parameters used in the economic analysis across baseline, best-case, and worst-case scenarios.

Parameter	Symbol	Baseline Case	Best Case	Worst Case	Unit
Nominal battery pack energy	$E_{\text{pack}}^{\text{nom}}$	80	120	20	kWh
Cell chemistry type	–	LFP	NMC	LFP	–
EV lifetime	Y_{EV}	18.80	19.51	19.57	years
Lifetime of CBP	Y_{CBP}	10	10	10	years
Lifetime of RBP	Y_{RBP}	11.14	12.52	11.13	years
Discount rate	r	3	2.10	2.01	%
O&M cost rate for CBP	α_{CBP}	2.00	2.78	1.07	%
O&M cost rate for RBP	α_{RBP}	1.00	1.39	0.54	%
Ohmic losses increase factor for RBP	δ_{loss}	3.00	1.00	4.96	%
Annual driving distance	L	12,000	11,429	68,926	km/year
RBP upfront cost factor	ν	7.50	3.00	1.77	%

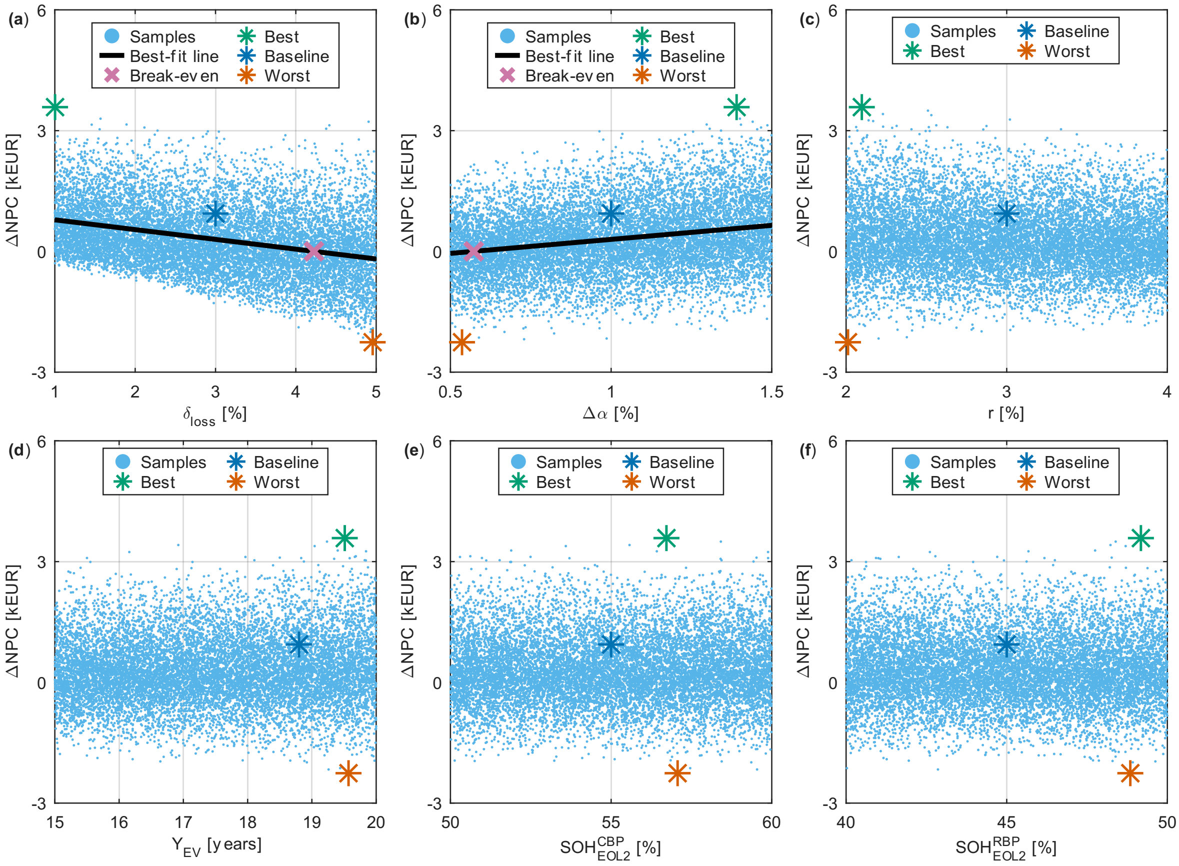

Supplementary Fig. S3 | Additional sensitivity analysis of the lifetime cost benefit of reconfigurable battery packs (RBPs) (ΔNPC) to economic, design, and usage parameters. NPC, net present cost; CBP, conventional battery pack. A positive ΔNPC indicates lifetime cost savings for RBPs relative to CBPs. Each point represents a simulation sample. All subfigures are based on the global parameter sweep described in the Economic Cost Modelling subsection of the Methods section of the main text. **(a–f)** One-dimensional sensitivities of ΔNPC to: additional ohmic losses (δ_{loss}); operational and maintenance (O&M) cost rate difference ($\Delta\alpha = \alpha_{\text{CBP}} - \alpha_{\text{RBP}}$); discount rate ($r$); electric vehicle (EV) lifetime (Y_{EV}); state of health (SOH) at the end of final life for CBP ($\text{SOH}_{\text{EOL2}}^{\text{CBP}}$); and SOH at the end of final life for RBP ($\text{SOH}_{\text{EOL2}}^{\text{RBP}}$). Source data are provided as a Source Data file.

consumption and thus operational costs. Regression analysis of ΔNPC against δ_{loss} (Fig. S3a) identifies a cost break-even threshold at $\delta_{\text{loss}} = 4.23\,\%$, above which RBPs become less economically favourable. However, similar to the case of annual driving distance L in Fig. 5c, this relationship shows considerable variability across scenarios. Given that energy losses represent only a minor share of total lifetime costs (Fig. 4a), the overall impact of δ_{loss} on RBP economic viability is limited. Notably, the identified threshold exceeds typical values reported in the literature for additional losses introduced by reconfiguration hardware [1], suggesting that current RBP designs are already within an acceptable efficiency range.

Supplementary Fig. S3b shows a weak positive correlation between ΔNPC and the difference in O&M cost rates, $\Delta\alpha$ ($\rho = 0.15$; Fig. 5a). This suggests that lower relative maintenance costs modestly enhance RBP economic performance. Nevertheless, this relationship is highly scenario-dependent, and O&M costs constitute the smallest portion of total lifetime expenses (Fig. 4a). As a result, while reducing RBP maintenance costs may offer marginal gains, greater economic improvements are likely to come from targeting major cost components, particularly upfront costs.

As shown in Supplementary Fig. S3c, the discount rate r exhibits a negligible correlation with ΔNPC ($\rho = -0.07$; Fig. 5a), with substantial scenario variability. The high spread in outcomes suggests that other parameters have a more dominant role. The weak sensitivity can be explained by the structure of the cost model (see the Economic Cost Modelling subsection of the Methods section of the main text). The dominant cost components are the upfront cost, replacement cost, and residual value. Among these, only the latter two are affected by discounting, while the upfront cost remains unchanged regardless of r. As r increases, the present value of future replacement and residual costs is reduced, but this effect applies similarly to both conventional battery pack (CBP) and RBP configurations. In particular, the typical timing difference in battery replacement is not large enough to create a significant divergence in discounted values. The residual value from the recently replaced battery pack is realised near the vehicle retirement point and is depreciated by discounting in both cases. As a result, variations in r shift the net present costs (NPC) of both systems in parallel, preserving their relative difference.

As shown in Supplementary Fig. S3d, ΔNPC exhibits a negligible correlation with EV lifetime Y_{EV} ($\rho = 0.04$; Fig. 5a), accompanied by considerable variability within the 15–20 year range. Notably, the regression line remains above zero across the entire interval, indicating that RBPs generally offer cost advantages regardless of the assumed deployment horizon. While longer vehicle lifetimes provide greater opportunity to realise the benefits of delayed replacements and slower degradation, the overall influence of Y_{EV} is minimal compared to other factors. This suggests that the cost-effectiveness of dynamic reconfiguration is robust to variations in the EV lifetime.

To evaluate the reliability of cost savings from RBPs, we analyse two-dimensional parameter spaces using a geometric search for robust regions. A robust region is defined as the largest axis-aligned rectangle within the feasible design space in which the success rate, measured as the fraction of simulations with $\Delta\text{NPC} > 0$, exceeds a specified threshold. We report a primary robust region satisfying a stringent success criterion of 99.7% and a secondary region meeting a relaxed 95% threshold. The search is performed on a normalised 101-by-101 parameter grid. The identified regions are plotted in Supplementary Fig. S4 over the sampled parameter space, with individual simulation outcomes marked in blue ($\Delta\text{NPC} > 0$) or red ($\Delta\text{NPC} < 0$).

S8. Economic Analysis: Sensitivity to Electricity Price and Vehicle Energy Consumption

To assess the robustness of the economic conclusions with respect to regional and usage-dependent variation in energy-related parameters, targeted sensitivity analyses are conducted for both electricity price and vehicle energy consumption. Both parameters enter the economic model through the annual energy cost (see Eqs. 13 and 17), where the cost difference between RBP and CBP is proportional to the product $c^{\text{en}} \cdot \varepsilon \cdot \delta_{\text{loss}}$. Consequently, higher values of either c^{en} or ε scale the ohmic loss penalty proportionally and reduce the net economic benefit of RBPs relative to CBPs. Three representative European levels are evaluated for each parameter: 0.10, 0.24 (baseline), and 0.40 €/kWh for electricity

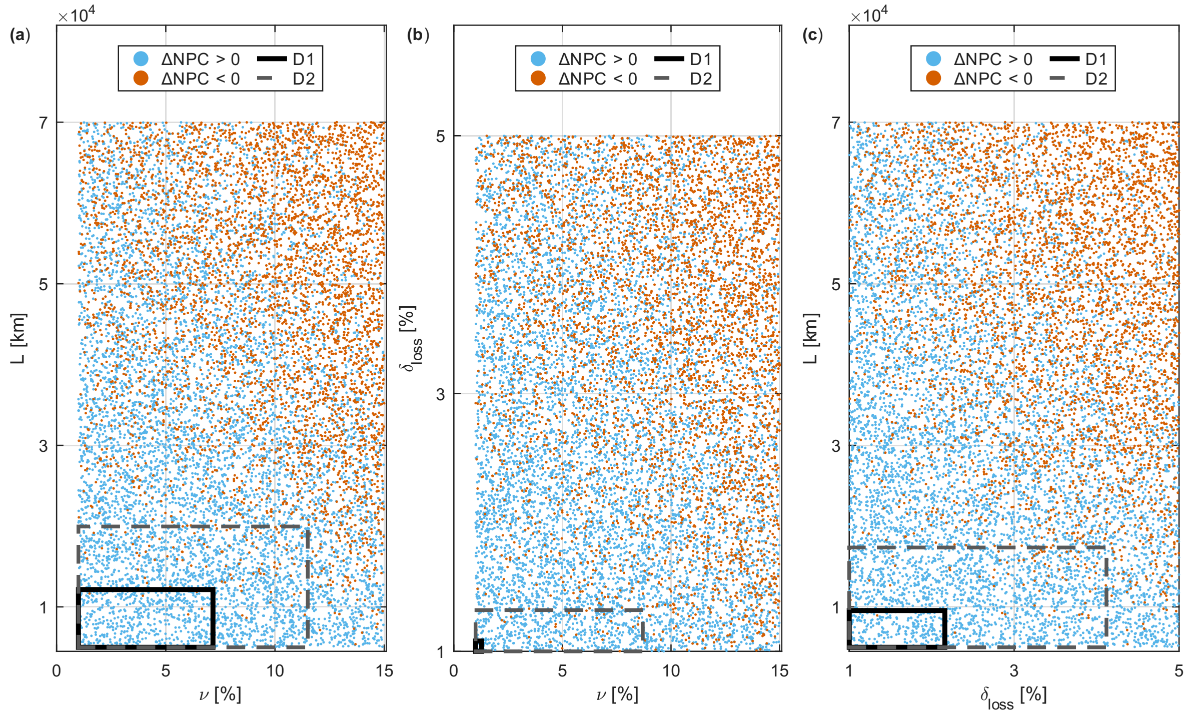

Supplementary Fig. S4 | Robust two-dimensional parameter regions for reconfigurable battery pack (RBP) lifetime cost benefit. NPC, net present cost; ν, RBP upfront cost factor; L, annual driving distance; δ_{loss}, ohmic losses increase factor for the RBP. Blue points indicate individual simulations with ΔNPC > 0; red points indicate ΔNPC < 0. For each parameter pair, the solid black rectangle (**D1**) denotes the primary robust region, defined as the largest axis-aligned area where at least 99.7% of samples yield ΔNPC > 0. The dashed grey rectangle (**D2**) indicates the secondary robust region, where at least 95% of samples yield ΔNPC > 0. **(a)** ν vs. L; **(b)** ν vs. δ_{loss}; **(c)** δ_{loss} vs. L. Source data are provided as a Source Data file.

price, based on recent Eurostat statistics [2]; and 0.15, 0.20 (baseline), and 0.30 kWh/km for vehicle energy consumption, consistent with real-world testing data reporting a range of 0.158 to 0.312 kWh/km across European electric vehicles [3]. For each scenario, 100,000 parameter combinations are generated using Latin Hypercube Sampling, consistent with the main economic analysis. Pack energy is sampled from the same discrete levels as in the main analysis ($\{20, 40, 60, 80, 100, 120\}$ kWh); the large-pack regime is defined as $E_{\text{pack}}^{\text{nom}} \geqslant 50$ kWh, consistent with the break-even threshold identified in the Economic Impact subsection of the main text, with the first discrete level at or above this threshold being 60 kWh. Large packs therefore correspond to $\{60, 80, 100, 120\}$ kWh and small packs to $\{20, 40\}$ kWh. The overall fractions reported below reflect this sampling structure and should be interpreted alongside the pack-stratified results. The results are presented in Supplementary Fig. S5 and summarised in Supplementary Table S6.

The results show that pack size dominance is conditional on operating conditions rather than universal (Supplementary Table S7). Under low and baseline electricity prices and energy consumption, $E_{\text{pack}}^{\text{nom}}$ is the strongest correlate of ΔNPC, with Spearman coefficients of $\rho = 0.739$ and $\rho = 0.671$ at the low scenarios and $\rho = 0.615$ at both baselines. However, at the high electricity price (0.40 €/kWh), annual driving distance L overtakes pack size as the dominant parameter ($\rho = -0.586$ vs. $\rho = 0.483$). The same crossover occurs at high energy consumption (0.30 kWh/km), where L again becomes dominant ($\rho = -0.560$ vs. $\rho = 0.513$). This reflects the underlying mechanism: at high electricity prices or energy consumption, the ohmic loss penalty, which scales with $c^{\text{en}} \cdot \varepsilon \cdot \delta_{\text{loss}} \cdot L$, becomes increasingly sensitive to annual mileage, elevating the influence of L relative to pack size. Notably, δ_{loss} also rises in importance under adverse conditions, entering the top three at both high scenarios ($\rho = -0.439$ at 0.40 €/kWh and $\rho = -0.420$ at 0.30 kWh/km), for the same reason. The parameter ranking under favourable conditions is led by $E_{\text{pack}}^{\text{nom}}$ and ν, whereas under adverse conditions it shifts to L and $E_{\text{pack}}^{\text{nom}}$. This implies that deployment strategy should be conditioned on local

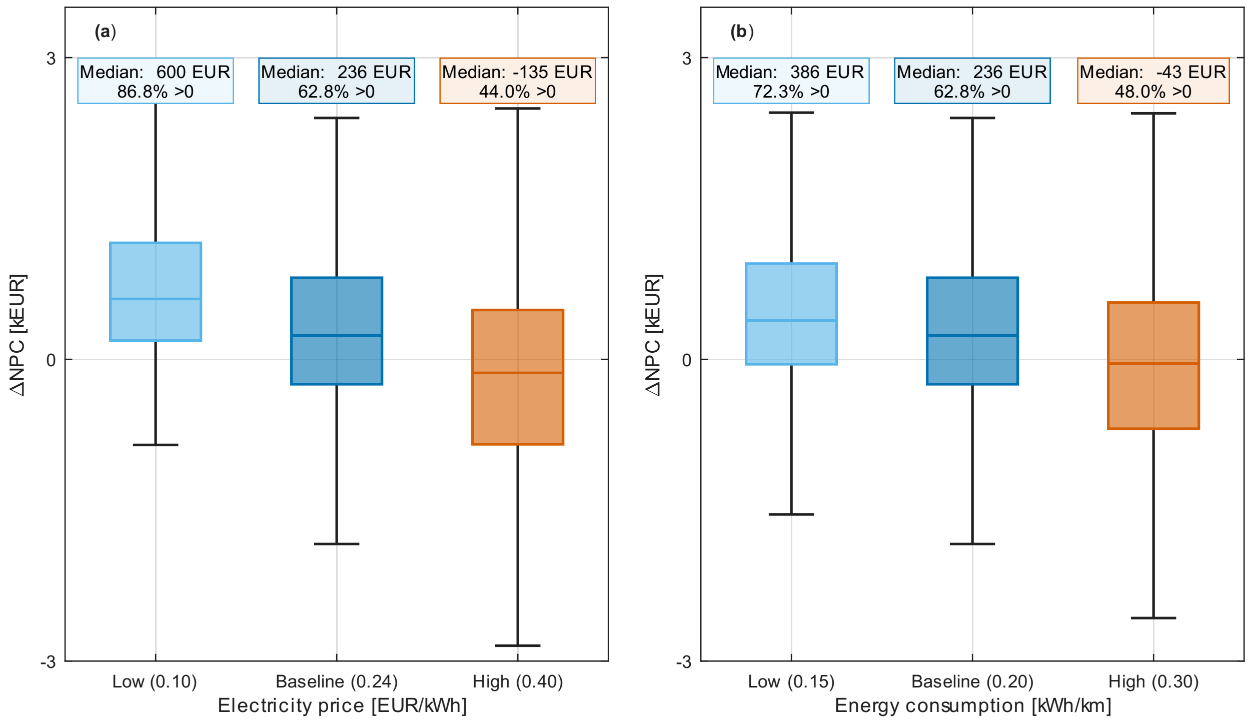

Supplementary Fig. S5 | Sensitivity of the lifetime cost benefit of reconfigurable battery packs (RBPs) (ΔNPC) to electricity price and vehicle energy consumption. NPC, net present cost. Annotated values report the median ΔNPC in € and the fraction of scenarios yielding $\Delta\mathrm{NPC} > 0$. The dashed horizontal line marks $\Delta\mathrm{NPC} = 0$. **(a)** Three representative European electricity price levels: 0.10 €/kWh (lower-cost markets such as Hungary), 0.24 €/kWh (baseline, Sweden [4]), and 0.40 €/kWh (Germany [2]). **(b)** Three representative vehicle energy consumption levels: 0.15 kWh/km (city driving or mild climate), 0.20 kWh/km (baseline, mixed conditions [5]), and 0.30 kWh/km (highway driving or cold-climate winter conditions [3]). All other parameters are varied within the ranges specified in Supplementary Table S12. In all boxplots: center line, median; box limits, upper and lower quartiles; whiskers, 1.5× interquartile range; points, outliers. Source data are provided as a Source Data file.

Supplementary Table S6 | Summary statistics for the electricity price and vehicle energy consumption sensitivity analyses. Median, 25th percentile (P25), and 75th percentile (P75) of ΔNPC are reported in €. Frac>0 denotes the fraction of all 100,000 sampled scenarios yielding $\Delta\mathrm{NPC} > 0$. The final two columns report this fraction restricted to packs of 50 kWh or larger and smaller than 50 kWh, respectively.

**Parameter**	**Scenario**	**Median [€]**	**P25 [€]**	**P75 [€]**	**Frac>0 (all)**	**Frac>0 ($E \geqslant 50$ kWh)**	**Frac>0 ($E < 50$ kWh)**
Electricity price [€/kWh]	Low (0.10)	600	186	1158	86.8%	96.4%	67.4%
	Baseline (0.24)	236	−248	811	62.8%	77.9%	32.8%
	High (0.40)	−135	−845	491	44.0%	57.1%	17.7%
Energy consumption [kWh/km]	Low (0.15)	386	−49	952	72.3%	86.7%	43.6%
	Baseline (0.20)	236	−248	811	62.8%	77.9%	32.8%
	High (0.30)	−43	−691	564	48.0%	61.7%	20.5%

electricity price and driving intensity: in low-price markets, pack size and upfront cost are the key levers for economic viability; in high-price or high-consumption markets, annual mileage and switching losses become equally or more important.

Despite this shift in parameter dominance, pack size remains a strong moderating variable across all scenarios. At the most adverse electricity price (0.40 €/kWh), the overall fraction of favourable scenarios drops to 44.0%, but this aggregate masks a pronounced stratification: large-pack configurations retain 57.1% of favourable outcomes while

Supplementary Table S7 | Spearman rank correlation coefficients between input parameters and ΔNPC across electricity price and energy consumption scenarios. Values are computed from 100,000 Latin Hypercube samples. Bold indicates the dominant parameter per scenario.

	Electricity price [€/kWh]			Energy consumption [kWh/km]		
**Parameter**	Low (0.10)	Baseline (0.24)	High (0.40)	Low (0.15)	Baseline (0.20)	High (0.30)
$E_{\text{pack}}^{\text{nom}}$	**0.739**	**0.615**	0.483	**0.671**	**0.615**	0.513
L	−0.232	−0.449	**−0.586**	−0.370	−0.449	**−0.560**
ν	−0.432	−0.370	−0.298	−0.399	−0.370	−0.314
δ_{loss}	−0.176	−0.340	−0.439	−0.281	−0.340	−0.420
r	−0.108	−0.072	−0.042	−0.087	−0.072	−0.048
α_{CBP}	0.271	0.233	0.188	0.250	0.233	0.198
Y_{EV}	0.086	0.045	0.013	0.061	0.045	0.020

small-pack configurations fall to 17.7%. An analogous pattern holds for energy consumption: at 0.30 kWh/km, the overall fraction is 48.0%, with large packs at 61.7% and small packs at 20.5%. In both cases, the deterioration of economic viability under adverse conditions is therefore concentrated almost entirely in small-pack applications.

Of the two sensitivity parameters, electricity price has the larger effect on ΔNPC. Increasing electricity price from the baseline to 0.40 €/kWh reduces the overall median saving from 236 € to −135 € and the large-pack favourable fraction from 77.9% to 57.1%. Increasing energy consumption from the baseline to 0.30 kWh/km produces a smaller reduction: median saving from 236 € to −43 € and large-pack favourable fraction from 77.9% to 61.7%. This asymmetry is consistent with the Spearman results: at the high electricity price scenario, $|\rho(L)| = 0.586$ compared to $|\rho(L)| = 0.560$ at the high energy consumption scenario, confirming that electricity price amplifies the influence of mileage more strongly than energy consumption does (Supplementary Table S7). Conversely, the low electricity price scenario (0.10 €/kWh) reveals substantial upside: 96.4% of large-pack scenarios are favourable with a median saving of 600 €, more than double the baseline median. This indicates that in lower-price European markets, the economic case for large-pack RBPs is substantially stronger than the baseline analysis suggests.

Taken together, these results complement the main analysis by clarifying how economic outcomes depend on electricity price and vehicle energy consumption. Pack size remains a primary determinant of RBP economic viability, but its dominance is conditional: under high electricity prices or high energy consumption, annual driving distance and switching losses become equally or more influential. Large-pack, low-mileage applications in moderate-price European markets represent the most robust deployment context for RBPs, while high-mileage applications in high-price markets face the most challenging economic conditions regardless of pack size (see Supplementary Table S6).

S9. Sensitivity Analysis: Spearman Rank Correlation Coefficient

We quantify the influence of input parameters on the lifetime cost benefit ΔNPC using the Spearman rank correlation coefficient ρ, a non-parametric measure of monotonic association.

Given a set of N samples from the input space $\{x_i^{(n)}\}_{n=1}^{N}$ and corresponding outputs $\Delta\text{NPC}^{(n)}$, each variable is transformed to its rank:

$$R_{x_i}^{(n)} = \text{rank}\left(x_i^{(n)}\right), \quad R_{\Delta\text{NPC}}^{(n)} = \text{rank}\left(\Delta\text{NPC}^{(n)}\right),$$

where n and i denote the indices of samples and input parameters, respectively.

The Spearman correlation for parameter x_i is computed as:

$$\rho_i = \frac{\sum_{n=1}^{N}\left(R_{x_i}^{(n)} - \overline{R}_{x_i}\right)\left(R_{\Delta\mathrm{NPC}}^{(n)} - \overline{R}_{\Delta\mathrm{NPC}}\right)}{\sqrt{\sum_{n=1}^{N}\left(R_{x_i}^{(n)} - \overline{R}_{x_i}\right)^2}\sqrt{\sum_{n=1}^{N}\left(R_{\Delta\mathrm{NPC}}^{(n)} - \overline{R}_{\Delta\mathrm{NPC}}\right)^2}},$$

where $\overline{R}_{x_i}$ and $\overline{R}_{\Delta\mathrm{NPC}}$ denote the mean ranks. A positive ρ_i indicates that larger values of x_i are associated with larger ΔNPC, whereas a negative ρ_i indicates the opposite. The magnitude $|\rho_i|$ reflects the strength of the monotonic relationship. The method captures both linear and non-linear monotonic relationships and does not rely on distributional assumptions.

S10. Modelling of Battery Cell Behaviour: Electrical Model Parameters

Supplementary Tables S8 and S9 summarise the electrical model parameters for the LFP and NMC cells used in this study. The model structure is consistent across chemistries and includes a series resistance, a single resistor-capacitor (RC) pair, and an OCV-SOC relationship (Supplementary Fig. S6). For the LFP cell, the series resistance and RC pair parameters are temperature-dependent, with measurements provided across multiple temperatures (Supplementary Figs. S7–S9). For the NMC cell, parameter data are available at 25 °C only and are treated as temperature-invariant. The OCV-SOC curves for both chemistries are assumed to be temperature-independent and are provided in Supplementary Fig. S10.

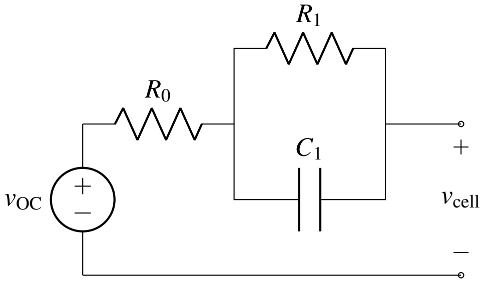

Supplementary Fig. S6 | Equivalent circuit model schematic.

Supplementary Table S8 | Electrical model parameters for the LFP cell.

Parameter	Symbol	Value	Unit	Source
Nominal voltage	V_{cell}^{nom}	3.2	V	[6]
Nominal capacity	Q_{nom}	3.0	Ah	[6]
Minimum terminal voltage (cutoff under load)	V_{cell}^{min}	2.0	V	[6]
Maximum terminal voltage (cutoff under load)	V_{cell}^{max}	3.6	V	[6]
Ohmic resistance	R_0	Fig. S7	mΩ	[6]
Polarization resistance	R_1	Fig. S8	mΩ	[6]
Polarization capacitance	C_1	Fig. S9	kF	[6]
Open-circuit voltage	v_{OC}	Fig. S10	V	[6]

Supplementary Table S9 | Electrical model parameters for the NMC cell.

Parameter	Symbol	Value	Unit	Source
Nominal voltage	V_{cell}^{nom}	3.6	V	[7]
Nominal capacity	Q_{nom}	2.05	Ah	[7]
Minimum terminal voltage (cutoff under load)	V_{cell}^{min}	2.75	V	[7]
Maximum terminal voltage (cutoff under load)	V_{cell}^{max}	4.2	V	[7]
Ohmic resistance	R_0	33.34	mΩ	[7]
Polarization resistance	R_1	11.14	mΩ	[7]
Polarization capacitance	C_1	1.867	kF	[7]
Open-circuit voltage	v_{OC}	Fig. S10	V	[8]

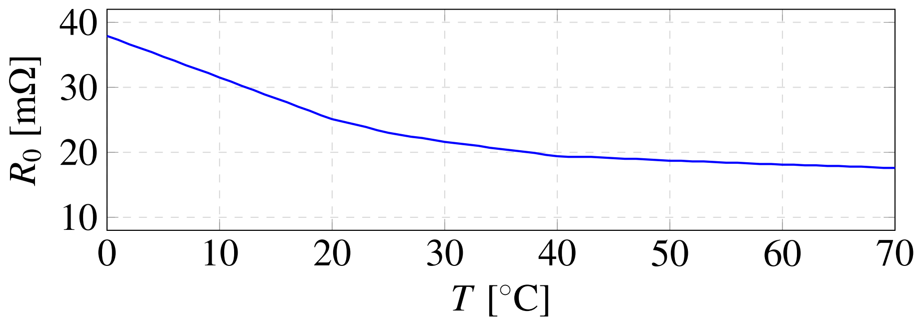

Supplementary Fig. S7 | Temperature dependence of the ohmic resistance R_0 for the lithium iron phosphate (LFP) cell. Data from [6].

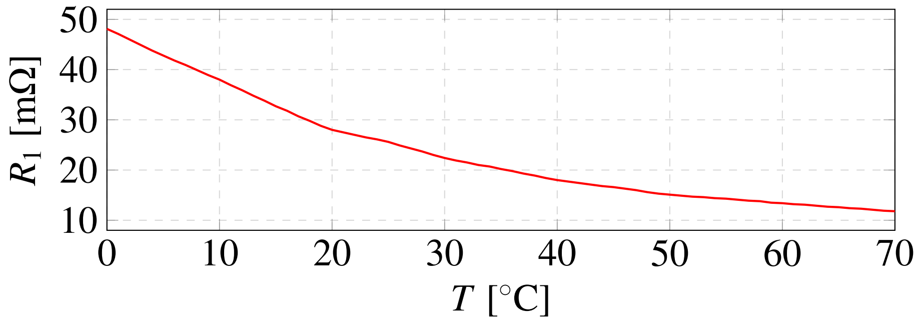

Supplementary Fig. S8 | Temperature dependence of the polarization resistance R_1 for the lithium iron phosphate (LFP) cell. Data from [6].

S11. Cell Manufacturing Variability: Capacity and Resistance of New Lithium-Ion Cells

To capture cell-to-cell variability under beginning-of-life (BOL) conditions, we adopt statistical parameter values: the mean and standard deviation of BOL cell capacity (μ_Q, σ_Q) and of BOL cell resistance (μ_R, σ_R). These values were taken from published studies based on large experimental datasets. Each dataset reports capacity and resistance measurements performed under controlled conditions on new, unused cells. Capacity was determined via constant-current constant-voltage (CC-CV) discharge testing, and resistance was obtained through electrochemical impedance spectroscopy at $\mathrm{Im}(\mathbb{Z}) = 0$. For each chemistry, two distinct parameter sets are considered, each corresponding to a separate test case. The corresponding statistical parameters are summarised in Supplementary Table S10.

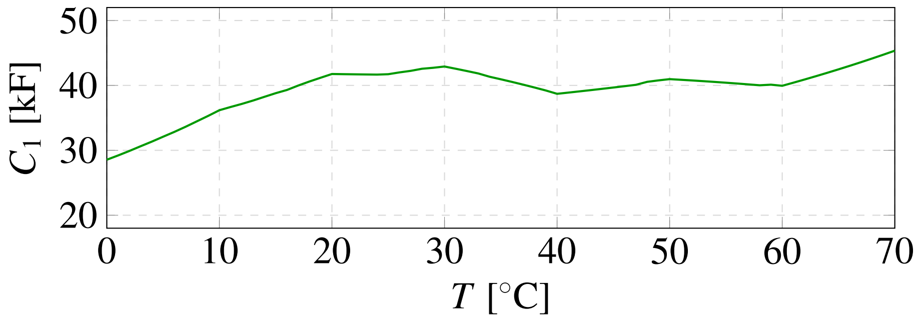

Supplementary Fig. S9 | Temperature dependence of the polarization capacitance C_1 for the lithium iron phosphate (LFP) cell. Data from [6].

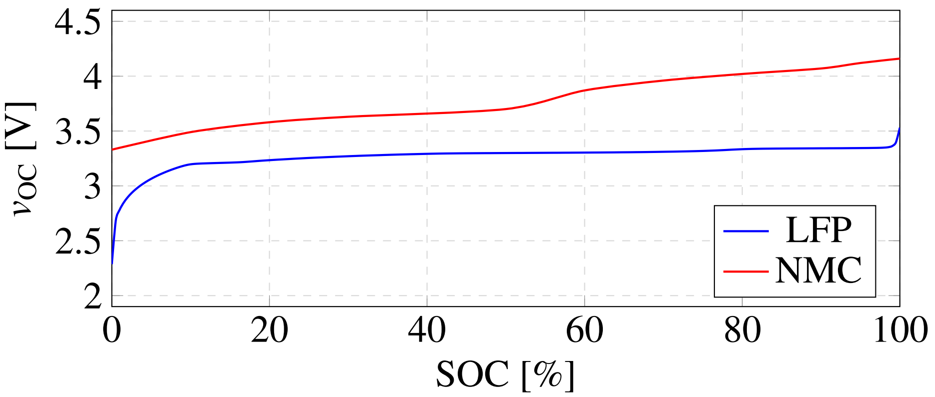

Supplementary Fig. S10 | Open-circuit voltage (OCV) vs. state of charge (SOC) for the lithium iron phosphate (LFP) and lithium nickel manganese cobalt oxide (NMC) cells. The curves show equilibrium voltage under no-load conditions. Minimum terminal-voltage cutoffs under load (Supplementary Tables S8 and S9) are expected to be lower than OCV at the corresponding SOC due to ohmic and polarisation losses. LFP data from [6]; NMC data from [8].

Supplementary Table S10 | Cell-to-cell variability in LFP and NMC battery chemistries based on experimental datasets. CV denotes the coefficient of variation, defined as the standard deviation divided by the mean, expressed in percent.

Chemistry	Dataset	Sample size	μ_Q [Ah]	σ_Q [Ah]	CV_Q [%]	μ_R [mΩ]	σ_R [mΩ]	CV_R [%]	Source
LFP	LFP-1	600	3.020	0.007	0.23	17.642	0.321	1.81	[9]
	LFP-2	500	3.018	0.010	0.33	17.729	0.130	0.73	[9]
NMC	NMC-1	160	3.43	–	0.36	28.7	–	1.04	[10]
	NMC-2	484	1.97	–	0.80	71.15	–	1.94	[11]

S12. Economic Analysis Parameterisation: Cost Estimation for RBP Components

We estimate the additional hardware cost of the RBP based on per-component pricing of automotive-grade electronics. Supplementary Table S11 summarises the unit costs and sources for the RBP-specific components. Prices reflect quotes for quantities of 1000 units, obtained on July 21, 2025. All costs are expressed in EUR.

The RBP introduces additional hardware for switching and control, resulting in a higher upfront cost compared to the CBP. These additional costs are modelled hierarchically, starting from the reconfigurable battery unit up to the full

Supplementary Table S11 | Unit costs for RBP components.

Component	Model	Cost [EUR]	Source
MOSFET	Infineon IAUC120N06S5L011ATMA1	1.19	[12]
Gate Driver	Texas Instruments UCC27425DGNR	0.295	[13]
Fuse	Eaton BK-AMG-300	4.41	[14]
PCB Board	JLCPCB	0.29	[15]
Microcontroller	Renesas R7F7016873AFP-C#AA1	7.32	[16]

pack.

Each cell in the RBP is contained within a reconfigurable battery unit (see Fig. 1), which comprises the cell and the switching circuitry integrated on a printed circuit board (PCB). The circuitry includes two MOSFETs and their corresponding gate drivers. The MOSFET pair enables dynamic reconfiguration: a series MOSFET, placed in the main current path, actively engages the cell, while a bypass MOSFET, connected in parallel to the cell, ensures continuity of current flow when the cell is disengaged. Because MOSFETs cannot be driven directly by low-power logic signals from a microcontroller, each is interfaced through a dedicated gate driver that provides the required voltage and current for reliable switching. The cost of the PCB is denoted as C_{PCB}, the cost of a single MOSFET as C_{MOSFET}, and the cost of a single gate driver as C_{GD}. The additional hardware cost introduced by the RBP functionality per unit is therefore:

$$C_{\mathrm{RBP,Unit}} = C_{\mathrm{PCB}} + 2\left(C_{\mathrm{MOSFET}} + C_{\mathrm{GD}}\right). \tag{S5}$$

Each module in the RBP contains $N_{\mathrm{RBP,Unit}}$ reconfigurable battery units. In addition, each module incorporates an extra pair of MOSFETs with their corresponding gate drivers to enable module-level reconfiguration, for example to bypass the entire module in the event of a fault. Together with a microcontroller for monitoring and control and a fuse for overcurrent protection, these components are integrated on a dedicated module-level PCB. The cost of the microcontroller is denoted as C_{MC}, the cost of the fuse as C_{Fuse}. The additional hardware cost introduced by the RBP functionality per module is therefore:

$$C_{\mathrm{RBP,Module}} = N_{\mathrm{RBP,Unit}} \cdot C_{\mathrm{RBP,Unit}} + C_{\mathrm{PCB}} + 2\left(C_{\mathrm{MOSFET}} + C_{\mathrm{GD}}\right) + C_{\mathrm{MC}} + C_{\mathrm{Fuse}}. \tag{S6}$$

At the pack level, the total RBP-specific hardware cost accounts for all $N_{\mathrm{RBP,Module}}$ modules. In addition, the pack incorporates an extra pair of MOSFETs with their corresponding gate drivers to enable pack-level reconfiguration or isolation, for example to disconnect the entire pack in the event of critical faults or during maintenance. Together with a central microcontroller for overall system management and a high-current fuse for pack-level overcurrent protection, these components are integrated on a dedicated pack-level PCB. The additional hardware cost introduced by the RBP functionality per pack is therefore:

$$C_{\mathrm{RBP,Pack}} = N_{\mathrm{RBP,Module}} \cdot C_{\mathrm{RBP,Module}} + C_{\mathrm{PCB}} + 2\left(C_{\mathrm{MOSFET}} + C_{\mathrm{GD}}\right) + C_{\mathrm{MC}} + C_{\mathrm{Fuse}}. \tag{S7}$$

For a battery pack with nominal energy $E_{\mathrm{pack}}^{\mathrm{nom}} = 60\,\mathrm{kWh}$, nominal voltage $V_{\mathrm{pack}}^{\mathrm{nom}} = 400\,\mathrm{V}$, and module nominal voltage $V_{\mathrm{module}}^{\mathrm{nom}} = 50\,\mathrm{V}$, typical design parameters are assumed: nominal cell voltage $V_{\mathrm{cell}}^{\mathrm{nom}} = 3.2\,\mathrm{V}$ for LFP or $V_{\mathrm{cell}}^{\mathrm{nom}} = 3.6\,\mathrm{V}$ for NMC.Under these assumptions, the additional hardware cost introduced by the RBP increases the CBP upfront cost by approximately 9.36 % for LFP and 8.47 % for NMC.

S13. Economic Analysis Parameterisation: Parameter Ranges

The complete set of parameter ranges explored in the economic analysis is summarised in Supplementary Table S12. Each parameter was varied independently using Latin Hypercube Sampling (LHS).

Supplementary Table S12 | Parameter ranges used in the economic analysis.

Parameter	Symbol	Range	Unit
EV lifetime	Y_{EV}	15 – 20	years
Discount rate	r	2 – 4	%
O&M cost rate for CBP	α_{CBP}	1 – 3	%
O&M cost rate for RBP	α_{RBP}	$0.5\,\alpha_{\mathrm{CBP}}$	%
Ohmic losses increase factor for RBP	δ_{loss}	1 – 5	%
Cell chemistry	–	LFP, NMC	–
Annual driving distance	L	5,000 – 70,000	km/year
RBP upfront cost factor	ν	1 – 15	%
Nominal battery pack energy	$E_{\mathrm{pack}}^{\mathrm{nom}}$	20, 40, . . . , 120	kWh

References

[1] J. Engelhardt, J. M. Zepter, T. Gabderakhmanova, M. Marinelli, Efficiency characteristic of a high-power reconfigurable battery with series-connected topology, in: Proc. Int. Power Electron. Conf., 2022, pp. 2370–2376.

[2] Eurostat, Electricity price statistics, https://ec.europa.eu/eurostat/statistics-explained/index.php?title=Electricity_price_statistics, accessed: 2026-03-02 (2025).

[3] Green NCAP, Estimated real-world energy consumption and driving range: Test procedure, version 1.0.0, https://www.greenncap.com/wp-content/uploads/GNT_Estimated-real-world-energy-consumption-and-driving-range_Test-Procedure_v1.0.0.pdf, accessed: 2026-03-02 (2025).

[4] Qery AS, Consumer electricity prices for households in Europe, https://qery.no/consumer-energy-prices-in-europe/, accessed: 2025-07-15 (2025).

[5] EV Database, Energy consumption of full electric vehicles cheatsheet, https://ev-database.org/cheatsheet/energy-consumption-electric-car, accessed: 2025-07-01 (2025).

[6] M. Schimpe, M. Naumann, N. Truong, H. C. Hesse, S. Santhanagopalan, A. Saxon, A. Jossen, Energy efficiency evaluation of a stationary lithium-ion battery container storage system via electro-thermal modeling and detailed component analysis, Appl. Energy 210 (2018) 211–229.

[7] J. Engels, B. Claessens, G. Deconinck, Techno-economic analysis and optimal control of battery storage for frequency control services, applied to the German market, Appl. Energy 242 (2019) 1036–1049.

[8] S. Käbitz, Untersuchung der Alterung von Lithium-Ionen-Batterien mittels Elektroanalytik und elektrochemischer Impedanzspektroskopie, Tech. rep., Inst. Stromrichtertech. Elektr. Antriebe (2016).

[9] K. Rumpf, M. Naumann, A. Jossen, Experimental investigation of parametric cell-to-cell variation and correlation based on 1100 commercial lithium-ion cells, J. Energy Storage 14 (2017) 224–243.

[10] M. Schindler, J. Sturm, S. Ludwig, J. Schmitt, A. Jossen, Evolution of initial cell-to-cell variations during a three-year production cycle, eTransportation 8 (2021) 100102.

[11] S. F. Schuster, M. J. Brand, P. Berg, M. Gleissenberger, A. Jossen, Lithium-ion cell-to-cell variation during battery electric vehicle operation, J. Power Sources 297 (2015) 242–251.

[12] Mouser Electronics, Infineon Technologies IAUC120N06S5L011ATMA1, https://eu.mouser.com/ProductDetail/Infineon-Technologies/IAUC120N06S5L011ATMA1?qs=8W1m6%252BaMh8TWVMx7jEvedQ%3D%3D, accessed: July 21, 2025 (2025).

[13] Mouser Electronics, Texas Instruments UCC27425DGNR, https://eu.mouser.com/ProductDetail/Texas-Instruments/UCC27425DGNR?qs=eJkN62t4xTW7fovPAhgefQ%3D%3D, accessed: July 21, 2025 (2025).

[14] Mouser Electronics, Bussmann / Eaton BK-AMG-300, https://eu.mouser.com/ProductDetail/Bussmann-Eaton/BK-AMG-300?qs=sGAEpiMZZMsIz3CjQ1xegREPq50MdNHEJ%252BTMnHagrwM%3D, accessed: July 21, 2025 (2025).

[15] JLCPCB, Instant PCB quote – JLCPCB, https://cart.jlcpcb.com/quote?spm=Jlcpcb.Homepage.1006, accessed: July 21, 2025 (2025).

[16] Mouser Electronics, Renesas R7F7016873AFP-C#AA1, https://eu.mouser.com/ProductDetail/Renesas-Electronics/R7F7016873AFP-CAA1?qs=81r%252BiQLm7BQyAhnPzQ5HyA%3D%3D, accessed: July 21, 2025 (2025).
