# Supplementary material for: System-level assessment of dynamic reconfiguration for lifetime and cost outcomes in electric vehicle battery packs
Source: Nat Commun. 2026 Jul 8;17:5980. doi: 10.1038/s41467-026-74951-8 (PMC13346987; doi:10.1038/s41467-026-74951-8)
Supplement: Supplementary file 2 — Transparent Peer Review file [file 41467_2026_74951_MOESM2_ESM.pdf]

nature portfolio

Peer Review File

System-Level Assessment of Dynamic Reconfiguration for Lifetime and Cost Outcomes in Electric Vehicle Battery Packs

Corresponding Author: Professor Changfu Zou

This file contains all reviewer reports in order by version, followed by all author rebuttals in order by version.

Version 0:

Reviewer comments:

Reviewer #1

(Remarks to the Author)
This manuscript presents a technology-agnostic evaluation of dynamic reconfiguration in electric vehicle (EV) battery packs. Using detailed cell-level electrochemical models, ageing models, Monte Carlo simulations (1000 per scenario), and large design-space exploration across manufacturing variability, thermal heterogeneity, usage patterns, system voltages, and chemistries, the authors quantify lifetime extension benefits and translate them into a rigorous techno-economic assessment. Notably, the study identifies ≥20% lifetime gains (even higher for high-voltage packs), derives economic break-even thresholds (≈50 kWh pack size, <7% additional upfront cost), and highlights when and why reconfigurable battery packs (RBPs) outperform conventional fixed packs (CBPs) in both technical and economic terms. The methodology is systematic, the contributions are clearly novel, and the findings have broad implications for next-generation EV design, online management, and retirement management.

In general, the reviewer believes this is a strong and well-executed manuscript that provides one of the most rigorous evaluations to date of dynamic reconfiguration impacts at both the cell and system levels. The integration of ageing-aware cell modelling, statistical variability, and full-lifecycle economic analysis is a substantial contribution, and directly addresses long-standing questions about the viability of RBPs in real-world EV platforms. The work is timely, highly relevant to the EV, BMS, and battery sustainability communities, and has the potential to influence both industrial design choices and academic research directions. Thus, the reviewer believes the work to be highly suitable for publication in Nature Communications.

Major Comments:
1.In the Introduction, it is recommended to supplement the description of, if any, existing methods for evaluating the lifetime and economics of reconfigurable batteries.
2.The analysis assumes full cell-level reconfiguration (i.e., every cell can be bypassed or engaged independently) where this represents an upper performance bound, whereas most industrially proposed RBPs adopt cluster-level or string-level reconfiguration due to cost and hardware complexity. The reviewer would suggest the authors comment on these scenarios and their implications, so that the results could be more impactful and industry relevant.
3.The authors use a simulation framework to evaluate 240 scenarios but do not provide specific simulation steps or a detailed workflow. The reviewer would suggest provide the data and code for evaluation and reproducibility check.
4.The economic model is rigorous but contains several assumptions: Linear SOH-to-value mapping (Eq. 26) may not reflect actual second-life market behaviour. Uniform distribution of lifetime gains (Eq. 18) simplifies scenario-specific uncertainty. Ohmic loss penalties (1–5%) may underestimate long-term efficiency impacts in commercial fleets. Residual value calculations assume consistent demand for second-life packs. In this regard, can the authors provide dynamical evaluation of the results by substituting the statical assumptions into the dynamical initial conditions of the second-life batteries? While the reviewer knows that such information can be very challenging to obtain, there are several emerging research can provide such information using millisecond to seconds level data, (Journal of Power Sources 597, 234156, Nature Communications 15 (1), 10154). It would be beneficial for the authors to explicitly discuss these.
5.The paper assumes that reconfiguration operates optimally to maximise lifetime extension. However, RBPs introduce a control policy problem: How does the controller decide which cells to bypass, when, and how often? Could operational strategy deviations reduce the theoretical lifetime extension? The authors may comment on the role of machine learning or predictive control in real deployments.

Minor Comments:
1.In the analysis of Figure 3, "Sensitivity of lifetime extension to key system and environmental factors," since a simulation method is used, are the number of parameters set sufficient? Especially for t_rest, which only has two parameter values. It is recommended that the authors explain the rationale behind the parameter settings in detail in Section 2.
2.The manuscript uses a 1RC equivalent circuit model to simulate battery electrical signals and an empirical model to simulate aging. Why were these two relatively simple models chosen? Can they effectively characterize the battery's state? How is the accuracy of the results ensured? The reviewer would suggest the authors review the existing data-driven methods that have enhanced the degradation modeling of the second-life batteiries, and discuss the possibility of using them for an improved analysis.
3.For the NMC battery in Supplementary Table S5, the cutoff voltage is 2.75V, which differs from the OCV-SOC curve shown in Supplementary Fig. S8. The authors should check this carefully and make the presented information consistent. Futhermore, could the OCV plateau region of the LFP battery impact the simulation results?
4.In Section 2 (Results), the fifth page includes parameter definitions for many of the five key influencing factors, interspersed within the text description. It is recommended to reorganize this information or supplement it with a table in the SI.
5.The authors are advised to check the font sizes and colors in all figures throughout the manuscript. For example, some fonts in Figure 1a are too small, and the colors in Figure 4 are similar to the shading, reducing clarity.
6.The authors should carefully check the formatting of mathematical equations, especially the use of upright and italic fonts.

In summary, the paper is exceptionally novel and robust. Pending some minor revisions, and added numerical experiments and the code data deposition, and the expanded discussion of the implication of the paper to a general second-life battery management landscape, this paper would be certain for publication in Nature Commnucations.

(Remarks on code availability)
The reproducibility of the figures is great, and it would be even better to open-source the modeling part (I guess the authors intended to do so when the paper can be considered to proceed with publication).

Reviewer #2

(Remarks to the Author)
I co-reviewed this manuscript with one of the reviewers who provided the listed reports. This is part of the Nature Communications initiative to facilitate training in peer review and to provide appropriate recognition for Early Career Researchers who co-review manuscripts.

(Remarks on code availability)

Reviewer #3

(Remarks to the Author)

The manuscript proposes a systematic evaluation of dynamic reconfiguration technology for EV battery packs. In particular, a simulative approach based on empirical aging model and Monte Carlo simulations is used to assess the capability of dynamic reconfiguration technology to extend battery lifetime and increase residual value.
The analysis investigates the sensitivity of lifetime extension from several factors, i.e. battery chemistry (LFP or NMC), temperature distribution, cell manufacturing variability and battery pack voltage. In addition, the manuscript provide also a basic economic feasibility analysis.
The manuscript is practical and interesting, however it presents some minor issues that should be addressed to improve flow linearity and the overall readability of the manuscript.

1)In particular, a concise description of the method employed (in particular the simulation model used and the workflow) should be included in the introduction to provide the user with the basic information needed to better understand workflow of the study.

2) The second paragraph of subsection 2.1("Manufacturing inconsistencies ... unattainable") provides basic information that would be more appropriately placed at the beginning of the subsection rather than in its current position.

3) It could be useful to enhance manuscript readability, to improve figures clarity and self-consistency. As an example, subplots of Figure 5 could be grouped in two or more different figures to enhance readability.

4)Subfigure (g) of Figure 1 suggests an environmental impact analysis that is not discussed in the current manuscript. For consistency, the authors should either include it in the manuscript or remove the subfigure.

(Remarks on code availability)

Reviewer #4

(Remarks to the Author)
General:
• Interesting and important topic that fits within the scope of Nature Communications.
• However, the written version lacks scientific rigor, which is evident in the written version (compare Section Manuscript Presentation) and in the unclear description of the methodological approach, which raises questions (compare Section Methods). In addition, the interpretations of some results are contradictory (compare Section Results).

Manuscript Presentation:
• Past-Tense in the results and methodology chapters
• Figures are too large, have different font sizes, and descriptions under the figures are too long.
• Most of the subfigures of Fig. 2 don’t have any meaning. I propose replacing Fig. 2 by a short section in the results chapter and a much more condensed figure.
• In Fig. 5a, the colors are indistinguishable when printed out. Additionally, mixing red and green in one plot is bad practice because of red-green weakness.
• I don’t see a large benefit in the analysis of Section 2.6, as all related results can already be derived from the previous sections. Instead, it would be much more beneficial to run additional sensitivity studies on the following matters:
o Full analysis NMC
o More conservative approach regarding energy prices, resale value of degraded RBP’s, and energy consumption
o While reviewing the code, it is now clear why Section 2.6 is necessary. However, it is not well explained in the text. Additionally, relying solely on the battery voltage seems to be counterintuitive
• A discussion chapter dealing with potential flaws of such a system or different solutions (e.g., selling the car with a larger gross battery size) to the problem is completely missing.
• The functionality of such systems is only briefly mentioned on two separate occasions.
• Chapter 4.1 would benefit from more precise citations. It is not clear from which semi-empirical model your model is derived, and what your own contribution is.
• Fig. 1 should solely focus on the principle of RPBs. Fig. 1c and b can be summarized in a table; Fig. 1d can be removed.
• I don’t understand this terminology:
o “while σT took values in {0, 5/12, 5/6}°C”
o “The smallest values (N = 4, 14/16)”
• Apply a legend in Fig. 3f
• There is a typo in Formula 19. Parentheses are set incorrectly; However, the formula is correct in the code. Formula 19 should be like this: c = p1 * exp(-p2*(yinit – y0)) +p3

Methods:
• Assumptions:
o Although the trend is toward cars that last longer, cars currently only last an average of 10 to 11 years. Accidents can, for example, negate the benefits of a system with high upfront costs.
o Usage pattern: Employing the usage pattern of a mining hauler sounds arbitrary. There are probably a lot more scenarios between the private case and the mining hauler
o As I understand, the switching behavior itself of the RBP is not implemented in the code. This should be stated in the limitations/conclusion section.
o It is questionable if the RBP achieves higher resale values than CBP, as in second-life applications, the cells usually need to be disassembled and tested individually. In this case, it does not matter if all “units” that form the battery pack are aged equally. The effort is potentially the same or even higher when the switches need to be disassembled as well. This should be communicated clearly.
o Although 24 ct/kWh is a valid assumption for the majority of Europe, it would be interesting to do a sensitivity study with higher prices, such as in Germany, as vehicle models are usually produced for Europe and not for specific countries.
o Fixed energy consumption of 0.2 kWh/km. The energy consumption is dependent on the user behavior and climate conditions. Some users use vehicles mainly for long-range missions (Autobahn higher consumption) or only in the city (lower consumption). It is colder in Winter in Sweden than in Italy. I suggest a sensitivity analysis.
o As the battery modeling is described in detail, I suggest also presenting the key facts of the modeled cells: capacity in Ah, rated cycles, etc.
• Literature:
o Similar systems are already available in the stationary market (STABL Energy GmbH). How do such systems compare in price and reliability to conventional systems?

Results:
• Contradiction in Use-case results
o “These findings underscore that dynamic reconfiguration is particularly well-suited to high-voltage EV architectures. Long-range BEVs, performance-oriented EVs, and commercial electric trucks, where both system voltage and the number of series-connected cells are high, stand to gain the most.”
o “Notably, we find that for v below approximately 7.16% and annual driving distances under 12,150 km, RBPs deliver cost benefits across nearly all tested scenarios.”
o Electric trucks usually drive more than 100.000 km per year, which diminishes the cost efficiency of RBPs. Additionally, given the 100.000 km per year, the lifetime of Trucks is much shorter than assumed in this publication (ca. 12 years). Although each result might be correct, the findings should be presented clearly and put into context. Since battery packs in

trucks are more accessible than in passenger cars, replacing the battery may be a more cost-effective option.
• When battery packs are bypassed, what effect does it have on the vehicle's range? From a user's perspective, it does not make a difference whether the battery size is reduced due to “balanced aging” or the aging effect.

Summary
The manuscript presents an interesting and important topic that fits within the scope of Nature Communications. However, there are significant issues with the manuscript's scientific rigor, including the use of past tense in the results and methodology chapters, inconsistencies in figure presentation, and unclear methodological descriptions. The results also contain contradictions, and there is a lack of discussion on potential flaws or alternative solutions to the problem.
The assumptions made in the methods section are questionable, and the literature review could benefit from a comparison of similar systems in the stationary market. The results show contradictions in the use-case results, particularly regarding the cost-efficiency of RBPs in electric trucks. The effect of bypassing battery packs on the vehicle's range is also not addressed.
Overall, the manuscript requires significant revisions to improve its scientific rigor and clarity, including addressing the identified issues in the methods, results, and discussion sections. Additional sensitivity studies and a more thorough literature review are also strongly recommended.

(Remarks on code availability)
For reproducibility and ease of use it would be beneficial to run the simulations and publish the code in Python. I don't see any reason why a Matlab/Simulink package would be better suited than Python in this case.

The code is executable, however, the presented framework itself is not included in the code (only plots and the economic assessment are included). From the presented results, it is clear, that the technology is sensitive to specific conditions. That is why the framework itselft is needed to test specific configurations for a high impact of the publication.

Reviewer #5

(Remarks to the Author)
I co-reviewed this manuscript with one of the reviewers who provided the listed reports. This is part of the Nature Communications initiative to facilitate training in peer review and to provide appropriate recognition for Early Career Researchers who co-review manuscripts.

(Remarks on code availability)

Version 1:

Reviewer comments:

Reviewer #1

(Remarks to the Author)
The reviewers appreciate the authors' careful revision. In our view, the authors have satisfactorily addressed the concerns raised in previous review. The revision has strengthened the manuscript in several key aspects, including prior-work positioning, clarification of cell-level versus cluster-/string-level reconfiguration, simulation workflow and reproducibility, and presentation consistency. Overall, the manuscript is now clearer, more rigorous, and practically relevant. We remain convinced that this work is novel and timely, and we support its acceptance.

(Remarks on code availability)

Reviewer #2

(Remarks to the Author)
I co-reviewed this manuscript with one of the reviewers who provided the listed reports. This is part of the Nature Communications initiative to facilitate training in peer review and to provide appropriate recognition for Early Career Researchers who co-review manuscripts.

(Remarks on code availability)
The code is clean and excutable.

Reviewer #3

(Remarks to the Author)
Authors successfully addressed the issues highlighted in the previous reviewing round. I have no further questions.

(Remarks on code availability)

Reviewer #4

(Remarks to the Author)
Dear Mr. Zou,

Thank you for your careful and thorough revision of the manuscript. I have reviewed your responses and the updated version, and I am satisfied that you have addressed all of my previous comments and questions.

I appreciate the clarity of your revisions and the effort you put into improving the manuscript. I have no further concerns and wish you the best with its publication.

(Remarks on code availability)

Reviewer #5

(Remarks to the Author)
I co-reviewed this manuscript with one of the reviewers who provided the listed reports. This is part of the Nature Communications initiative to facilitate training in peer review and to provide appropriate recognition for Early Career Researchers who co-review manuscripts.

(Remarks on code availability)

Open Access This Peer Review File is licensed under a Creative Commons Attribution 4.0 International License, which permits use, sharing, adaptation, distribution and reproduction in any medium or format, as long as you give appropriate credit to the original author(s) and the source, provide a link to the Creative Commons license, and indicate if changes were made.
In cases where reviewers are anonymous, credit should be given to 'Anonymous Referee' and the source.
The images or other third party material in this Peer Review File are included in the article’s Creative Commons license, unless indicated otherwise in a credit line to the material. If material is not included in the article’s Creative Commons license and your intended use is not permitted by statutory regulation or exceeds the permitted use, you will need to obtain permission directly from the copyright holder.
To view a copy of this license, visit https://creativecommons.org/licenses/by/4.0/

Response to Reviewers

Nature Communications

Manuscript Title: "System-level benefits of dynamic reconfiguration in electric vehicle battery packs: Lifetime extension and economic viability"
Manuscript Number: NCOMMS-25-71405-T

Contents

1 Reviewer #1 Comments 2

2 Reviewer #2 Comments 13

3 Reviewer #3 Comments 14

4 Reviewer #4 Comments 19

5 Reviewer #5 Comments 41

1 Reviewer #1 Comments

General assessment

This manuscript presents a technology-agnostic evaluation of dynamic reconfiguration in electric vehicle (EV) battery packs. Using detailed cell-level electrochemical models, ageing models, Monte Carlo simulations (1000 per scenario), and large design-space exploration across manufacturing variability, thermal heterogeneity, usage patterns, system voltages, and chemistries, the authors quantify lifetime extension benefits and translate them into a rigorous techno-economic assessment. Notably, the study identifies $\geq 20\,\%$ lifetime gains (even higher for high-voltage packs), derives economic break-even thresholds ($\approx 50\,\mathrm{kWh}$ pack size, $< 7\,\%$ additional upfront cost), and highlights when and why reconfigurable battery packs (RBPs) outperform conventional fixed packs (CBPs) in both technical and economic terms. The methodology is systematic, the contributions are clearly novel, and the findings have broad implications for next-generation EV design, online management, and retirement management.

In general, the reviewer believes this is a strong and well-executed manuscript that provides one of the most rigorous evaluations to date of dynamic reconfiguration impacts at both the cell and system levels. The integration of ageing-aware cell modelling, statistical variability, and full-lifecycle economic analysis is a substantial contribution, and directly addresses long-standing questions about the viability of RBPs in real-world EV platforms. The work is timely, highly relevant to the EV, BMS, and battery sustainability communities, and has the potential to influence both industrial design choices and academic research directions. Thus, the reviewer believes the work to be highly suitable for publication in Nature Communications.

Author response: We thank the reviewer for the encouraging overall evaluation and for highlighting the contribution of integrating ageing-aware cell modelling, statistical variability, and techno-economic analysis in a unified framework. We have addressed all major and minor comments and revised the manuscript accordingly, with particular emphasis on clarifying the literature context, practical implementability (granularity), and reproducibility (workflow and code availability). We believe these revisions have strengthened both the technical rigor and the industry relevance of the work.

Major comments (MaC)

MaC-Q1: In the Introduction, it is recommended to supplement the description of, if any, existing methods for evaluating the lifetime and economics of reconfigurable batteries.

Author response: We thank the reviewer for this valuable suggestion. The Introduction has been revised to more clearly position the present work with respect to existing studies on reconfigurable battery systems. Specifically, we now explicitly clarify that prior work has primarily focused on immediate functional benefits or case-specific techno-economic analyses, without providing systematic, generalisable evaluations across chemistries, operating, and design conditions. This clarification is reflected in the second paragraph of the revised Introduction. For reviewer's convenience, we reproduce the relevant text below:

· · · .Yet, existing studies have primarily focused on immediate advantages such as active balancing or fault diagnosis, treating lifetime extension and cost savings as secondary benefits [20, 21]. Furthermore, the available techno-economic assessments tend to be case-specific [22] or architecture-limited [23], lacking systematic evaluations that generalise across diverse degradation scenarios, environmental conditions, and usage profiles.

MaC-Q2: The analysis assumes full cell-level reconfiguration (i.e., every cell can be bypassed or engaged independently) where this represents an upper performance bound, whereas most industrially proposed RBPs adopt cluster-level or string-level reconfiguration due to cost and hardware complexity. The reviewer would suggest the authors comment on these scenarios and their implications, so that the results could be more impactful and industry relevant.

Author response: We thank the reviewer for this important observation. We agree that full cell-level reconfiguration represents an upper-bound configuration and that industrial research efforts and pre-commercial demonstrator studies also explore coarser reconfiguration granularities, such as cluster- or string-level architectures, to balance cost, complexity, and reliability.

In the revised manuscript, we have clarified that the assumed cell-level reconfiguration represents a theoretical upper bound on achievable lifetime extension and performance gains. We further discuss the implications of reconfiguration granularity, noting that coarser architectures manage heterogeneity at an aggregated level and may capture a reduced fraction of achievable performance gains compared to cell-level implementations.

Importantly, we emphasize that the primary value of the present analysis lies in identifying governing trends across system voltage, manufacturing variability, thermal heterogeneity, usage intensity, and chemistry. These trends remain informative for industrial RBP research and design studies, as they indicate when dynamic reconfiguration is fundamentally worthwhile, while the achievable magnitude depends on the chosen granularity. In Section 3 of the revised manuscript, a dedicated discussion has been added to clarify these aspects and to position the results within an industry-relevant context. This text is presented below for reviewer's convenience:

Section 3: Discussion

. . .

Section 3.1 Reconfiguration Granularity

Full cell-level reconfiguration is considered in the analysis presented in Section 2, whereby each cell can be independently engaged or bypassed (Fig. 1). This configuration represents an upper bound on achievable performance, as it maximises the resolution at which cell-to-cell heterogeneity can be identified and acted upon during operation.

In practice, industrial research and pre-commercial demonstrator studies have pre-

dominantly explored coarser granularities, such as cluster- or string-level reconfiguration, as a compromise between performance gains, hardware complexity, packaging constraints, and system reliability [13, 15, 16, 34]. At these scales, heterogeneity is managed in aggregated form, limiting the ability to isolate localised degradation or transient stress. This trade-off highlights a fundamental dependency between reconfiguration granularity and heterogeneity resolution. As granularity coarsens, the system progressively transitions from fine-grained adaptive control toward averaged management, reducing its capacity to mitigate weak-cell effects and redistribute degradation pathways. Consequently, the achievable lifetime extension and associated economic value are intrinsically linked to the architectural level at which heterogeneity can be resolved.

These considerations suggest that reconfiguration should not be viewed as a binary design choice, but rather as a continuum defined by architectural resolution. Identifying the optimal granularity under realistic cost, packaging, and reliability constraints will therefore be central to practical RBP implementation.

MaC-Q3: The authors use a simulation framework to evaluate 240 scenarios but do not provide specific simulation steps or a detailed workflow. The reviewer would suggest provide the data and code for evaluation and reproducibility check.

Author response: We thank the reviewer for the request regarding code availability and reproducibility. The complete repository used in this study is publicly available at:

`https://github.com/askegro/NCOMMS-25-71405-T_Code`

It is organised into two subfolders: `simulation/` contains the MATLAB simulation framework (cell electrical and ageing models, Monte Carlo sampling logic, and CBP/RBP lifetime evaluation), and `postprocessing/` contains all figure generation and economic analysis scripts together with the raw simulation output data for all 240 scenarios, enabling direct reproduction of all reported figures without rerunning the simulations. The full parameter sweep comprises 240 scenarios with 1,000 Monte Carlo realisations each, executed on the National Academic Infrastructure for Supercomputing in Sweden (NAISS), accessed through Chalmers University of Technology. Individual parameter combinations can be run at reduced scale on a standard workstation; usage instructions for both local and HPC environments are provided in the repository `README.md`.

MaC-Q4: The economic model is rigorous but contains several assumptions: Linear SOH-to-value mapping (Eq. 26) may not reflect actual second-life market behaviour. Uniform distribution of lifetime gains (Eq. 18) simplifies scenario-specific uncertainty. Ohmic loss penalties (1–5%) may underestimate long-term efficiency impacts in commercial fleets. Residual value calculations assume consistent demand for second-life packs. In this regard, can the authors provide dynamical evaluation of the results by substituting the statical assumptions into the dynamical initial conditions of the second-life batteries? While the reviewer knows that such information can be very challenging to obtain, there are several emerging research can provide such information using millisecond to seconds level data, (Journal of Power Sources 597, 234156, Nature Communications 15 (1), 10154). It would be beneficial for the authors to explicitly discuss these.

Author response: We thank the reviewer for this thoughtful and technically well-founded comment. We agree that the economic evaluation relies on simplifying assumptions, particularly regarding second-life valuation, lifetime uncertainty, and efficiency penalties. These assumptions were deliberately adopted to ensure transparency and consistency in a system-level assessment spanning a broad design and usage space.

In the revised manuscript, we have expanded the discussion of these assumptions and their implications. Specifically, we clarify that (i) the linear SOH-to-value mapping represents a conservative first-order approximation commonly adopted in early-stage techno-economic studies; (ii) the uniform distribution of lifetime gains is used to propagate uncertainty without imposing scenario-specific priors that would bias the results toward particular applications; and (iii) the assumed range of ohmic loss penalties reflects current automotive-grade reconfiguration hardware, while acknowledging that duty-cycle-dependent losses in high-utilisation commercial fleets may be higher.

We also acknowledge that second-life battery value depends on the internal condition of individual cells at retirement, which cannot be fully captured by static SOH-based proxies. While emerging diagnostic and data-driven approaches can provide more detailed characterisation of these conditions [1, 2] (i.e., Refs. 35–36 in the revised manuscript), their integration into system-level economic analysis would require additional assumptions on second-life deployment, usage profiles, and market structures. We therefore retain the simplified economic model as a baseline for capturing first-order effects of lifetime extension and deferred replacement. The main qualitative trends identified in this work are not tied to a specific valuation formulation and remain applicable under more detailed second-life modelling approaches.

A dedicated discussion has been added to the revised manuscript (Section 3.2) to clarify these aspects and to position the adopted modelling assumptions within the broader context of second-life battery valuation. The relevant text is shown in the following box:

Section 3: Discussion

...

Section 3.2 Economic Modelling Framework and Assumptions

The economic analysis relies on a set of static, transparent assumptions to enable consistent comparison between conventional and reconfigurable battery pack architectures across a wide design and usage space. These include a linear mapping between remaining state of health (SOH) and residual value, a uniform distribution to represent uncertainty in lifetime extension, and a fixed range of ohmic loss penalties associated with reconfiguration hardware (see details in Section 2). Such assumptions are commonly adopted in system-level techno-economic assessments where the objective is to identify governing trends, break-even thresholds, and design envelopes rather than to predict application-specific market outcomes.

In practice, second-life battery value also depends on the internal condition of individual cells at retirement, which cannot be fully represented by static SOH-based proxies. While emerging diagnostic and data-driven approaches can provide more detailed characterisation of these conditions [35, 36], their integration into

system-level economic analysis would require additional assumptions on second-life deployment, usage profiles, and market structures. Consequently, the adopted economic model serves as a baseline for capturing first-order effects of lifetime extension and deferred replacement, while enabling consistent comparison across diverse system configurations.

[1] S. Tao, R. Ma, Y. Chen, Z. Liang, H. Ji, Z. Han, G. Wei, X. Zhang, G. Zhou, Rapid and sustainable battery health diagnosis for recycling pretreatment using fast pulse test and random forest machine learning, J. Power Sources 597 (2024) 234156.

[2] S. Tao, R. Ma, Z. Zhao, G. Ma, L. Su, H. Chang, Y. Chen, H. Liu, Z. Liang, T. Cao, et al., Generative learning assisted state-of-health estimation for sustainable battery recycling with random retirement conditions, *Nat. Commun.* 15 (1) (2024) 10154.

MaC-Q5: The paper assumes that reconfiguration operates optimally to maximise lifetime extension. However, RBPs introduce a control policy problem: How does the controller decide which cells to bypass, when, and how often? Could operational strategy deviations reduce the theoretical lifetime extension? The authors may comment on the role of machine learning or predictive control in real deployments.

Author response: We thank the reviewer for raising this point. We agree that the results presented in this work correspond to an idealised operating scenario.

In the revised manuscript, we clarify that the reported lifetime for RBPs is obtained under an idealised reconfiguration policy and therefore represents an upper bound on achievable lifetime extension. This clarification has been added in Section 5.2 (see the box below).

Section 5: Method

...

Section 5.2 Statistics of Lifetime Extension

For a CBP, the EOL time is defined as the point at which the first cell reaches 80% of its nominal capacity. In contrast, full reconfiguration in RBPs under an idealised control policy assumed in this work enables cells to be actively managed such that their long-term degradation is balanced and they reach the EOL simultaneously. The resulting lifetime therefore represents an upper bound on achievable lifetime extension. ...

We would like to emphasise that the objective of this study is to quantify the system-level potential of dynamic reconfiguration across a broad design and operating space. The analysis therefore focuses on the impact of structural flexibility and cell-to-cell heterogeneity on lifetime extension, without specifying a particular control strategy.

Minor comments (MiC)

MiC-Q1: In the analysis of Figure 3, "Sensitivity of lifetime extension to key system and environmental factors," since a simulation method is used, are the number of parameters set sufficient? Especially for t_rest, which only has two parameter values. It is recommended that the authors explain the rationale behind the parameter settings in detail in Section 2.

Author response: We thank the reviewer for this careful comment regarding the adequacy and rationale of the parameter discretisation used in the sensitivity analysis of the original Fig. 3 (now Fig. 2). We agree that, for simulation-based studies, it is important to clearly justify both the choice and the number of parameter values explored.

In the revised manuscript, we now state explicitly at the beginning of Section 2 that the study is designed as a trend-focused sensitivity analysis rather than an exhaustive exploration of a continuous parameter space. Discrete parameter levels were therefore chosen to represent distinct, practically relevant operating regimes that span the expected range of real-world conditions (see Supplementary Table S2 for the complete list of factor levels and investigated ranges).

Regarding the usage-pattern factor, the rest-time fraction t_{rest} was discretised into representative low- and high-utilisation regimes (Supplementary Table S2), corresponding to intensive duty cycles (e.g., commercial/industrial operation) and predominantly idle operation (typical private EV usage). This discretisation captures the dominant role of utilisation intensity and rest exposure on lifetime trends while keeping the design space computationally tractable. These clarifications have been added directly to the beginning of Section 2 and to the usage-pattern description in Section 2.

> Together, these factors span practically relevant EV design and operating regimes and are selected based on their relevance to battery pack design [24], correspondence to real-world usage [25], and physical links to dominant degradation mechanisms [26]. The definitions, symbols, investigated values, and scenario counts for these factors are summarised in Supplementary Table S2.

MiC-Q2: The manuscript uses a 1RC equivalent circuit model to simulate battery electrical signals and an empirical model to simulate aging. Why were these two relatively simple models chosen? Can they effectively characterize the battery's state? How is the accuracy of the results ensured? The reviewer would suggest the authors review the existing data-driven methods that have enhanced the degradation modeling of the second-life batteries, and discuss the possibility of using them for an improved analysis.

Author response: We thank the reviewer for this thoughtful comment regarding model selection and fidelity.

The 1RC equivalent circuit model (ECM) and the empirical ageing formulation were deliberately selected to balance physical interpretability, computational efficiency, and scalability in a system-level study spanning a large design and operating space. The objective of this work is not to achieve high-precision cell-level voltage or degradation prediction for a specific cell, but rather to quantify relative lifetime-extension trends and techno-economic impacts of dynamic reconfiguration across diverse operating conditions, pack architectures, and chemistries. For this purpose, low-order ECMs and empiric-

ally grounded ageing models are widely adopted in the literature and have been shown to capture the dominant electrical dynamics and degradation dependencies on current, temperature, depth of discharge, and usage intensity [3, 4].

Importantly, the ECM and ageing-model parameters were not re-identified or re-fitted in this work; instead, parameter values and coefficients were adopted from peer-reviewed experimental studies. Specifically, for LFP cells the OCV and ECM parameters (R_0, R_1, C_1) were taken from [5], while for NMC the OCV was taken from [6] and the remaining ECM parameters from [7]. Likewise, the ageing formulations and their coefficients were adopted from experimental studies for LFP calendar/cycle ageing [8, 9] and for NMC calendar/cycle ageing [10]. Additional model details and parameter tables are provided in Supplementary Information S11.

With respect to state characterization, the chosen models represent the battery states required for this study at the pack level (terminal voltage, current distribution, SOC evolution, and aggregate capacity fade and resistance growth). They are not intended to resolve detailed electrochemical states or local degradation mechanisms, which would require higher-fidelity physics-based models and substantially greater parametrisation effort.

The robustness of the reported conclusions is supported by (i) reliance on experimentally grounded parameterisations from the cited sources, (ii) evaluation of a large scenario ensemble across a structured design space, and (iii) Monte Carlo sampling (1,000 runs per scenario) so that results reflect statistically stable comparative trends rather than single realisations. Moreover, the main conclusions are based on relative metrics (mean lifetime extension $\bar{\chi}$, variability s_χ, and cost break-even thresholds) rather than absolute lifetime prediction, which reduces sensitivity to model order.

We acknowledge that recent data-driven and hybrid approaches, including machine-learning-based degradation and SOH models, have demonstrated improved accuracy for cell-level lifetime prediction, particularly for second-life batteries with heterogeneous and previously unknown usage histories [1, 2, 11, 12]. Such methods are highly promising for diagnostics and application-specific forecasting. However, integrating such approaches into a large-scale, technology-agnostic system study would require extensive chemistry- and use-case-specific training datasets and would reduce model transparency and generality. We therefore position the present modelling framework as a conservative and transparent baseline that complements, rather than replaces, higher-fidelity data-driven degradation models.

[3] G. L. Plett, Extended Kalman filtering for battery management systems of LiPB-based HEV battery packs: Part 1. background, J. Power Sources 134 (2) (2004) 252–261.

[4] X. Hu, S. Li, H. Peng, A comparative study of equivalent circuit models for li-ion batteries, J. Power Sources 198 (2012) 359–367.

[5] M. Schimpe, M. Naumann, N. Truong, H. C. Hesse, S. Santhanagopalan, A. Saxon, A. Jossen, Energy efficiency evaluation of a stationary lithium-ion battery container storage system via electro-thermal modeling and detailed component analysis, Appl. Energy 210 (2018) 211–229.

[6] S. Käbitz, J. Tübke, D. U. Sauer, Untersuchung der Alterung von Lithium-Ionen-Batterien mittels Elektroanalytik und elektrochemischer Impedanzspektroskopie, Tech.

rep., Inst. Stromrichtertech. Elektr. Antriebe (2016).

[7] J. Engels, B. Claessens, G. Deconinck, Techno-economic analysis and optimal control of battery storage for frequency control services, applied to the German market, Appl. Energy 242 (2019) 1036–1049.

[8] M. Naumann, M. Schimpe, P. Keil, H. C. Hesse, A. Jossen, Analysis and modeling of calendar aging of a commercial LiFePO4/graphite cell, J. Energy Storage 17 (2018) 153–169.

[9] M. Naumann, F. B. Spingler, A. Jossen, Analysis and modeling of cycle aging of a commercial LiFePO4/graphite cell, J. Power Sources 451 (2020) 227666.

[10] J. Schmalstieg, S. Käbitz, M. Ecker, D. U. Sauer, A holistic aging model for Li(NiMnCo)O2 based 18650 lithium-ion batteries, J. Power Sources 257 (2014) 325– 334.

[11] K. A. Severson, P. M. Attia, N. Jin, N. Perkins, B. Jiang, Z. Yang, M. H. Chen, M. Aykol, P. K. Herring, D. Fraggedakis, et al., Data-driven prediction of battery cycle life before capacity degradation, Nat. Energy 4 (5) (2019) 383–391. 31

[12] P. M. Attia, A. Grover, N. Jin, K. A. Severson, T. M. Markov, Y.-H. Liao, M. H. Chen, B. Cheong, N. Perkins, Z. Yang, et al., Closed-loop optimization of fast-charging protocols for batteries with machine learning, Nature 578 (7795) (2020) 397–402.

MiC-Q3: For the NMC battery in Supplementary Table S5, the cutoff voltage is 2.75 V, which differs from the OCV–SOC curve shown in Supplementary Fig. S8. The authors should check this carefully and make the presented information consistent. Furthermore, could the OCV plateau region of the LFP battery impact the simulation results?

Author response: We thank the reviewer for pointing out this ambiguity and for raising the relevant question regarding the influence of the LFP OCV plateau.

For the NMC cell, the value $V_{\text{cell}}^{\text{min}} = 2.75\,\text{V}$ (Supplementary Table S9) is the minimum terminal voltage cutoff under load specified by the manufacturer/datasheet, whereas Supplementary Fig. S10 reports the open-circuit voltage $v_{\text{OC}}(SOC)$ measured under no-load (equilibrium) conditions. These quantities are not expected to coincide because terminal voltage during discharge includes ohmic and polarisation losses ($v_{\text{cell}} = v_{\text{OC}} - IR_0 - \eta$). We have clarified this distinction in the Supplementary Information by revising the table terminology and expanding the OCV–SOC figure caption.

Regarding the LFP OCV plateau, we agree it can reduce voltage-based SOC observability in practical systems. In our simulations, however, SOC is tracked directly as a model state (not inferred from voltage), and end of life is defined by capacity-based criteria. Therefore, the LFP plateau does not materially affect the simulated degradation trajectories or the reported lifetime-extension metrics. This point has also been clarified in the revised Supplementary Information S10 and S11. Specifically, the relevant entries in Supplementary Tables S9 and S10 have now been updated as:

> Minimum terminal voltage (cutoff under load)
> Maximum terminal voltage (cutoff under load)

The caption of Supplementary Fig. S10 has been revised as follows:

Supplementary Fig. S10 | Open-circuit voltage vs. SOC for the LFP and the NMC cell. The curves show equilibrium voltage under no-load conditions. Minimum terminal-voltage cutoffs under load (Supplementary Tables S8 and S9) are expected to be lower than OCV at the corresponding SOC due to ohmic and polarisation losses.

MiC-Q4: In Section 2 (Results), the fifth page includes parameter definitions for many of the five key influencing factors, interspersed within the text description. It is recommended to reorganize this information or supplement it with a table in the SI.

Author response: We thank the reviewer for this constructive comment. We agree that embedding detailed parameter definitions directly in the Results text reduced readability and interrupted the narrative flow.

In the revised manuscript, Section 2 was streamlined so that it now focuses on the conceptual interpretation of each influencing factor, while detailed definitions, symbols, investigated ranges, and scenario counts have been consolidated into a dedicated summary table in the Supplementary Information. A cross-reference to this table was also added at the beginning of Section 2, as in the box below:

The definitions, symbols, investigated values, and scenario counts for all factors are summarised in Supplementary Table S2.

In addition, representative passages in Section 2 have been revised accordingly, replacing parameter-heavy definitions with shorter conceptual descriptions and direct references to the Supplementary Information. Correspondingly, a new summary table, i.e., Supplementary Table S2, was added in the updated Supplementary Information to consolidate the five key influencing factors, including their physical meaning, parameters/symbols, investigated values/ranges, and the number of scenarios considered.

MiC-Q5: The authors are advised to check the font sizes and colors in all figures throughout the manuscript. For example, some fonts in Figure 1a are too small, and the colors in Figure 4 are similar to the shading, reducing clarity.

Author response: We thank the reviewer for this helpful observation. We agree that in the original manuscript, some figures contained font sizes and color choices that could reduce readability, particularly when viewed in print or at reduced scale.

In the revised manuscript, the colour scheme across all figures has been revised to use a colour-blind-safe palette following the recommendations in [13]. For reviewer's convenience, the updated Fig. 1 and Fig. 4 are provided below:

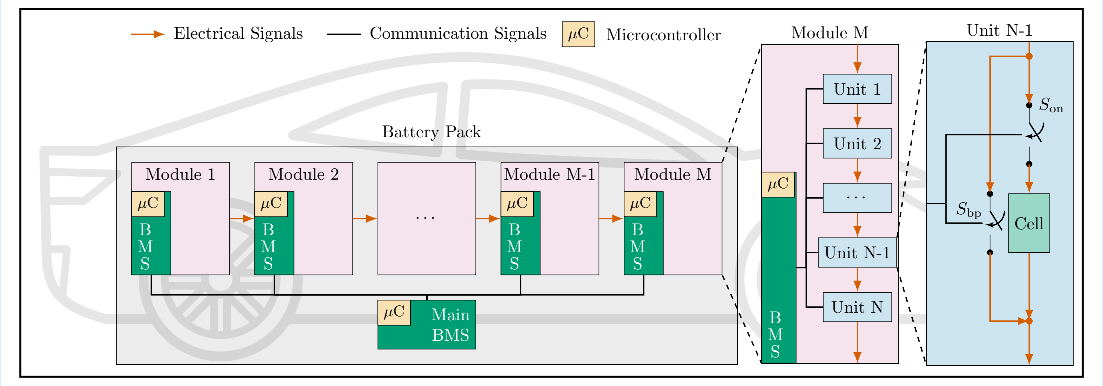

Fig. 1 | Physical architecture of a dynamically reconfigurable battery pack (RBP). Each reconfigurable battery unit consists of a cell and two controllable switches, enabling independent engagement or bypassing during operation. A detailed description of the hierarchical RBP design is provided in Supplementary Information S12.

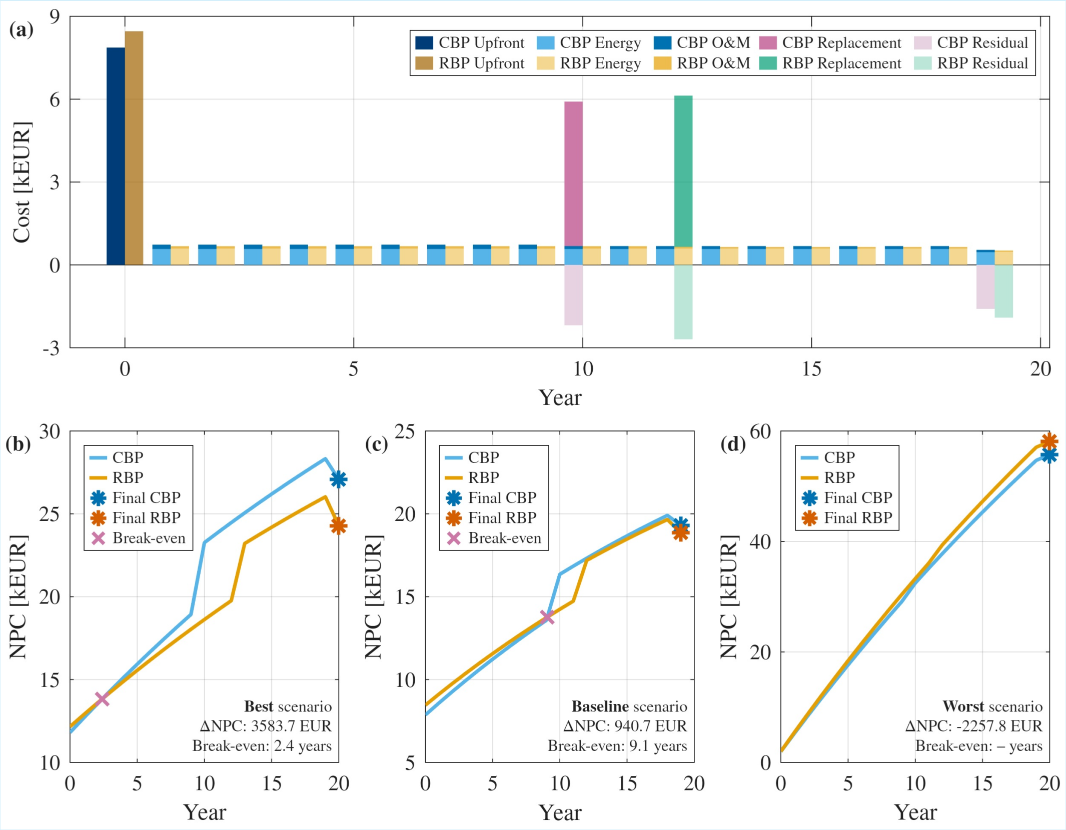

Fig. 4 | Cost composition and cumulative net present cost trajectories of RBPs relative to CBPs. A positive ΔNPC indicates lifetime cost savings for the RBP relative to the CBP. **(a)** Annual cost breakdown in the baseline scenario. **(b–d)** Cumulative NPC trajectories in the most favourable scenario **(b)**, baseline scenario **(c)**, and least favourable scenario **(d)**. All parameter details are provided in Supplementary Table S5.

[13] Wong, B. Color blindness. *Nat. Methods* **8**, 441 (2011).

MiC-Q6: The authors should carefully check the formatting of mathematical equations, especially the use of upright and italic fonts.

Author response: We thank the reviewer for this observation. We agree that in the original version of the manuscript, minor inconsistencies were present in the typographical formatting of mathematical expressions. The manuscript has been revised to ensure consistent and correct mathematical notation throughout. Representative examples of corrections made are shown below:

Ageing rate function subscripts:
Before: $k_{\mathrm{SOC,Q}}$, $k_{T,\mathrm{Q}}$
After: $k_{\mathrm{SOC,Q}}$, $k_{\mathrm{T,Q}}$

C-rate subscript:
Before: $k_{C,\mathrm{Q}}$, $k_{C,\mathrm{R}}$ (italic C, inconsistent with upright DOD)
After: $k_{\mathrm{C,Q}}$, $k_{\mathrm{C,R}}$

Cycle ageing superscripts in NMC equations:
Before: $\widetilde{Q}_i^{l,cyc}$ (italic, inconsistent with LFP formulation)
After: $\widetilde{Q}_i^{l,\mathrm{cyc}}$

Start and end superscripts:
Before: t^{s}_{cyc}, t^{e}_{cyc}
After: $t^{\mathrm{s}}_{\mathrm{cyc}}$, $t^{\mathrm{e}}_{\mathrm{cyc}}$

O&M notation:
Before: C^{OM} in the NPC summation equation (13) and $C^{\mathrm{O\&M}}$ in the O&M cost equation (23)
After: $C^{\mathrm{O\&M}}$ used consistently throughout

Code availability remark

The reproducibility of the figures is great, and it would be even better to open-source the modeling part (I guess the authors intended to do so when the paper can be considered to proceed with publication).

Author response: We thank the reviewer for this suggestion and for the positive assessment of figure reproducibility.

As detailed in our response to MaC-Q3 above, the complete repository, comprising both the simulation framework and the post-processing scripts with raw simulation data, is publicly available at:

https://github.com/askegro/NCOMMS-25-71405-T_Code

2 Reviewer #2 Comments

I co-reviewed this manuscript with one of the reviewers who provided the listed reports. This is part of the Nature Communications initiative to facilitate training in peer review and to provide appropriate recognition for Early Career Researchers who co-review manuscripts.

Author response: We thank you for taking the time to carefully review our manuscript and for your contribution to the peer-review process. Based on your insightful comments, we have revised the manuscript accordingly, as detailed in responses to each of your points above.

3 Reviewer #3 Comments

The manuscript proposes a systematic evaluation of dynamic reconfiguration technology for EV battery packs. In particular, a simulative approach based on empirical aging model and Monte Carlo simulations is used to assess the capability of dynamic reconfiguration technology to extend battery lifetime and increase residual value. The analysis investigates the sensitivity of lifetime extension from several factors, i.e. battery chemistry (LFP or NMC), temperature distribution, cell manufacturing variability and battery pack voltage. In addition, the manuscript provide also a basic economic feasibility analysis. The manuscript is practical and interesting, however it presents some minor issues that should be addressed to improve flow linearity and the overall readability of the manuscript.

Author response: We thank the reviewer for the careful reading of the manuscript and for the positive assessment of its relevance and practical interest. We agree with the reviewer's assessment that, in its original form, the manuscript exhibited minor issues related to flow linearity and overall readability, particularly in the organisation of background information, methodological guidance, and figure presentation.

In response, we have addressed these points throughout the revised manuscript. Specifically, we improved the logical flow by (i) clarifying the methodological workflow already in the Introduction, (ii) restructuring selected subsections to place contextual information before result interpretation, and (iii) enhancing figure readability and self-consistency through clearer structuring, captions, and explicit textual guidance in the Results section. In addition, we removed elements that could be perceived as out of scope to ensure full consistency between figures and the manuscript narrative.

These revisions are purely structural and presentational and do not affect the results or conclusions of the study. We believe these revisions have improved clarity, readability, and coherence of the manuscript.

Q1: In particular, a concise description of the method employed (in particular the simulation model used and the workflow) should be included in the introduction to provide the user with the basic information needed to better understand workflow of the study.

Author response: We thank the reviewer for this helpful suggestion. The reviewer is correct that the original Introduction focused primarily on motivation, literature context, and contributions, while the description of the simulation model and workflow was deferred to later sections. We agree that providing a concise description of the approach in the Introduction improves readability and helps readers better understand the overall workflow.

In the revised manuscript, we have integrated a concise methodological description directly into the final paragraph of the Introduction. Specifically, we now clarify that the analysis is based on experimentally validated battery models (equivalent-circuit electrical models coupled with semi-empirical ageing models), evaluated over a structured design space spanning battery chemistries, pack topologies, usage conditions, and temperature distributions. We further specify that each scenario is assessed using large-scale Monte Carlo simulations with stochastic variations in initial capacity and resistance to capture cell-to-cell manufacturing variability, enabling systematic comparison between

conventional fixed-configuration battery packs (CBPs) and reconfigurable battery packs (RBPs). In addition, we explicitly state that the resulting lifetime gains are subsequently translated into economic outcomes through a system-level cost analysis.

This revision provides a clearer high-level overview of the modelling approach and workflow, while avoiding duplication of the detailed formulations presented in the Methods section. For the reviewer's convenience, the updated last paragraph of Introduction is provided below:

> To address this critical gap, we present a systematic and battery technology-agnostic evaluation of the lifetime extension and economic implications enabled by dynamic reconfiguration. The analysis builds on large-scale virtual cell populations and experimentally validated battery models, combining equivalent-circuit electrical formulations with semi-empirical ageing models. A structured design-space exploration is conducted across a broad range of battery chemistries, pack topologies, usage intensities, and temperature distributions, reflecting the scale and diversity encountered in real-world EV deployments. To account for cell-to-cell manufacturing variability, each scenario is further evaluated using 1,000 Monte Carlo simulations with stochastic variations in initial capacity and resistance, enabling systematic comparison between CBPs and RBPs. Notably, these results reveal that the achievable lifetime extension from dynamic reconfiguration scales non-linearly with system voltage and is significantly influenced by battery chemistries. Building on these findings, we further quantify how lifetime gains translate into economic benefits through a system-level cost analysis, enabling identification of design, usage, and cost thresholds for economically viable deployment. Our results highlight substantial economic opportunities of RBPs, particularly for high-voltage and high-capacity EVs.

Q2: The second paragraph of subsection 2.1 ("Manufacturing inconsistencies ... unattainable") provides basic information that would be more appropriately placed at the beginning of the subsection rather than in its current position.

Author response: We thank the reviewer for this constructive suggestion. The reviewer correctly notes that the paragraph beginning with "Manufacturing inconsistencies . . . unattainable" provides general background information that serves as contextual motivation rather than interpretation of results, and that its current placement may interrupt the logical flow of the subsection. While the original placement was intended to connect the simulation results to real-world manufacturing practice, we acknowledge that introducing this context earlier improves readability and narrative coherence.

Accordingly, we have revised the manuscript by moving this paragraph to the beginning of Subsection 2.1 (Effect of Manufacturing Variability), where it now precedes the discussion of the simulation results and Fig. 3(a). No technical content has been altered; the change is purely structural and improves clarity and logical progression.

Q3: It could be useful to enhance manuscript readability, to improve figures clarity and self-consistency. As an example, subplots of Figure 5 could be grouped in two or more different figures to enhance readability.

Author response: We thank the reviewer for this helpful suggestion. In the revised manuscript, the original Fig. 5 has been split into two separate figures, now presented as Figs. 4 and 5. Specifically, Fig. 4 presents the cost composition and cumulative net present cost trajectories under favourable, baseline, and unfavourable scenarios (former panels (a)–(d)). Fig. 5 presents the sensitivity analysis, global parameter ranking, design-space assessment, and chemistry-dependent outcomes (former panels (e)–(j)). For the reviewer's convenience, the updated figures are provided below.

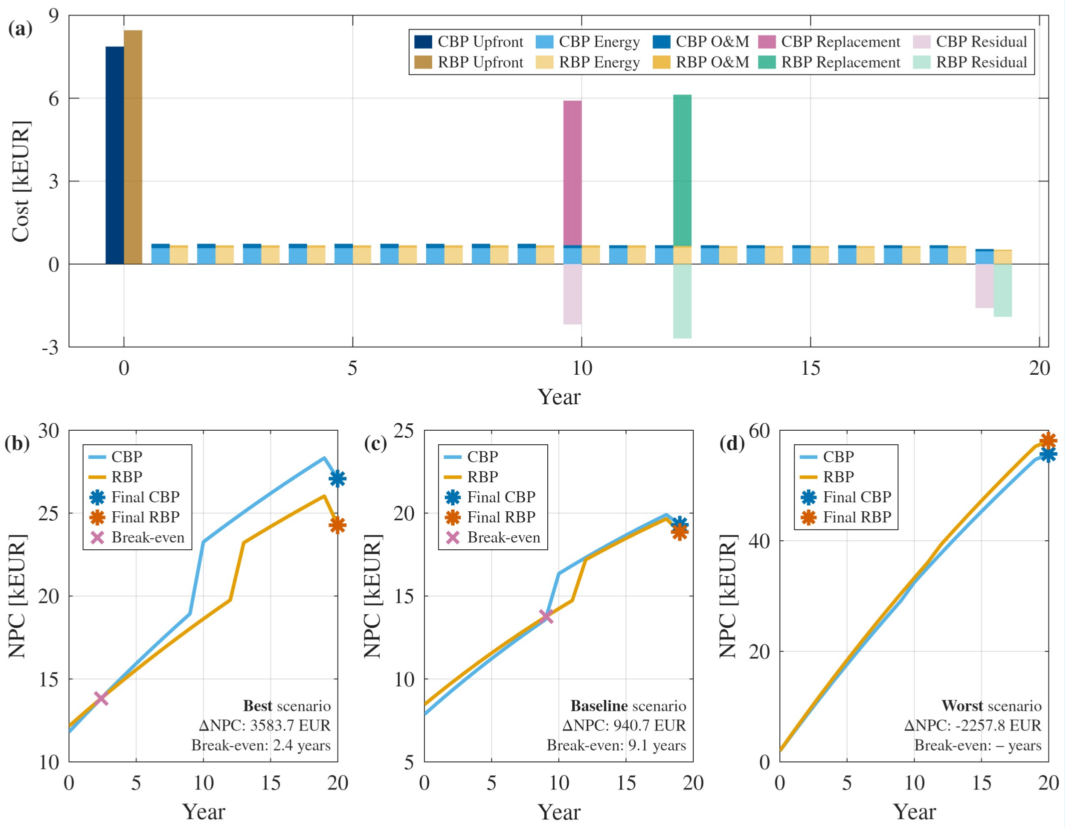

Fig. 4 | Cost composition and cumulative net present cost trajectories of RBPs relative to CBPs. A positive ΔNPC indicates lifetime cost savings for the RBP relative to the CBP. **(a)** Annual cost breakdown in the baseline scenario. **(b–d)** Cumulative NPC trajectories in the most favourable scenario **(b)**, baseline scenario **(c)**, and least favourable scenario **(d)**. All parameter details are provided in Supplementary Table S5.

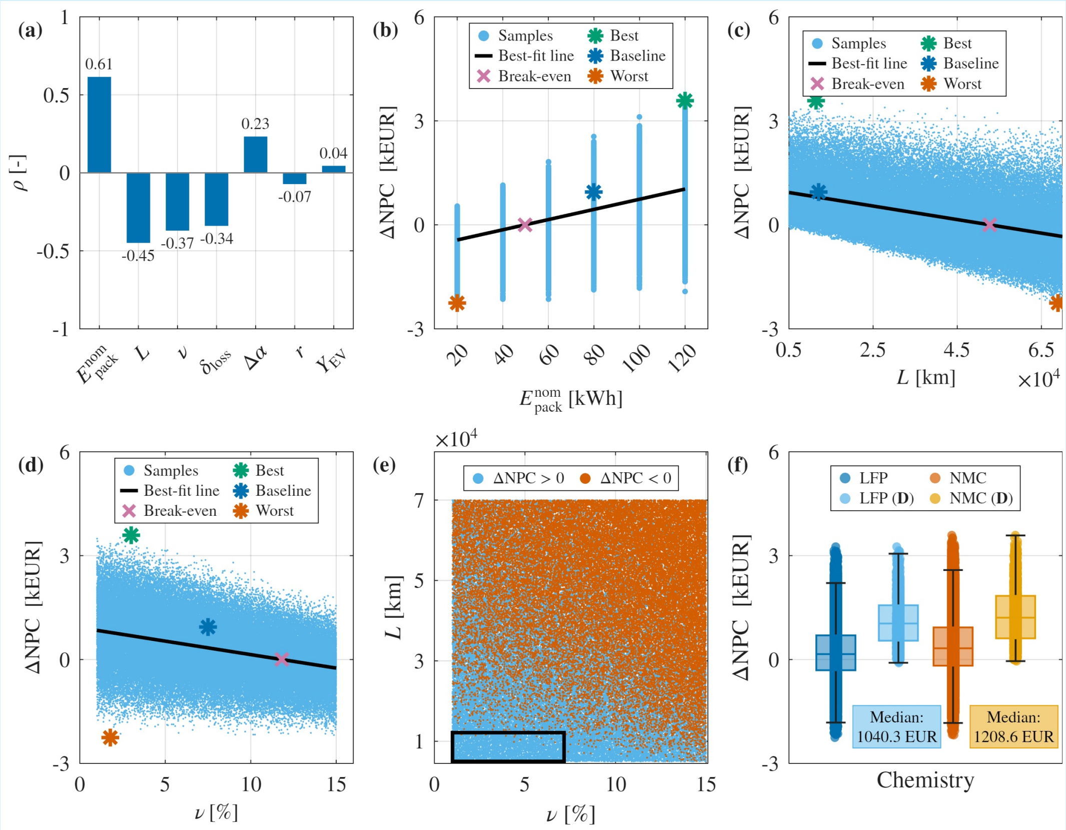

Fig. 5 | Sensitivity analysis of the lifetime cost benefit of RBPs (ΔNPC). **(a)** Global sensitivity ranking based on the Spearman correlation coefficient (ρ) between input parameters and ΔNPC. ν denotes the RBP upfront cost factor. **(b–d)** One-dimensional sensitivities of ΔNPC to nominal battery pack energy ($E_{\text{pack}}^{\text{nom}}$) **(b)**, annual driving distance (L) **(c)**, and relative increase in ohmic losses (δ_{loss}) **(d)**. Points show simulation samples. **(e)** Design space analysis in the (ν, L) plane. The identified rectangular region (D) indicates the favourable design region where at least 99.7% of cases yield $\Delta\text{NPC} > 0$. **(f)** Sensitivity to cell chemistry. Boxplots show the distribution of ΔNPC for LFP and NMC chemistries; 'D' refers to samples within the favourable design region from panel (e).

Q4: Subfigure (g) of Figure 2 suggests an environmental impact analysis that is not discussed in the current manuscript. For consistency, the authors should either include it in the manuscript or remove the subfigure.

Author response: We thank the reviewer for pointing out this inconsistency. The reviewer is correct that subfigure (g) implied a quantitative environmental impact analysis not present in the manuscript, and we agree that it should be removed.

After careful consideration, we concluded that the most coherent solution was to remove Fig. 2 in its entirety rather than just subfigure (g). The remaining subfigures added limited informational value beyond what is already conveyed in the prose of Section 2 and in Supplementary Table S2, which consolidates the definitions, parameter ranges, and scenario counts for all five influencing factors. Removing the figure in full also improves the narrative flow of Section 2, which now opens directly with the analysis rather than

with a design-space overview figure.

4 Reviewer #4 Comments

General

Interesting and important topic that fits within the scope of Nature Communications. However, the written version lacks scientific rigor, which is evident in the written version (compare Section Manuscript Presentation) and in the unclear description of the methodological approach, which raises questions (compare Section Methods). In addition, the interpretations of some results are contradictory (compare Section Results).

Author response: We thank the reviewer for the careful and critical evaluation of the manuscript and for recognising the relevance of the topic within the scope of Nature Communications. We take the concerns regarding scientific rigour, methodological clarity, and result interpretation seriously. In the revised manuscript, we have therefore substantially strengthened clarity and rigour, as detailed in the point-to-point responses below. We are grateful for the reviewer's insightful comments, which have helped improve the clarity and overall presentation of the manuscript.

Manuscript presentation (MP)

MP-Q1: Past-Tense in the results and methodology chapters

Author response: We thank the reviewer for pointing out this stylistic inconsistency. The reviewer is correct that portions of the Results and Methods sections were written using the past tense, which is inconsistent with standard conventions for describing models, assumptions, and analytical procedures. In the revised manuscript, we have reviewed all sections and have revised the tenses to ensure consistency (as highlighted in cyan). Representative examples of the corrections are shown below:

Section 2 (Results), opening paragraph:
Before: "Five key categories of influencing factors **were considered** $\cdots$. Manufacturing variability **was captured** by introducing statistical distributions."
After: "The analysis **considers** five categories of influencing factors $\cdots$. Manufacturing variability **is characterised** by cell-to-cell dispersion."

Section 2.1 (Effect of Manufacturing Variability):
Before: "we **compared** two representative test cases derived from experimental data."
After: "we **compare** two representative manufacturing-variability test cases."

Section 4.1 (Modelling of Battery Cell Behaviour):
Before: "Two cell types **were selected** for evaluation."
After: "Two cell types **are selected** for evaluation."

MP-Q2: Figures are too large, have different font sizes, and descriptions under the figures are too long. Most of the subfigures of Fig. 2 don't have any meaning. I propose replacing Fig. 2 by a short section in the results chapter and a much more condensed figure.

Author response: We thank the reviewer for this constructive suggestion. In the updated manuscript, we have revised the figure presentation to improve clarity, consistency, and information density across the manuscript.

Specifically, following the reviewer's recommendation, the original Fig. 2 has been removed. The information it conveyed has been reorganised into a concise paragraph in the Results section (see excerpt in the box below) and a structured summary in Supplementary Table S2. In addition, the original Fig. 5 has been reorganised and split into two figures, now presented as Figs. 4 and 5. Further revisions to Figs. 1, 3, and 4 are addressed in response to comments MP-Q3, MP-Q7 and MP-Q9, respectively.

> We evaluate the potential of dynamic battery reconfiguration across a representative set of realistic operating conditions and design scenarios. The analysis considers five categories of influencing factors: manufacturing variability, thermal environment, usage pattern, system voltage, and cell chemistry. Manufacturing variability is characterised by cell-to-cell dispersion in beginning-of-life (BOL) capacity and resistance. Thermal environment is characterised by the long-term mean cell temperature and the degree of spatial temperature non-uniformity within the pack. Usage pattern is described by the fraction of time the battery remains at rest, system voltage by the number of series-connected cells, and cell chemistry by separate simulations of lithium iron phosphate (LFP) and lithium nickel manganese cobalt oxide (NMC) cells. Together, these factors span practically relevant EV design and operating regimes and are selected based on their relevance to battery pack design [24], correspondence to real-world usage [25], and physical links to dominant degradation mechanisms [26]. The definitions, symbols, investigated values, and scenario counts for these factors are summarised in Supplementary Table S2.

MP-Q3: In Fig. 5a, the colors are indistinguishable when printed out. Additionally, mixing red and green in one plot is bad practice because of red-green weakness.

Author response: We thank the reviewer for this important observation. The colour scheme across all figures in the revised manuscript has been updated to use a colour-blind-safe palette following the recommendations in [1], replacing the original red–green combination. In particular, the revised palette for Fig. 4 (that includes former Fig. 5a) uses blue tones for CBP cost components and orange tones for RBP cost components. For the reviewer's convenience, the updated figure is provided below.

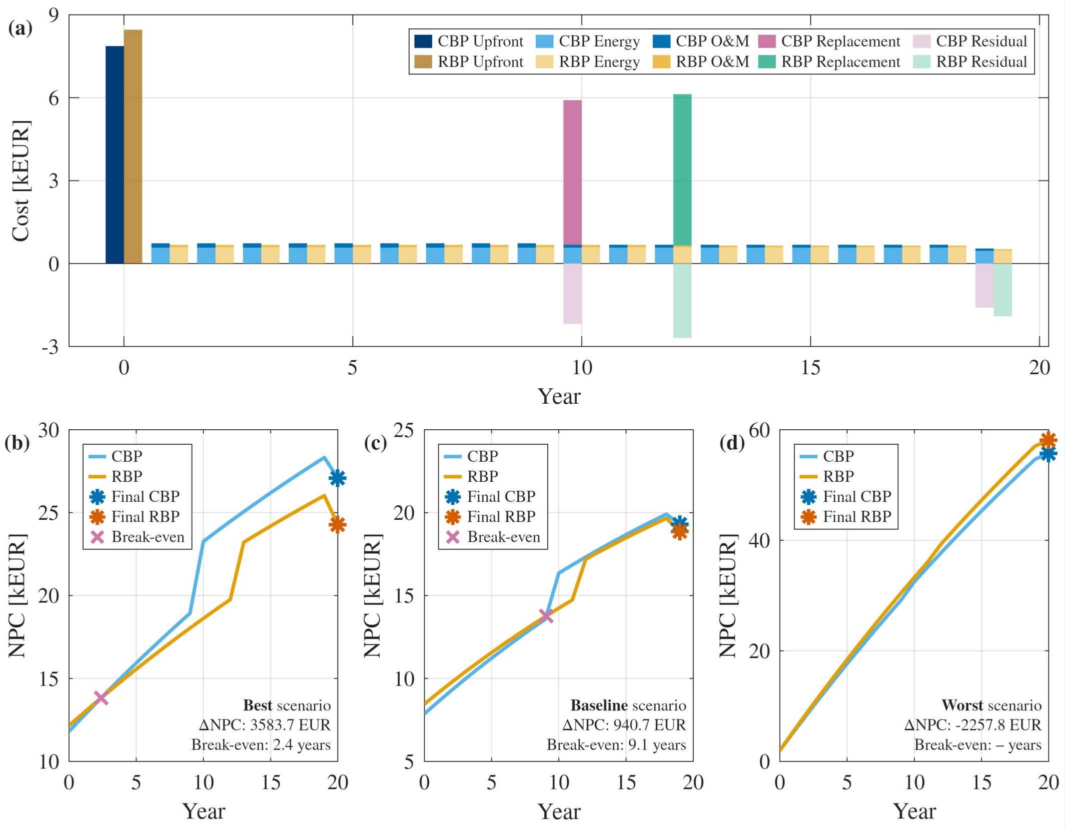

Fig. 4 | Cost composition and cumulative net present cost trajectories of RBPs relative to CBPs. A positive ΔNPC indicates lifetime cost savings for the RBP relative to the CBP. **(a)** Annual cost breakdown in the baseline scenario. **(b–d)** Cumulative NPC trajectories in the most favourable scenario **(b)**, baseline scenario **(c)**, and least favourable scenario **(d)**. All parameter details are provided in Supplementary Table S5.

[1] Wong, B. Color blindness. *Nat. Methods* **8**, 441 (2011).

MP-Q4: I don't see a large benefit in the analysis of Section 2.6, as all related results can already be derived from the previous sections. Instead, it would be much more beneficial to run additional sensitivity studies on the following matters:

- Full analysis NMC
- More conservative approach regarding energy prices, resale value of degraded RBP's, and energy consumption
- While reviewing the code, it is now clear why Section 2.6 is necessary. However, it is not well explained in the text. Additionally, relying solely on the battery voltage seems to be counterintuitive

Author response: We thank the reviewer for this insightful comment regarding the role and interpretation of Section 2.6. We agree that the motivation of Section 2.6 was not sufficiently explicit in the original manuscript. In the revised version, we have clarified its role by explicitly distinguishing it from the preceding factor-wise analyses. Specifically,

while the individual dependencies on manufacturing variability, thermal environment, usage pattern, and chemistry are analysed separately in Sections 2.1–2.5, their combined system-level implications cannot be directly inferred. Section 2.6 addresses this gap by synthesising these factors and projecting the resulting lifetime-extension behaviour onto representative real-world EV architectures. This enables an application-level interpretation of reconfiguration benefits. The revised text is reproduced below for the reviewer's convenience:

> *2.6 Implications for Real-World EV Applications*
>
> While the individual dependencies on manufacturing variability, thermal environment, usage pattern, and chemistry are analysed separately in Sections 2.1–2.5, their combined system-level implications cannot be directly inferred. Here, we synthesise these factors by projecting the resulting lifetime-extension behaviour onto real-world EV architectures, thereby enabling an application-level interpretation of reconfiguration benefits. Specifically, the expected lifetime extension through dynamic reconfiguration is mapped onto 20 representative EV models spanning 15 manufacturers.

In terms of NMC analysis, these results were already included in the original submission. Based on the reviewer's comment, we have revised the corresponding descriptions in Section 2 to improve the clarity and accessibility. The updated text is shown below:

> **2. Results**
>
> ⋮
>
> $\cdots$. Fig. 2 presents the LFP results together with LFP–NMC comparisons, while detailed NMC-only results are provided in Supplementary Fig. S2. $\cdots$.
>
> ⋮
>
> The results, shown in Fig. 3, reveal a clear and interpretable pattern: $\cdots$. $\cdots$, NMC-based vehicles consistently outperform their LFP-based counterparts. This is consistent with earlier findings (Section 2.5), reflecting the stronger reconfiguration gains achievable in chemistries with greater intrinsic ageing variability.

Regarding the suggested additional sensitivity studies, analyses with respect to electricity price and energy consumption have been performed and are reported in Supplementary Fig. S5. Details are also provided in our responses to M-A-Q5 and M-A-Q6 below, and are not repeated here for brevity. Regarding resale value of degraded RBPs, the conceptual validity of the residual value assumption, including the implications of cell-level disassembly workflows for second-life valuation, is addressed in detail in our response to M-A-Q4 below. A dedicated paragraph has been added to the Discussion section accordingly.

Finally, we clarify that system voltage is not treated as the sole determining factor in Section 2.6, but rather as a compact system-level proxy that captures multiple coupled design attributes, including the number of series-connected cells, pack power level, and the statistical averaging of cell-to-cell heterogeneity. This interpretation has now been

made explicit in the revised manuscript to avoid the impression that voltage alone governs the observed trends. The added text is reproduced below:

> Specifically, the expected lifetime extension through dynamic reconfiguration is mapped onto 20 representative EV models spanning 15 manufacturers. These vehicles, characterised by their nominal battery pack voltages and cell chemistries, represent a cross-section of mild hybrid electric cars, low-voltage electric cars, high-voltage electric cars, and electric commercial trucks. System voltage is adopted as a compact system-level proxy that reflects multiple coupled design attributes, including the number of series-connected cells, pack power level, and the statistical averaging of cell-to-cell heterogeneity. This abstraction captures the dominant scaling effects of battery pack configuration without introducing a high-dimensional design space.

MP-Q5: A discussion chapter dealing with potential flaws of such a system or different solutions (e.g., selling the car with a larger gross battery size) to the problem is completely missing.

The functionality of such systems is only briefly mentioned on two separate occasions.

Author response:

We thank the reviewer for this important observation. We agree that the original manuscript lacked a dedicated discussion of potential challenges and alternative engineering approaches to the same underlying problem. We have addressed both points and the functionality of reconfigurable battery packs in the revised manuscript.

Regarding potential challenges, we have clarified these points in the updated Introduction section and Supplementary Information. In particular, while dynamic reconfiguration provides structural flexibility to mitigate cell-to-cell heterogeneity, it introduces additional hardware and system-level considerations. These include increased component count (e.g., switching elements), associated integration effort, and higher upfront cost. Moreover, reconfiguration can lead to additional ohmic losses depending on switching states and current routing. These aspects are now acknowledged in the revised manuscript, with a structured summary of both advantages and associated challenges provided in Supplementary Table S1.

> **1. Introduction**
>
> ⋮
>
> · · · . Nevertheless, these benefits come at the cost of increased hardware complexity, integration effort [10], and upfront costs, factors that have contributed to skepticism about their broader practical viability [11]. A summary of key functional advantages and associated challenges is given in Supplementary Table S1.
>
> ⋮
>
> **S1. Functional Properties, System-Level Outcomes, and Industrial Engagement**

Supplementary Table S1 and Supplementary Fig. S1 complement Fig. 1 of the main text by summarising the functional properties, system-level outcomes, and challenges of dynamic battery reconfiguration, alongside a timeline of industrial engagement.

Supplementary Table S1 | Functional properties, system-level outcomes, and associated challenges of dynamic battery reconfiguration. Functional properties describe capabilities enabled directly by reconfiguration; system-level outcomes are the resulting benefits at the pack or vehicle level; challenges represent costs and drawbacks introduced by the additional hardware and control complexity.

⋮

Regarding the functionality of RBPs, we note that the operating principle is described in the Introduction section (cell bypass and engagement to mitigate weak-cell bottlenecks) and illustrated in Fig. 1, which shows the hardware architecture at cell level. The Method section further specifies the building block explicitly. We believe this level of description is appropriate for a system-level assessment targeting a broad scientific audience, and we have added a paragraph in the revised Discussion to explain the functional difference from alternative design strategies (see Section 3.3 and the box below).

Regarding alternative solutions, we have added a dedicated subsection in the Discussion (Section 3.3) that compares dynamic reconfiguration with other engineering approaches, including battery oversizing, cell sorting, balancing strategies, and modular replacement. This subsection positions these approaches according to their stage of intervention and underlying mechanism, and clarifies how dynamic reconfiguration differs in addressing heterogeneity at the architectural level. For reviewer's convenience, it is reproduced below:

3.3 Alternative Design Strategies

Several engineering approaches have been proposed to mitigate the pack-level consequences of cell-to-cell heterogeneity and premature failure, acting at the stages of manufacturing, design, operation, or maintenance. However, these approaches intervene through fundamentally different mechanisms, and therefore should not be regarded as equivalent solutions to the same problem.

At the manufacturing stage, tighter cell sorting and grading aim to reduce BOL dispersion in capacity and resistance before pack assembly. This can improve initial uniformity, but, as discussed in Section 2.1, it requires additional processing and cannot prevent cell states from diverging progressively during operation. At the design stage, battery oversizing mitigates premature EOL by introducing excess nominal capacity. Although straightforward to implement, this strategy increases material use and pack mass, while leaving the intrinsic weak-cell sensitivity of a fixed series-connected architecture unchanged.

At the operation stage, conventional battery management strategies such as pass-

ive or active balancing can redistribute charge and reduce short-term SOC imbalance [5]. Yet these strategies still act within a fixed electrical topology. They may smooth transient non-uniformity, but they cannot remove the structural bottleneck imposed by weak or degraded cells, nor can they reconfigure current paths in response to evolving cell-level limitations. Maintenance-stage interventions, such as modular replacement, restore performance only after degradation has already become sufficiently pronounced to justify service action.

Dynamic reconfiguration is distinct because it addresses heterogeneity through topology itself. Rather than only reducing the symptoms of non-uniform ageing, it changes how non-uniformity is propagated, tolerated, and managed at pack level during operation. ⋯

MP-Q6: Chapter 4.1 would benefit from more precise citations. It is not clear from which semi-empirical model your model is derived, and what your own contribution is.

Author response: We thank the reviewer for this important comment regarding model provenance and attribution. The reviewer is correct that, in the original manuscript, the lineage of the semi-empirical ageing model and the distinction between adopted formulations and the authors' contributions were not sufficiently clear. In the revised version, we provide precise citations for each component (OCV, ECM, and ageing models) for both LFP and NMC chemistries. The contribution of this work does not lie in the development of new cell-level models, but in the system-level integration of established models to evaluate reconfigurable battery pack architectures. The relevant text in the revised manuscript is shown below:

The electrical and ageing models are adopted from experimentally validated formulations reported in the literature, without re-identification of parameters. Detailed parameter values are provided in Supplementary Information S10. ⋯. The OCV and ECM parameters are taken from [46] for LFP cells and [47, 48] for NMC cells.

The ageing model captures two primary degradation mechanisms: capacity fade and resistance increase. Both components are modelled separately for LFP and NMC chemistries using semi-empirical expressions derived in [49–51].

⋮

For LFP cells, calendar ageing is modelled as a function of the cell's RMS SOC, temperature T, and time t, following the semi-empirical relations proposed in [49] ⋯

The cycle ageing of LFP cells is modelled as a function of the cell's C-rate, depth of discharge (DOD), and equivalent full cycles (EFCs), following [50] ⋯

MP-Q7: Fig. 1 should solely focus on the principle of RPBs. Fig. 1c and b can be summarized in a table; Fig. 1d can be removed.

Author response: We thank the reviewer for this constructive suggestion and agree

that Fig. 1 in its original form was too broad in scope.

In the revised manuscript, Fig. 1 has been restructured to solely present the physical architecture of a reconfigurable battery pack (former panel (a)). The functional properties and system-level outcomes previously shown in panels (b) and (c) have been consolidated into Supplementary Table S1. Panel (d), the industrial engagement timeline, has also been removed from the main figure. It has been retained as Supplementary Fig. S1 for readers who wish to consult it. The Introduction text has been updated accordingly, removing all references to the former panels and replacing by citations to Supplementary Table S1 and Supplementary Fig. S1 where appropriate. For the reviewer's convenience, the updated Fig. 1 and Supplementary Information S1 are provided below.

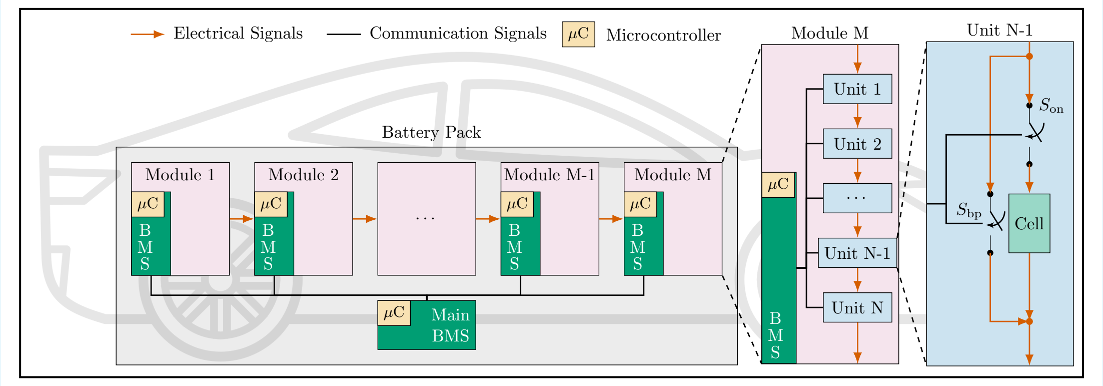

Fig. 1 | Physical architecture of a dynamically reconfigurable battery pack (RBP). Each reconfigurable battery unit consists of a cell and two controllable switches, enabling independent engagement or bypassing during operation. A detailed description of the hierarchical RBP design is provided in Supplementary Information S12.

S1. Functional Properties, System-Level Outcomes, and Industrial Engagement

Supplementary Table S1 and Supplementary Fig. S1 complement Fig. 1 of the main text by summarising the functional properties, system-level outcomes, and challenges of dynamic battery reconfiguration, alongside a timeline of industrial engagement.

Supplementary Table S1 | Functional properties, system-level outcomes, and associated challenges of dynamic battery reconfiguration. Functional properties describe capabilities enabled directly by reconfiguration; system-level outcomes are the resulting benefits at the pack or vehicle level; challenges represent costs and drawbacks introduced by the additional hardware and control complexity.

Functional properties	System-level outcomes	Challenges
State of health balancing	Lifetime extension	Increased ohmic losses
Fault isolation	Faster charging	Increased upfront cost
Improved observability	Improved safety	Hardware complexity
Support for mixed chemistries		Integration effort

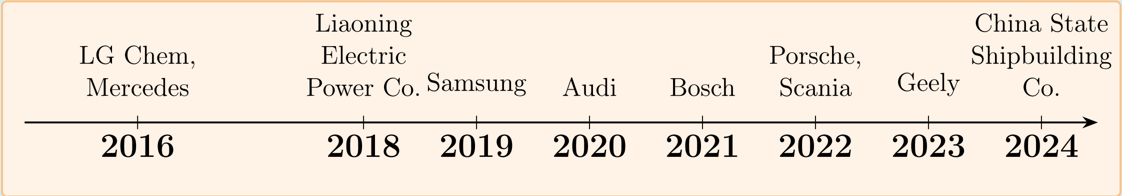

Supplementary Fig. S1 | Timeline of industrial engagement with dynamic battery reconfiguration. First known public disclosures by stakeholders across the automotive, battery manufacturing, grid utility, and heavy industry sectors between 2016 and 2024.

MP-Q8: I don't understand this terminology:

- "while σ_T took values in {0, 5/12, 5/6}°C"
- "The smallest values (N = 4, 14/16)"

Author response: We thank the reviewer for pointing out these instances of unclear terminology. In the revised manuscript, detailed numerical parameter definitions have been removed from the main text and replaced with conceptual descriptions of the thermal environment and system-voltage factors. The corresponding investigated values are now reported exclusively in Supplementary Table S2, which avoids the use of shorthand expressions. Specifically, fractional values such as 5/12 and 5/6 are expressed in decimal form (0.417 and 0.833, respectively). In addition, the chemistry-dependent notation (i.e., "14/16") is now explicitly defined in Supplementary Table S2, where 14 and 16 denote the number of series-connected cells for NMC and LFP, respectively, at approximately 50 V.

MP-Q9: Apply a legend in Fig. 3f

Author response: We thank the reviewer for this helpful observation. A legend has been added directly to panel (f) of Fig. 3 (now Fig. 2 in the revised manuscript), identifying the blue boxes as LFP and the orange boxes as NMC. While this information was previously conveyed in the figure caption, we agree that an in-figure legend improves readability and reduces the caption length, as noted by the reviewer in an earlier comment.

MP-Q10: There is a typo in Formula 19. Parentheses are set incorrectly; However, the formula is correct in the code. Formula 19 should be like this: $c = p_1 \cdot \exp(-p_2 \cdot (y_{init} - y_0)) + p_3$

Author response: We thank the reviewer for carefully checking the equation and for cross-validating it against the code. The reviewer is correct that the intended formulation of Eq. (19) is

$$c = p_1 \cdot \exp\bigl(-p_2 \cdot (y_{\text{init}} - y_0)\bigr) + p_3.$$

In the revised manuscript, we have updated the typesetting of Eq. (19) to make the grouping of terms explicit and unambiguous, ensuring full consistency with the implementation used in the code.

Methods (M)

Assumptions:

M-A-Q1: Although the trend is toward cars that last longer, cars currently only last an average of 10 to 11 years. Accidents can, for example, negate the benefits of a system with high upfront costs.

Author response: We thank the reviewer for raising this interesting point and for requesting clarification regarding typical vehicle service lifetimes.

The statement that passenger vehicles currently exhibit average lifetimes on the order of 10–11 years refers to fleet-level statistics reported for mature automotive markets. For example, recent data indicate an average vehicle age of approximately 11–12 years in Europe [2] and North America [3], reflecting aggregate retirement due to wear, obsolescence, and stochastic events such as accidents.

We agree that accidents and early vehicle retirement can negate the benefits of any technology with higher upfront cost. However, such events are inherently stochastic and exogenous to battery system design, and therefore cannot be addressed through pack-level engineering choices. To enable a consistent and technology-focused comparison, the present analysis evaluates conditional lifetime and cost outcomes under the assumption of continued vehicle operation.

The following paragraph has been added to the Economic Cost Modelling subsection.

> The economic analysis assumes continued vehicle operation and does not explicitly model stochastic vehicle retirement events (e.g., accidents), which are exogenous to battery system design.

[2] European Environment Agency. Evolution of the average passenger car age in the EU between 2013 and 2022. *European Environment Agency* (2024). Available at: https://www.eea.europa.eu/en/circularity/sectoral-modules/product-lifespans/evolution-of-the-average-passenger-car-age-in-the-eu-between-2013-and-2022 (accessed 17 April 2026).

[3] S&P Global Mobility. Average age of vehicles in the US hits 12.8 years in 2025. *S&P Global Mobility* (2025). Available at: https://www.spglobal.com/automotive-insights/en/blogs/2025/05/average-age-of-vehicle-in-us (accessed 17 April 2026).

M-A-Q2: Usage pattern: Employing the usage pattern of a mining hauler sounds arbitrary. There are probably a lot more scenarios between the private case and the mining hauler.

Author response: We thank the reviewer for this comment regarding the representativeness of the selected usage patterns.

The two investigated usage regimes are not intended to represent only "private cars" and "mining haulers" as specific vehicle classes. Rather, they are chosen to span the physical envelope of utilisation intensity relevant to battery ageing. A predominantly idle case ($t_{\text{rest}} = 0.95$) captures calendar-dominated ageing typical of privately used passenger vehicles, which are parked the majority of the time. In contrast, the high-utilisation case ($t_{\text{rest}} = 0.2$) represents an upper-bound scenario in which cycle ageing dominates and idle periods are minimal. A mining-hauler–like duty cycle is used as a realistic proxy for such an extreme continuous-operation regime, as few road-vehicle applications exhibit higher sustained utilisation.

The purpose of selecting these bounding cases is to investigate how dynamic reconfiguration behaves across the full spectrum between calendar-driven and cycle-driven degradation, rather than to exhaustively represent all intermediate usage scenarios. Intermediate operating conditions are physically bounded by these two extremes. We have clarified this rationale in the revised manuscript and explicitly state that the selected usage patterns define a structured sensitivity envelope spanning low- and high-utilisation regimes. The following text has been added at the start of the Effect of Usage Pattern subsection, before the results are discussed.

> The two utilisation regimes span the physical envelope of battery ageing behaviour: the low-utilisation case ($t_{\text{rest}} = 0.95$) is dominated by calendar ageing, while the high-utilisation case ($t_{\text{rest}} = 0.2$) is dominated by cycle ageing. Intermediate duty cycles are physically bounded by these two extremes.

M-A-Q3: As I understand, the switching behavior itself of the RBP is not implemented in the code. This should be stated in the limitations/conclusion section.

Author response: We thank the reviewer for this observation.

The reviewer is correct that the detailed switching dynamics of the reconfigurable battery pack, such as transient electrical behaviour during switching events, switching frequency optimisation, and controller-level decision logic, are not explicitly simulated in the present framework. Instead, the RBP architecture is modelled at a structural level, assuming that reconfiguration decisions are made in a lifetime-oriented manner and that the resulting cell engagement patterns are realised without explicitly resolving fast-timescale switching dynamics. This abstraction allows us to focus on the long-term ageing and techno-economic implications of dynamic reconfiguration across a large design space, while keeping the model computationally tractable.

We agree that real-world implementations would require detailed modelling of switching behaviour, control strategies, and associated losses. The present study therefore represents an upper-bound, system-level assessment of achievable lifetime extension. We have added an explicit statement to the revised manuscript clarifying this modelling as-

sumption and highlighting the need to translate these structural advantages into practical battery management strategies. Furthermore, we note that such strategies should account for switching behaviour (in line with the reviewer's suggestion) and its system-level effects. The relevant content has been added to the Conclusion section and to the Methods section (Statistics of Lifetime Extension subsection), as shown below.

4. Conclusion

⋮

···. Future work should focus on translating the structural advantages of reconfiguration into practical battery management strategies, particularly by accounting for switching behaviour and its system-level effects.

⋮

5.2. Statistics of Lifetime Extension

···. The analysis operates at the structural level, characterising the long-term effects of reconfiguration on cell utilisation and lifetime without resolving fast-timescale switching dynamics or controller-level decision logic.

M-A-Q4: It is questionable if the RBP achieves higher resale values than CBP, as in second-life applications, the cells usually need to be disassembled and tested individually. In this case, it does not matter if all "units" that form the battery pack are aged equally. The effort is potentially the same or even higher when the switches need to be disassembled as well. This should be communicated clearly.

Author response: We thank the reviewer for this important and technically well-founded comment.

In a conventional battery pack (CBP), pack-level end-of-first-life (EOL1) is triggered by the first cell reaching 80% of its nominal capacity, leaving the remaining cells underutilised and resulting in a heterogeneous pack at retirement. In contrast, RBPs balance cell utilisation through selective bypassing, leading to a more uniform degradation state, reduced unused cell capacity, and a more homogeneous and utilisation-efficient starting condition for second-life use. This may increase the technically recoverable value at retirement.

Second-life deployment typically follows either (i) direct pack-level reuse or (ii) cell- or module-level harvesting with testing, grading, and repackaging [4] (Ref. [37] in the revised manuscript). While (ii) is often used to mitigate heterogeneity, it is also driven by external constraints such as certification requirements (e.g., UL 1974 [5] (Ref. [38] in the revised manuscript)), system integration, and liability. RBPs partially relax the heterogeneity constraint through enhanced observability, fault detection, and bypass functionality, enabling continued operation despite localised degradation. This shifts part of the second-life challenge from pre-use sorting to runtime management and can make pack-level reuse (i) more feasible in suitable applications.

We agree with the reviewer that in workflows requiring full disassembly and requalification (ii), additional hardware, such as switches, gate drivers, and interconnections, may result in equal or higher processing effort. The magnitude of this effect depends on system

integration and cannot be neglected a priori.

Overall, the impact on resale value is pathway-dependent. RBPs remove heterogeneity as a key technical driver for disassembly, but do not eliminate external drivers such as certification, integration, and liability constraints. They may increase residual value where pack-level reuse is feasible, but this advantage may be reduced or negated in workflows requiring disassembly, where added hardware can increase processing effort.

We therefore emphasise that the employed economic model does not assume elimination of second-life processing steps. Residual value is represented as a function of retained usable capacity at retirement, and the dominant benefit arises from extended first-life operation rather than second-life value alone. To the extent that RBPs enable pack-level reuse pathways that would otherwise not be viable, the associated value, such as avoided disassembly and repackaging costs, is not explicitly captured in the present framework and may therefore be underestimated.

Based on the above considerations, we have added the following paragraph to the Discussion section of the revised manuscript:

> Second-life deployment pathways vary between direct pack-level reuse and cell-level harvesting followed by sorting and repackaging [37]. RBPs may fundamentally shift second-life strategies from pre-use disassembly and requalification toward in-operation management of degradation. By reducing cell-to-cell heterogeneity and enabling fault-tolerant operation through selective bypassing, they increase the technical feasibility of direct pack-level reuse and open pathways that are currently limited for CBPs. However, the realisation of this potential depends on factors beyond the battery itself, including certification frameworks (e.g., UL 1974 [38]), system integration standards, and liability structures, which may still necessitate conservative processing workflows. As these external constraints are not explicitly modelled, the residual value estimated in this work may underestimate the benefits of RBPs in scenarios where pack-level reuse becomes viable.

[4] R. Ma, S. Tao, X. Sun, Y. Ren, C. Sun, G. Ji, J. Xu, X. Wang, X. Zhang, Q. Wu, et al., Pathway decisions for reuse and recycling of retired lithium-ion batteries considering economic and environmental functions, *Nat. Commun.* 15 (1) (2024) 7641.

[5] Underwriters Laboratories. UL 1974: Evaluation for Repurposing or Remanufacturing Batteries (2023).

M-A-Q5: Although 24 ct/kWh is a valid assumption for the majority of Europe, it would be interesting to do a sensitivity study with higher prices, such as in Germany, as vehicle models are usually produced for Europe and not for specific countries.

M-A-Q6: Fixed energy consumption of 0.2 kWh/km. The energy consumption is dependent on the user behavior and climate conditions. Some users use vehicles mainly for long-range missions (Autobahn → higher consumption) or only in the city (→ lower consumption). It is colder in Winter in Sweden than in Italy. I suggest a sensitivity analysis.

Author response: We thank the reviewer for these constructive suggestions.

To address both points, we have performed additional sensitivity analyses across representative European ranges for both parameters. Electricity price was varied between 0.10 and 0.40 €/kWh (including high-cost markets such as Germany), and vehicle energy consumption between 0.15 and 0.30 kWh/km, consistent with Eurostat [6] and Green NCAP data [7].

For clarity and completeness, the full analysis has been included as a dedicated section in the Supplementary Information (Section S8), as shown below.

S8. Economic Analysis: Sensitivity to Electricity Price and Vehicle Energy Consumption

To assess the robustness of the economic conclusions with respect to regional and usage-dependent variation in energy-related parameters, targeted sensitivity analyses are conducted for both electricity price and vehicle energy consumption. Both parameters enter the economic model through the annual energy cost (see Eqs. 13 and 17), where the cost difference between RBP and CBP is proportional to the product $c^{\mathrm{en}} \cdot \varepsilon \cdot \delta_{\mathrm{loss}}$. Consequently, higher values of either c^{en} or ε scale the ohmic loss penalty proportionally and reduce the net economic benefit of RBPs relative to CBPs. Three representative European levels are evaluated for each parameter: 0.10, 0.24 (baseline), and 0.40 EUR/kWh for electricity price, based on recent Eurostat statistics [2]; and 0.15, 0.20 (baseline), and 0.30 kWh/km for vehicle energy consumption, consistent with real-world testing data reporting a range of 0.158 to 0.312 kWh/km across European electric vehicles [3]. For each scenario, 100,000 parameter combinations are generated using Latin Hypercube Sampling, consistent with the main economic analysis. Pack energy is sampled from the same discrete levels as in the main analysis ($\{20, 40, 60, 80, 100, 120\}$ kWh); the large-pack regime is defined as $E_{\mathrm{pack}}^{\mathrm{nom}} \geq 50\,\mathrm{kW\,h}$, consistent with the break-even threshold identified in Section 2.7, with the first discrete level at or above this threshold being $60\,\mathrm{kW\,h}$. Large packs therefore correspond to $\{60, 80, 100, 120\}$ kWh and small packs to $\{20, 40\}$ kWh. The overall fractions reported below reflect this sampling structure and should be interpreted alongside the pack-stratified results. The results are presented in Supplementary Fig. S5 and summarised in Supplementary Table S6.

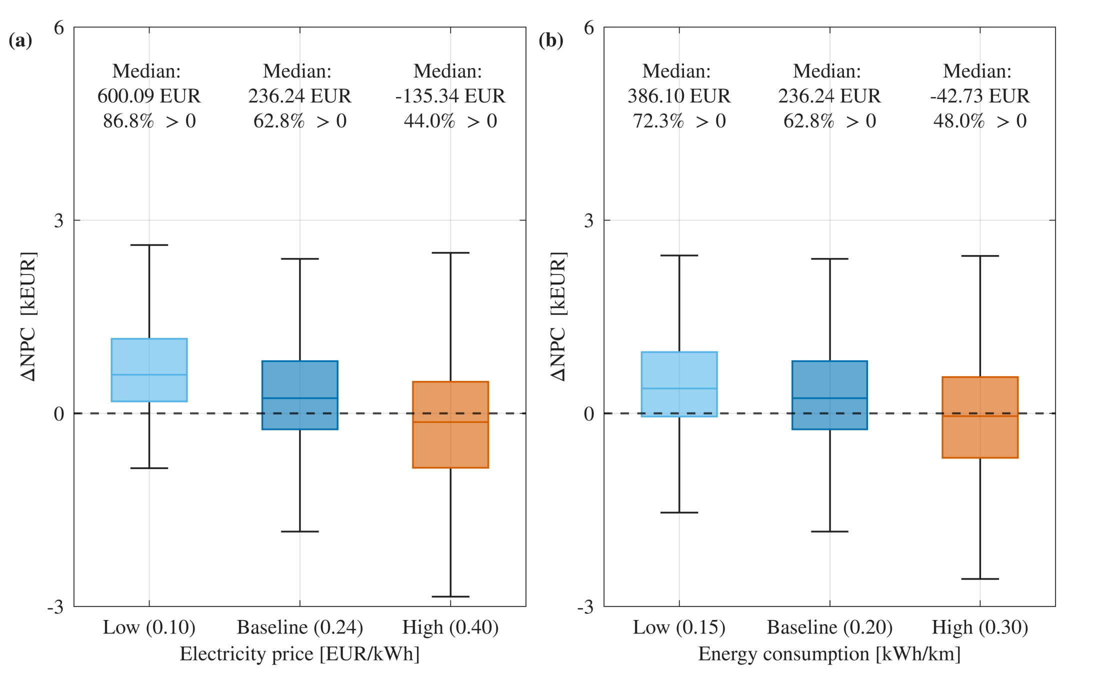

Supplementary Fig. S5 | Sensitivity of the lifetime cost benefit of RBPs (ΔNPC) to electricity price and vehicle energy consumption. Annotated values report the median ΔNPC in kEUR and the fraction of scenarios yielding ΔNPC > 0. The dashed horizontal line marks ΔNPC $= 0$. **(a)** Three representative European electricity price levels: 0.10 €/kWh (lower-cost markets such as Hungary), 0.24 €/kWh (baseline, Sweden [4]), and 0.40 €/kWh (Germany [2]). **(b)** Three representative vehicle energy consumption levels: 0.15 kWh/km (city driving or mild climate), 0.20 kWh/km (baseline, mixed conditions [5]), and 0.30 kWh/km (highway driving or cold-climate winter conditions [3]). All other parameters are varied within the ranges specified in Supplementary Table S12.

Supplementary Table S6 | Summary statistics for the electricity price and vehicle energy consumption sensitivity analyses. Median, 25th percentile (P25), and 75th percentile (P75) of ΔNPC are reported in kEUR. Frac>0 denotes the fraction of all 100,000 sampled scenarios yielding ΔNPC > 0. The final two columns report this fraction restricted to packs of 50 kW h or larger and smaller than 50 kW h, respectively.

⋮

The results show that pack size dominance is conditional on operating conditions rather than universal (Supplementary Table S7). Under low and baseline electricity prices and energy consumption, $E_{\text{pack}}^{\text{nom}}$ is the strongest correlate of ΔNPC, with Spearman coefficients of $\rho = 0.739$ and $\rho = 0.671$ at the low scenarios and $\rho = 0.615$ at both baselines. However, at the high electricity price (0.40 €/kWh), annual driving distance L overtakes pack size as the dominant parameter ($\rho = -0.586$ vs. $\rho = 0.483$). The same crossover occurs at high energy consumption (0.30 kWh/km), where L again becomes dominant ($\rho = -0.560$ vs. $\rho = 0.513$). This reflects the

underlying mechanism: at high electricity prices or energy consumption, the ohmic loss penalty, which scales with $c^{en} \cdot \varepsilon \cdot \delta_{loss} \cdot L$, becomes increasingly sensitive to annual mileage, elevating the influence of L relative to pack size. Notably, δ_{loss} also rises in importance under adverse conditions, entering the top three at both high scenarios ($\rho = -0.439$ at 0.40 €/kWh and $\rho = -0.420$ at 0.30 kWh/km), for the same reason. The parameter ranking under favourable conditions is led by E^{nom}_{pack} and ν, whereas under adverse conditions it shifts to L and E^{nom}_{pack}. This implies that deployment strategy should be conditioned on local electricity price and driving intensity: in low-price markets, pack size and upfront cost are the key levers for economic viability; in high-price or high-consumption markets, annual mileage and switching losses become equally or more important.

Despite this shift in parameter dominance, pack size remains a strong moderating variable across all scenarios. At the most adverse electricity price (0.40 €/kWh), the overall fraction of favourable scenarios drops to 44.0%, but this aggregate masks a pronounced stratification: large-pack configurations retain 57.1% of favourable outcomes while small-pack configurations fall to 17.7%. An analogous pattern holds for energy consumption: at 0.30 kWh/km, the overall fraction is 48.0%, with large packs at 61.7% and small packs at 20.5%. In both cases, the deterioration of economic viability under adverse conditions is therefore concentrated almost entirely in small-pack applications.

Of the two sensitivity parameters, electricity price has the larger effect on ΔNPC. Increasing electricity price from the baseline to 0.40 €/kWh reduces the overall median saving from 236.24 €to −135.34 €and the large-pack favourable fraction from 77.9% to 57.1%. Increasing energy consumption from the baseline to 0.30 kWh/km produces a smaller reduction: median saving from 236.24 €to −42.73 €and large-pack favourable fraction from 77.9% to 61.7%. This asymmetry is consistent with the Spearman results: at the high electricity price scenario, $|\rho(L)| = 0.586$ compared to $|\rho(L)| = 0.560$ at the high energy consumption scenario, confirming that electricity price amplifies the influence of mileage more strongly than energy consumption does (Supplementary Table S7). Conversely, the low electricity price scenario (0.10 €/kWh) reveals substantial upside: 96.4% of large-pack scenarios are favourable with a median saving of 600.09 €, more than double the baseline median. This indicates that in lower-price European markets, the economic case for large-pack RBPs is substantially stronger than the baseline analysis suggests.

Taken together, these results complement the main analysis $\cdots$.

Correspondingly, a new paragraph has been added to the end of Section 2.7 in the revised manuscript to briefly summarise the key results of these analyses.

2.7 Economic Impact of RBPs

⋮

To assess robustness with respect to regional variation in electricity price and

vehicle energy consumption, targeted sensitivity analyses are conducted across three representative European levels for each parameter, with results reported in Supplementary Fig. S5 and Supplementary Table S6. For large-pack applications ($E_{\text{pack}}^{\text{nom}} \geq 50\,\text{kW h}$), the fraction of economically favourable scenarios remains majority-positive across the full range of tested conditions, whereas small-pack viability is more sensitive to both parameters.

[6] Eurostat, Electricity price statistics (2025). Available at: https://ec.europa.eu/eurostat/statistics-explained/index.php?title=Electricity_price_statistics (accessed 2 March 2026).

[7] Green NCAP, Estimated real-world energy consumption and driving range: Test Procedure, Version 1.0.0 (2025). Available at: https://www.greenncap.com/wp-content/uploads/GNT_Estimated-real-world-energy-consumption-and-driving-range_Test-Procedure_v1.0.0.pdf (accessed 2 March 2026).

M-A-Q7: As the battery modeling is described in detail, I suggest also presenting the key facts of the modeled cells: capacity in Ah, rated cycles, etc.

Author response: We thank the reviewer for this suggestion. The key specifications of both modelled cells, including nominal voltage, nominal capacity, terminal voltage limits, and equivalent circuit model parameters, are already reported in Supplementary Tables S8 and S9, together with the OCV–SOC curves in Supplementary Fig. S10. For completeness, a brief reference to these tables has been added to the introduction of the Method section (Page 14 of the revised manuscript):

Cell specifications are provided in Supplementary Tables S8 and S9.

Literature

Similar systems are already available in the stationary market (STABL Energy GmbH). How do such systems compare in price and reliability to conventional systems?

Author response: We thank the reviewer for highlighting existing stationary implementations of reconfigurable battery systems.

Reconfigurable architectures have been commercialised in stationary energy storage, with examples including Relectrify [8], STABL Energy [9] and Rimac Energy's SineStack platform [10], as pointed out by the reviewer. While these examples demonstrate the practical viability of dynamic reconfiguration, a quantitative comparison of price and reliability against conventional stationary systems is not currently possible from public sources, and reliability is typically communicated via operational availability rather than formal metrics such as mean time between failures. Even where data are reported, differences in system boundaries, integration level, and operating conditions limit direct comparability to conventional systems.

We note that stationary battery systems are subject to a layered regulatory framework, including cell and system safety standards (e.g., UL 1973 [11], Ref. [41] in the revised

manuscript), system-level certification (e.g., UL 9540 [12], Ref. [42] in the revised manuscript), installation and fire safety requirements (e.g., NFPA 855 [13], Ref. [43] in the revised manuscript), and repurposing standards (e.g., UL 1974 [5]). These frameworks impose validation, testing, and integration requirements that are not directly reflected in pack-level metrics and may limit the extent to which architectural differences translate into cost or reliability advantages.

Nevertheless, the framework developed in this work is not vendor- or application-specific. It is based on electrochemical ageing, electrical system modelling, and simulation-based evaluation of degradation and utilisation. Application-specific factors, such as duty cycles and thermal conditions, enter through parameterisation, while regulatory and system-level constraints impose boundaries on admissible operation and design rather than altering the underlying model structure. This clarification has been added in the revised manuscript by adding a new subsection in the Discussion section.

3.4 Beyond EV Applications

While the analysis is presented in the context of EV operation, the underlying framework is not specific to a particular application domain. By separating model structure from parameterisation, it can be extended to systems with different duty cycles, power profiles, and operational constraints, including stationary energy storage [39, 40]. In such settings, reconfigurable architectures may provide similar advantages in terms of utilisation efficiency and robustness to cell-level degradation. However, the extent to which these benefits are realised depends on application-specific constraint regimes. Stationary systems, for example, are subject to distinct safety, installation, and certification requirements (e.g., UL 1973 [41], UL 9540 [42], NFPA 855 [43]), as well as different integration and liability considerations.

These factors influence not only system design, but also acceptable operating strategies and failure tolerances, which in turn affect the practical value of reconfiguration. Therefore, extending the present framework to other domains requires careful reparameterisation under domain-specific constraints, rather than direct transfer of EV-based results.

[8] Relectrify, Relectrify second-life battery trial: Final knowledge sharing report (Public), Australian Renewable Energy Agency (ARENA), Project 2020/ARP023, 2025. Available at: https://arena.gov.au/assets/2025/05/Relectrify-Second-life-battery-trial-Final-knowledge-sharing-report.pdf (accessed 17 April 2026).

[9] STABL Energy GmbH, STABL container 20 ft – Datasheet, 2025. Available at: https://143465754.fs1.hubspotusercontent-eu1.net/hubfs/143465754/Website%20Download%20Dateien/EN/stabl-container-20ft-datasheet-2025-12-en.pdf (accessed 17 April 2026).

[10] Rimac Energy, SineStack Battery Energy Storage System, 2024. Available at: https://www.rimac-energy.com/sinestack (accessed 17 April 2026).

[11] Underwriters Laboratories. UL 1973: Standard for Batteries for Use in Stationary, Vehicle Auxiliary Power and Light Electric Rail (LER) Applications (2018).

[12] Underwriters Laboratories. UL 9540: Energy Storage Systems and Equipment

(2023).

[13] National Fire Protection Association (NFPA). NFPA 855: Standard for the Installation of Stationary Energy Storage Systems (2023).

Results (R)

R-Q1: Contradiction in Use-case results

- "These findings underscore that dynamic reconfiguration is particularly well-suited to high-voltage EV architectures. Long-range BEVs, performance-oriented EVs, and commercial electric trucks, where both system voltage and the number of series-connected cells are high, stand to gain the most."
- "Notably, we find that for ν below approximately 7.16% and annual driving distances under 12,150 km, RBPs deliver cost benefits across nearly all tested scenarios."

Electric trucks usually drive more than 100.000 km per year, which diminishes the cost efficiency of RBPs. Additionally, given the 100.000 km per year, the lifetime of Trucks is much shorter than assumed in this publication (ca. 12 years). Although each result might be correct, the findings should be presented clearly and put into context. Since battery packs in trucks are more accessible than in passenger cars, replacing the battery may be a more cost-effective option.

Author response: We thank the reviewer for this insightful observation. The reviewer correctly notes that the manuscript originally juxtaposed two results that could appear contradictory when interpreted without additional context.

The first statement refers to the technical lifetime extension potential of dynamic reconfiguration, which increases with the number of series-connected cells and is therefore particularly pronounced in high-voltage battery architectures such as those used in long-range BEVs and heavy-duty electric trucks.

The second statement refers to the economic outcome, which depends on additional factors such as annual driving distance and operating costs. High utilisation rates increase the cumulative impact of reconfiguration-related losses and may therefore reduce the net economic benefit despite larger achievable lifetime gains.

We have revised Section 2.6 and Section 2.7 to explicitly distinguish between technical lifetime-extension potential and economic attractiveness, thereby clarifying that heavy-duty vehicles may experience large technical benefits while the economic outcome remains usage-dependent. The updated text in the revised manuscript (Page 7) is shown below:

> *2.6 Implications for Real-World EV Applications*
>
> ⋮
>
> These findings underscore that dynamic reconfiguration is particularly well-suited to high-voltage EV architectures, where the large number of series-connected cells amplifies the achievable lifetime extension. Vehicle classes such as long-range BEVs, performance-oriented EVs, and electric commercial trucks typically fall into this

high-voltage regime and therefore exhibit substantial technical benefits in terms of lifetime extension. ⋯

2.7 Economic Impact of RBPs

While Section 2.6 establishes the technical lifetime-extension potential of dynamic reconfiguration, its practical adoption ultimately depends on economic viability. In particular, the extent to which lifetime extension translates into cost benefits depends on usage intensity and economic conditions.

Correspondingly, we have updated the relevant part in the Conclusion section, as shown below:

Our economic analysis reveals that dynamic reconfiguration can deliver significant lifetime cost savings under realistic EV deployment scenarios. These savings stem primarily from deferred battery replacements and improved residual value. However, the economic outcome depends not only on the achievable lifetime extension but also on operational factors. ⋯

R-Q2: When battery packs are bypassed, what effect does it have on the vehicle's range? From a user's perspective, it does not make a difference whether the battery size is reduced due to "balanced aging" or the aging effect.

Author response: We thank the reviewer for raising this important user-level perspective. The reviewer is correct that bypassing degraded cells reduces instantaneous usable pack energy and therefore driving range. From a user perspective, this range reduction is indistinguishable from that caused by capacity fade in a conventional pack.

However, the relevant comparison is not between a new and a late-life RBP, but between an RBP and a conventional fixed-topology pack at the same point in time. In a conventional pack, end of life is triggered the moment a single outlier cell reaches 80% of its nominal capacity, regardless of the state of the remaining cells. The RBP instead bypasses that outlier cell and continues operating on the remaining healthy cells, extending pack life at the cost of a modest, gradual range reduction. The user therefore trades a sharp, premature retirement event for a smoother, more gradual decline in usable range, which is arguably the more user-friendly outcome. Lifetime extension in the RBP thus arises from avoiding premature pack-level failure, not from eliminating capacity fade. This perspective aligns with the reviewer's observation and clarifies the user-level implications of the proposed approach.

Summary

The manuscript presents an interesting and important topic that fits within the scope of Nature Communications. However, there are significant issues with the manuscript's scientific rigor, including the use of past tense in the results and methodology chapters, inconsistencies in figure presentation, and unclear methodological descriptions. The results also contain contradictions, and there is a lack of discussion on potential flaws or

alternative solutions to the problem. The assumptions made in the methods section are questionable, and the literature review could benefit from a comparison of similar systems in the stationary market. The results show contradictions in the use-case results, particularly regarding the cost-efficiency of RBPs in electric trucks. The effect of bypassing battery packs on the vehicle's range is also not addressed.

Overall, the manuscript requires significant revisions to improve its scientific rigor and clarity, including addressing the identified issues in the methods, results, and discussion sections. Additional sensitivity studies and a more thorough literature review are also strongly recommended.

Author response:

We thank the reviewer for this consolidated summary.

All points raised have been addressed in the individual responses above. Specifically, tense consistency has been corrected throughout Sections 2 and 4 (MP-Q1); figure presentation has been substantially revised (MP-Q2, MP-Q3, MP-Q7, MP-Q9); methodological descriptions have been clarified, including modelling scope, switching dynamics, and parameter rationale (M-A-Q1 through M-A-Q4); the contradiction between technical and economic use-case results has been resolved by explicitly distinguishing lifetime-extension potential from economic attractiveness (R-Q1); the effect of cell bypassing on driving range has been addressed (R-Q2); a dedicated discussion of practical limitations and alternative approaches has been added Section 3 (MP-Q5); the applicability of the framework to stationary reconfigurable battery systems has been discussed in Section 3 (Literature comment); and targeted sensitivity analyses with respect to electricity price and energy consumption have been performed and reported in Supplementary Information (M-A-Q5 and M-A-Q6).

We believe these revisions collectively improve the scientific rigour, clarity, and practical relevance of the manuscript.

Code availability remark (CAR)

CAR-Q1: For reproducibility and ease of use it would be beneficial to run the simulations and publish the code in Python. I don't see any reason why a Matlab/Simulink package would be better suited than Python in this case.

Author response: We thank the reviewer for this suggestion. The simulation framework uses MATLAB's Parallel Computing Toolbox for `parfor`-based Monte Carlo parallelisation, though the code runs correctly without it by falling back to a sequential loop. The simulation framework itself does not use Simulink or any other domain-specific toolboxes; the postprocessing scripts additionally require the Statistics and Machine Learning Toolbox for Latin Hypercube Sampling and statistical aggregation. The core numerical routines, equivalent circuit simulation, semi-empirical ageing models, and Monte Carlo sampling, have direct counterparts in standard Python scientific libraries such as NumPy, SciPy, and joblib. A Python reimplementation is therefore feasible in principle, but would require substantial additional effort beyond the scope of the present revision. Hence, we identify it as a valuable direction for future work.

CAR-Q2: The code is executable, however, the presented framework itself is not included in the code (only plots and the economic assessment are included). From the presented results, it is clear, that the technology is sensitive to specific conditions. That is why the framework itselft is needed to test specific configurations for a high impact of the publication.

Author response: We thank the reviewer for this important remark. The reviewer is correct that the originally shared repository contained only post-processing and economic assessment scripts. The repository has now been extended to include the complete simulation framework and is publicly available at:

https://github.com/askegro/NCOMMS-25-71405-T_Code

It is organised into two subfolders: `simulation/` contains the complete MATLAB simulation framework, and `postprocessing/` contains all figure generation and economic analysis scripts together with the raw simulation output data for all 240 scenarios, enabling direct reproduction of all reported figures without rerunning the simulations. Usage instructions for both local and HPC environments are provided in the repository `README.md`.

5 Reviewer #5 Comments

I co-reviewed this manuscript with one of the reviewers who provided the listed reports. This is part of the Nature Communications initiative to facilitate training in peer review and to provide appropriate recognition for Early Career Researchers who co-review manuscripts.

Author response: We thank the reviewer for their careful evaluation of the manuscript and for their participation in the Nature Communications co-review initiative. Based on your insightful comments, we have revised the manuscript accordingly, as detailed in our responses to each of your points and those of Reviewer 4 above.
